# Supplementary material for: Modifying electron injection kinetics for selective photoreduction of nitroarenes into cyclic and asymmetric azo compounds
Source: Nat Commun. 2022 Apr 11;13:1940. doi: 10.1038/s41467-022-29559-z (PMC9001638; doi:10.1038/s41467-022-29559-z)
Supplement: Supplementary file 1 — Supplementary Information [file 41467_2022_29559_MOESM1_ESM.pdf]

Supplementary Information

Modifying electron injection kinetics for selective photoreduction of nitroarenes into cyclic and asymmetric azo compounds

Yang Yang, Xu Jing*, Jing Zhang, Fengyu Yang, and Chunying Duan*

State Key Laboratory of Fine Chemicals, Zhang Dayu College of Chemistry, Dalian University of Technology Dalian, 116024, China

Supplementary Methods

1. Summaries of the mentioned substrates and electron donors.

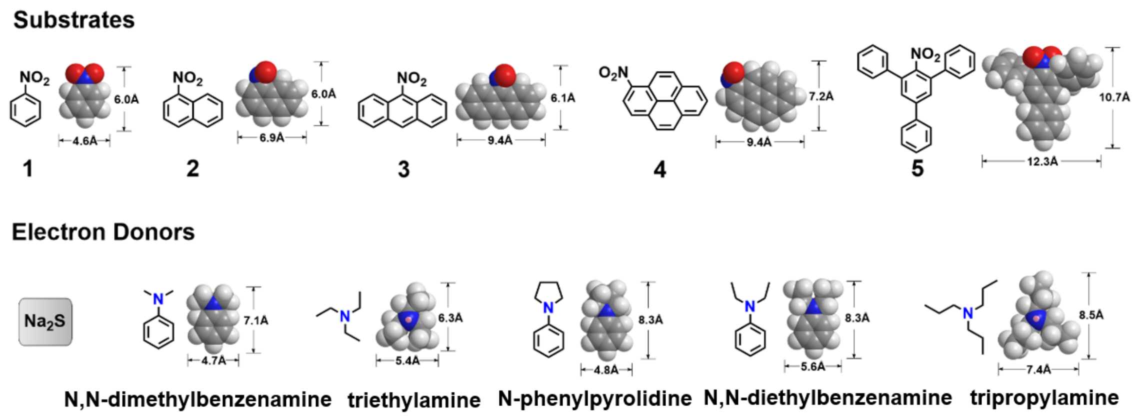

Supplementary Figure 1. Summaries of the mentioned substrates and reductants, and the molecule size measured by theoretical software simulation.

2. Synthesis of dinitro substrates

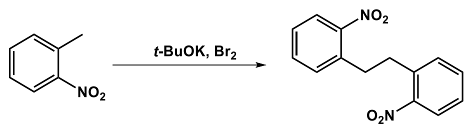

2-nitrotoluene (1.00 g, 7.5 mmol) was dissolved in tetrahydrofuran at 0 °C, followed by addition of potassium butoxide as bases. After 10 min agitation, the reaction mixture was then added bromine (1.60 g, 10.0 mmol) and further stirred for 10 min before addition of 250 mL of ice/water. The precipitate was filtered and the filtrate was extracted with CH_2Cl_2 (3 × 50 mL). The organic layers were combined and washed with saturated sodium thiosulfate solution and saturated sodium. After dried over Na_2SO_4, the solution was concentrated under reduced pressure. The final product 2,2'-Dinitrodibenzyl was purified by crystallization and collected as a white solid.[1]

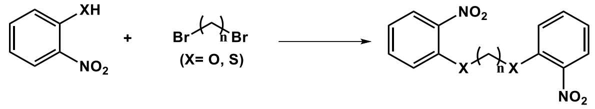

To a solution of o-nitrophenol (1.0 mmol) in DMSO (20 mL), anhydrous potassium carbonate (1.0 mmol) was added in small portion and the reaction mixture was stirred continuously with the inert atmosphere. Then the dibromo substrate (0.5 mmol) was injected into the reaction solution and was further stirred for 2 h. After the completion of the reaction, the mixture was extracted with ethyl acetate (2 × 10 mL) and washed successively with HCl (2 N, 2×5 mL), and cold water. After treated with Na_2SO_4, the finale product was collected by evaporating the solvent under reduced pressure.[2]

3. Crystallography

The intensities were collected on a Bruker SMART APEX CCD diffractometer equipped with a graphite-monochromated Mo-Kα (λ = 0.71073 Å) radiation source; the data were acquired using the SMART and SAINT programs[3, 4]. The structures were solved by direct methods and refined on F^2 by full-matrix least-squares methods using the SHELXTL version 5.1 software[5].

Crystal data for H_1

Crystal data for H_1: $C_{375}H_{401}Co_6F_{24}N_{38}O_{40}P_{16}$ [$C_{312}H_{228}Co_6F_{24}N_{28}O_{12}P_{16} \cdot 10(C_3H_7NO) \cdot 5(CH_3OH) \cdot 7(C_4H_{10}O) \cdot 6H_2O$], Mr = 7406.93, Monoclinic space group C 2/c, dark-red block, a = 27.2433 (8) Å, b = 48.4238 (14) Å, c = 35.3446 (10) Å, α = γ = 90.0°, β = 111.329 (2) °,V = 43424 (2) $Å^3$, Z = 4, Dc= 1.133 g cm^{-3}, T = 173 (2) K, μ (Mo-Kα) = 0.355 mm^{-1}. [R_{int} = 0.1087]. For 38126 unique reflects, final R_1[with $I > 2\sigma(I)$] = 0.0903, wR_2 (all data) = 0.1674, GOOF = 0.960. CCDC NO. 1913942.

For the refinement of H_1, except the solvent molecules, other non-hydrogen atoms were refined anisotropically, hydrogen atoms were fixed geometrically at calculated distances and allowed to ride on the parent non-hydrogen atoms. Two of the benzene rings in the ligands, and several F aoms in the counter PF_6^- ions were disordered into two parts with the site occupied factors of each part being fixed as 0.5, respectively. The related bond distances in the solvent molecules were restrained as idealized values. Thermal parameters on adjacent atoms of several solvent molecules were restrained to be similar.

In the checkcif file, the A alert is caused by the weak diffraction intensity of the poor quality crystal, and the "short inter D…A contact" is caused by the presence of partly occupied solvent molecules with highly disordered atoms. The crystal analysis here could offer the structure of the cage compound and estimate the size of the cavity, which is helpful for the research of the catalytical chemistry of the cage compound.

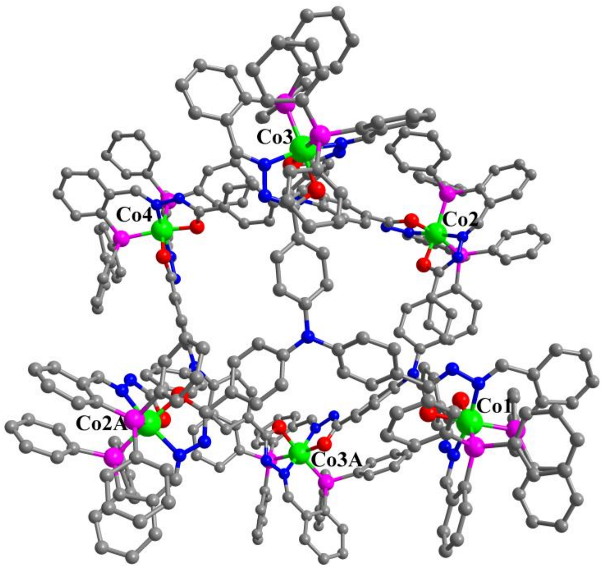

Supplementary Figure 2. Structure of the molecular octahedron H_1, showing the coordination geometries of the cobalt ions. Hydrogen atoms, anions, and solvent molecules are omitted for clarity.

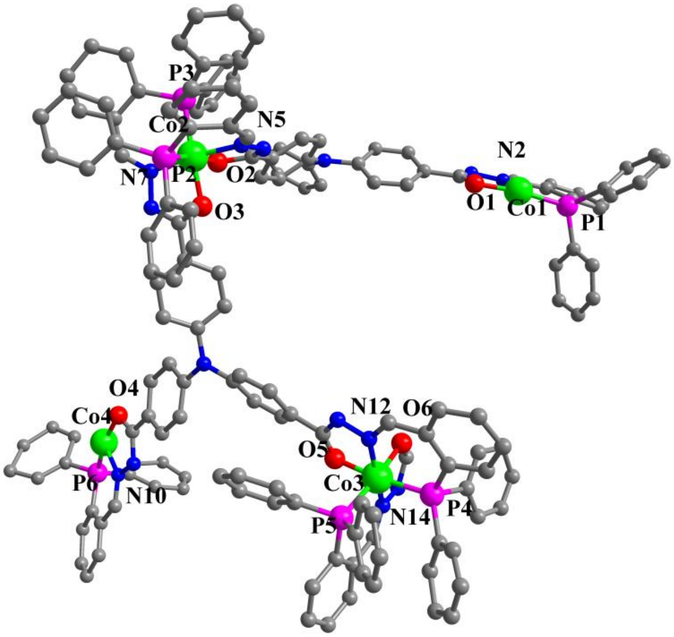

Supplementary Figure 3. Molecular structure of H_1 capsule within a unique asymmetric unit, showing the backbone of the liands in the complex.

Supplementary Table 1. Slective bond distance (Å) in H_1.

	**Bond distance (Å)**		**Bond distance (Å)**
Co1-N2	1.906(9)	Co3-N12	1.905(11)
Co1-O1	1.907(7)	Co3-O6	1.921(7)
Co1-P1	2.232(3)	Co3-O5	1.923(7)
Co2-O2	1.878(9)	Co3-N14	1.924(10)
Co2-O3	1.898(8)	Co3-P4	2.234(4)
Co2-N7	1.914(9)	Co3-P5	2.245(3)
Co2-N5	2.029(11)	Co4-N10	1.897(12)
Co2-P3	2.251(4)	Co4-O4	1.917(7)
Co2-P2	2.249(4)	Co4-P6	2.231(4)

Supplementary Table 2. Slective bond angle (°) in H_1.

	**Bond angle (°)**		**Bond angle (°)**
N2-Co1-O1	84.8(3)	N10-Co4-O4	83.3(5)
N2-Co1-P1	92.1(3)	N10-Co4-P6	90.3(3)
O1-Co1-P1	172.8(2)	O4-Co4-P6	169.0(3)
O2-Co2-N5	84.4(3)	O6-Co3-N12	86.6(4)
O2-Co2-P3	87.8(3)	O6-Co3-O5	85.9(3)
O2-Co2-N7	90.2(4)	O6-Co3-P5	173.7(3)
O2-Co2-P2	170.9(3)	O6-Co3-N14	82.5(4)
O2-Co2-O3	82.1(4)	O6-Co3-P4	86.8(3)
N5-Co2-P3	91.3(3)	N12-Co3-O5	2.231(4)
N5-Co2-N7	173.0(3)	N12-Co3-P5	83.3(4)
N5-Co2-P2	92.94(19)	N12-Co3-N14	165.9(5)
N5-Co2-O3	90.6(4)	N12-Co3-P4	95.6(4)
P3-Co2-N7	92.8(3)	O5-Co3-P5	87.9(2)
P3-Co2-P2	101.01(14)	O5-Co3-N14	87.0(4)
P3-Co2-O3	169.5(3)	O5-Co3-P4	172.6(2)
N7-Co2-P2	91.8(3)	P5-Co3-N14	96.5(3)
N7-Co2-O3	84.4(4)	P5-Co3-P4	99.50(14)
P2-Co2-O3	89.2(3)	N14-Co3-P4	92.6(3)

4. The computational details.

Docking calculations were performed with the AutoDock program 4.2. The cage and Fluorescein molecule were downloaded from the CCDC database. Molecular docking simulations of Fluorescein molecule were performed against cage utilizing AutoDock 4.2. The cage was used as the recipient in the docking calculation. The models of the cage were refined by adding hydrogen atoms, followed by the assignment of Kollman charges, fragmental volumes, and atomic solvation parameters to adhesive by means of AutoDock Tools. For the ligand, Fluorescein molecule was refined by adding hydrogen atoms in a similar manner to that for adhesive. Next, Gasteiger partial charges were assigned to the ligands, and nonpolar hydrogens were merged. All torsions were allowed to rotate during docking. The Lamarckian genetic algorithm was used to determine the appropriate binding positions, orientations, and conformations of the ligands. Default parameters were used, except for the number of runs was set to 100. The blind docking strategy was used with a 54 Å × 66 Å × 68Å (x, y and z, respectively). grid box which ensured sufficient spaced to cover the entire surface of the cage. The Lamarckian genetic algorithm was chosen with default parameters, which was set to 100 for more accurate docking results. The best docking mode of the host–guest complex was chosen based on the binding energy score, clustering, and chemical reasonableness.

5. ESI-MS spectra data of the catalysts

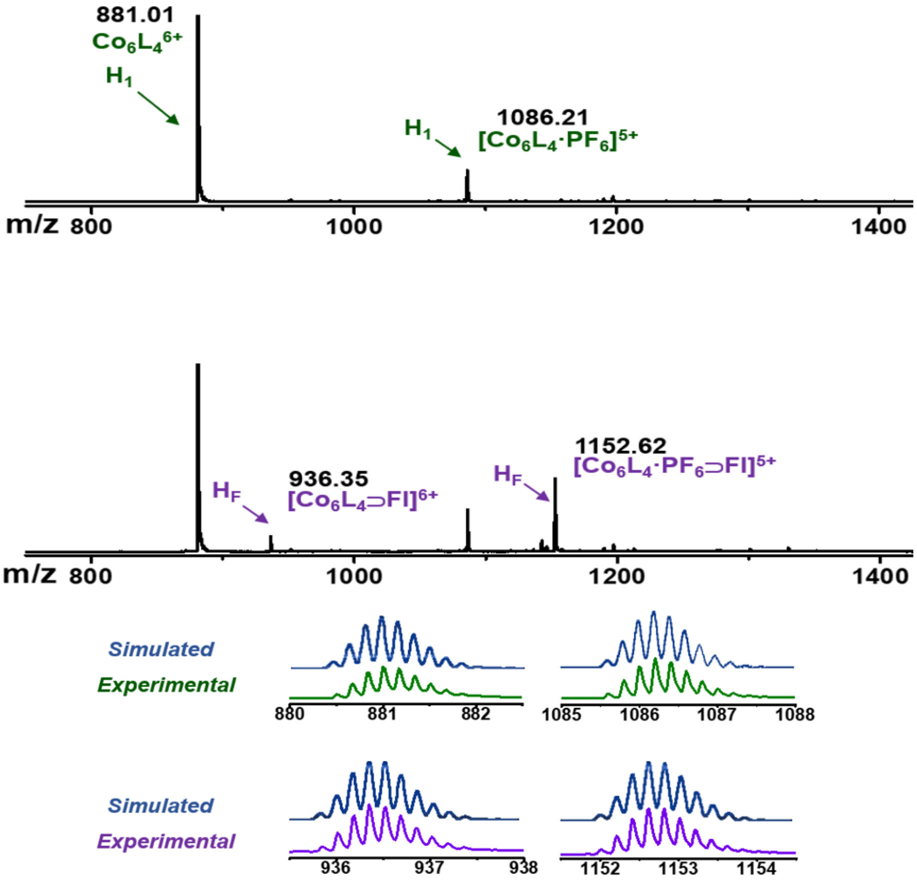

Peak	Value of m/z	Specie assigned
1	881.0157	$(Co_6L_4)^{6+}$
2	1086.2099	$[(Co_6L_4) \cdot PF_6]^{5+}$
3	936.3547	$[(Co_6L_4) \supset Fl]^{6+}$
4	1152.6194	$[(Co_6L_4 \supset Fl) \cdot PF_6]^{5+}$

Supplementary Figure 4. ESI-MS spectra of H_1 in acetonitrile solution. The inserts show the measured and simulated isotopic patterns at m/z = 881.02 and 1086.21, respectively (top). ESI-MS spectra of H_1 in acetonitrile solution with addition of fluorescein (Fl) to form H_F. The inserts show the measured and simulated isotopic patterns at m/z = 936.35 and 1152.62 (bottom).

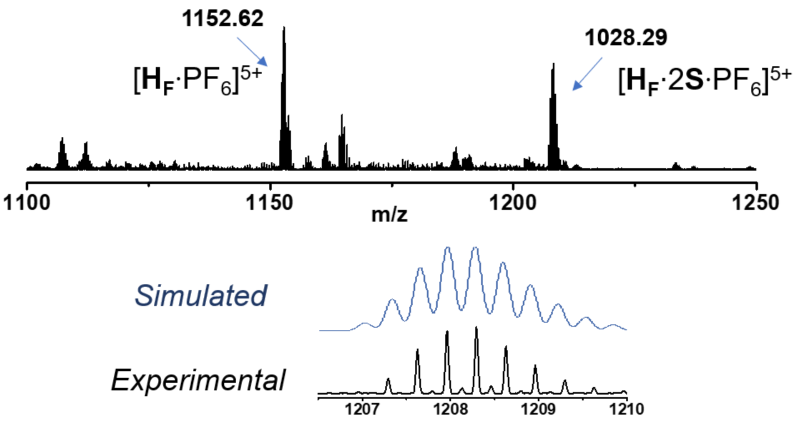

Supplementary Figure 5. ESI-MS spectra of H_1 in acetonitrile solution with addition of fluorescein (Fl) and substrate 4-hydroxyl-nitrobenzene (**S**) to form $H_F \supset$ 2S species. The inserts show the measured and simulated isotopic patterns at m/z = 1028.2943.

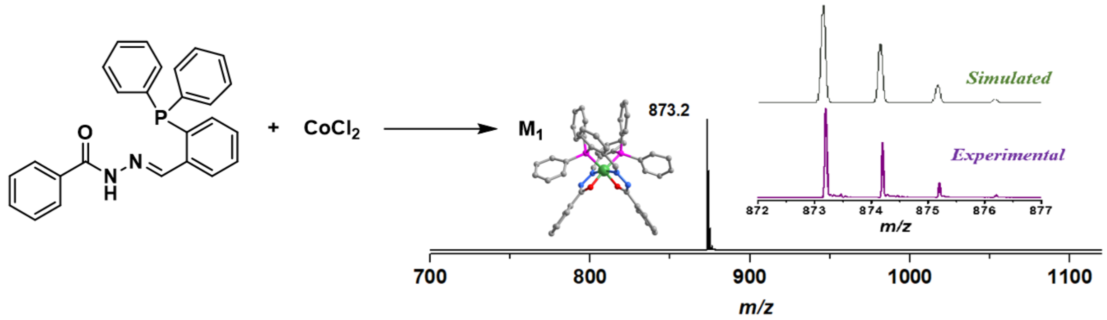

Supplementary Figure 6. ESI-MS spectra of M_1 in acetonitrile solution.

6. The cyclic voltammetry experiment

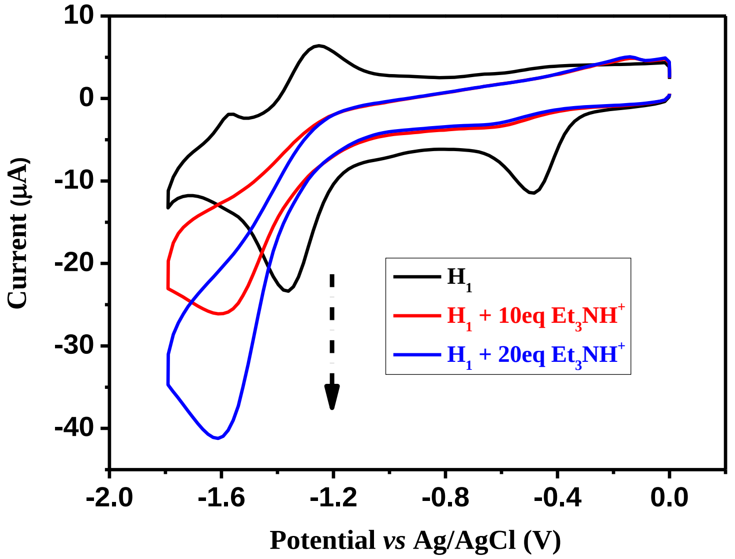

Supplementary Figure 7. Cyclic voltammogram of 0.1 mM H_1 and with addition of Et_3NH^+ in acetonitrile containing 0.1 M $TBAPF_6$, showing the capability for proton reduction under electrochemical condition. Scan Rate: 100 mV/s.

7. Fluorescence titration experiments data

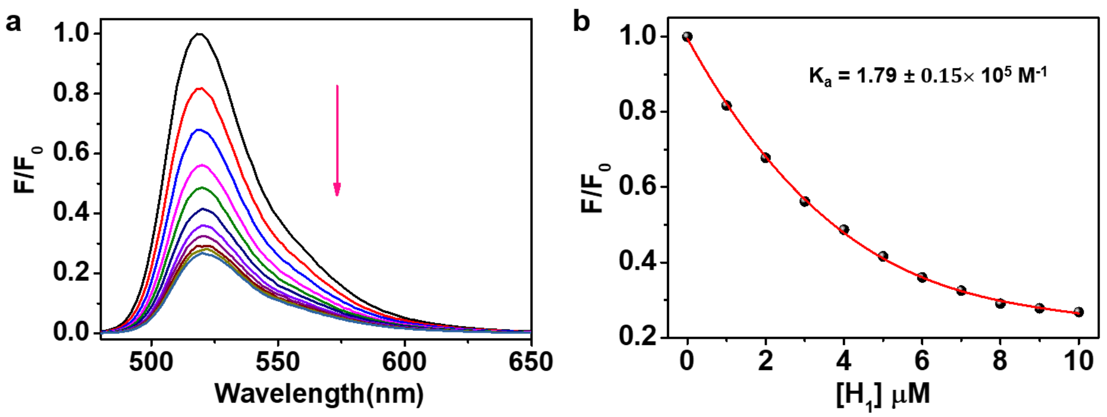

Supplementary Figure 8. (a) Emission spectra of Fl upon addition of H_1 (excited at 475 nm), and (b) the nonlinearity fitting of the titration curve. Fluorescence intensity was recorded at 520 nm and excited at 475 nm.

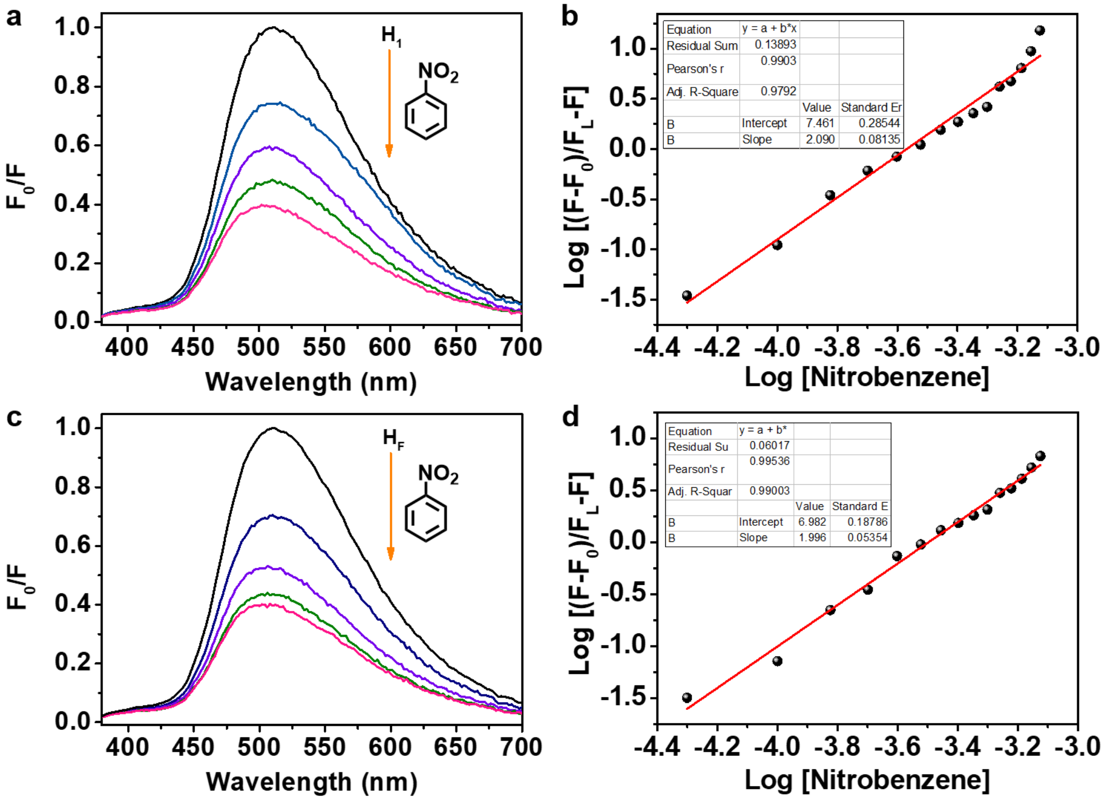

Supplementary Figure 9. (a) Emission spectra of H_1 (20.0 μM) upon addition of nitrobenzene in CH_3CN solution, the fluorescence intensity was recorded at 510 nm and excited at 365 nm. (b) The Hill-plot fitting of the titration curve showing a 1:2 host-guest behavior between H_1 and substrate nitrobenzene with an associated constant calculated as $2.89 \pm 0.43 \times 10^7$ M^{-2}. (c) Emission spectra of H_F (20.0 μM) upon addition of nitrobenzene in CH_3CN solution, the fluorescence intensity was recorded at 510 nm and excited at 365 nm. (d) The Hill-plot fitting of the titration curve showing a 1:2 host-guest behavior between H_1 and substrate nitrobenzene with a associated constant calculated as $9.78 \pm 0.48 \times 10^6$ M^{-2}.

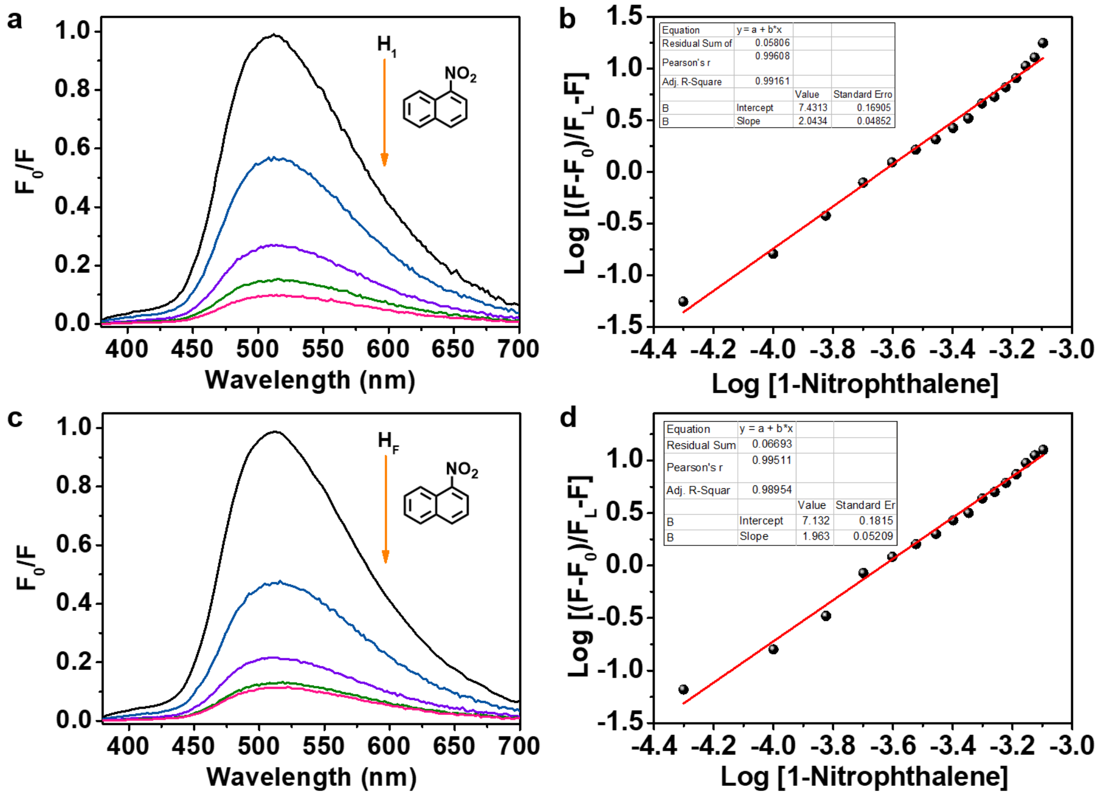

Supplementary Figure 10. (a) Emission spectra of H_1 (20.0 μM) upon addition of 1-nitronaphthalene in CH_3CN solution, the fluorescence intensity was recorded at 510 nm and excited at 365 nm. (b) The Hill-plot fitting of the titration curve showing a 1:2 host-guest behavior between H_1 and substrate 1-nitronaphthalene with an associated constant calculated as $2.70 \pm 0.13 \times 10^7$ M^{-2}. (c) Emission spectra of H_F (20.0 μM) upon addition of 1-nitronaphthalene in CH_3CN solution, the fluorescence intensity was recorded at 510 nm and excited at 365 nm. (d) The Hill-plot fitting of the titration curve showing a 1:2 host-guest behavior between H_1 and substrate 1-nitronaphthalene with an associated constant calculated as $1.36 \pm 0.07 \times 10^7$ M^{-2}.

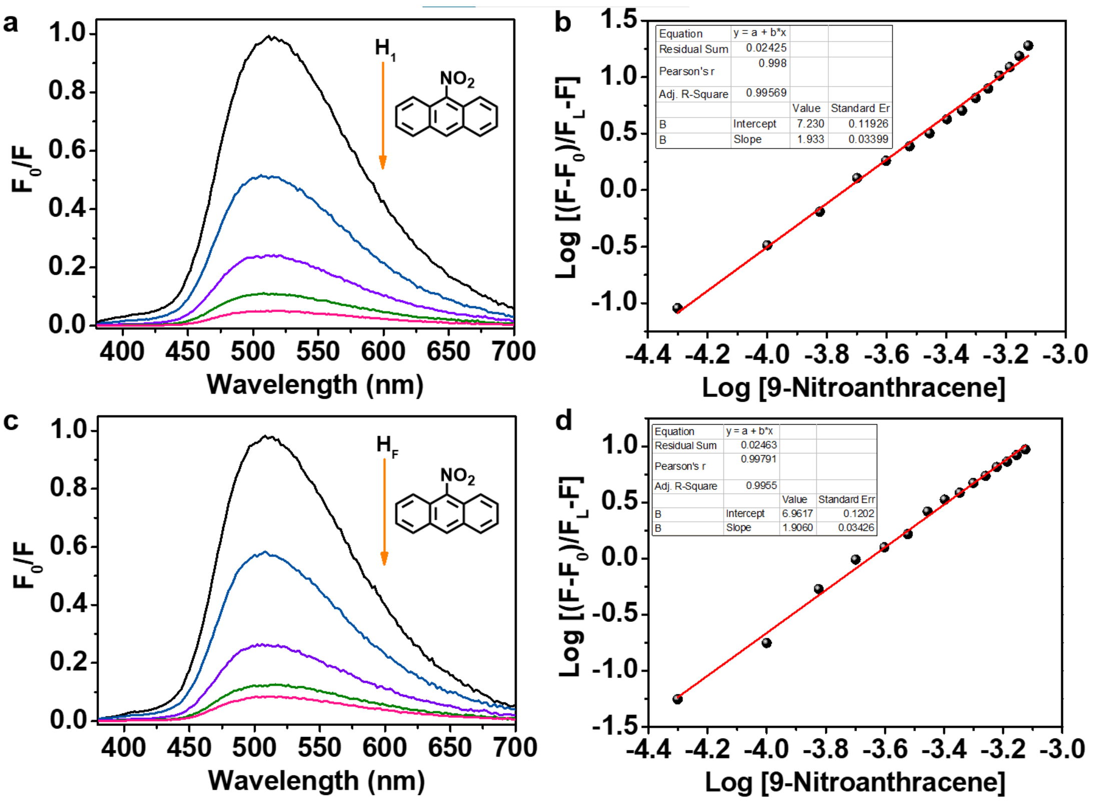

Supplementary Figure 11. (a) Emission spectra of H_1 (20.0 μM) upon addition of 9-nitroanthracene in CH_3CN solution, the fluorescence intensity was recorded at 510 nm and excited at 365 nm. (b) The Hill-plot fitting of the titration curve showing a 1:2 host-guest behavior between H_1 and substrate 9-nitroanthracene with an associated constant calculated as $1.69 \pm 0.05 \times 10^7$ M^{-2}. (c) Emission spectra of H_F (20.0 μM) upon addition of 1-nitroanthracene in CH_3CN solution, the fluorescence intensity was recorded at 510 nm and excited at 365 nm. (d) The Hill-plot fitting of the titration curve showing a 1:2 host-guest behavior between H_1 and substrate 9-nitroanthracene with an associated constant calculated as $9.12 \pm 0.23 \times 10^6$ M^{-2}.

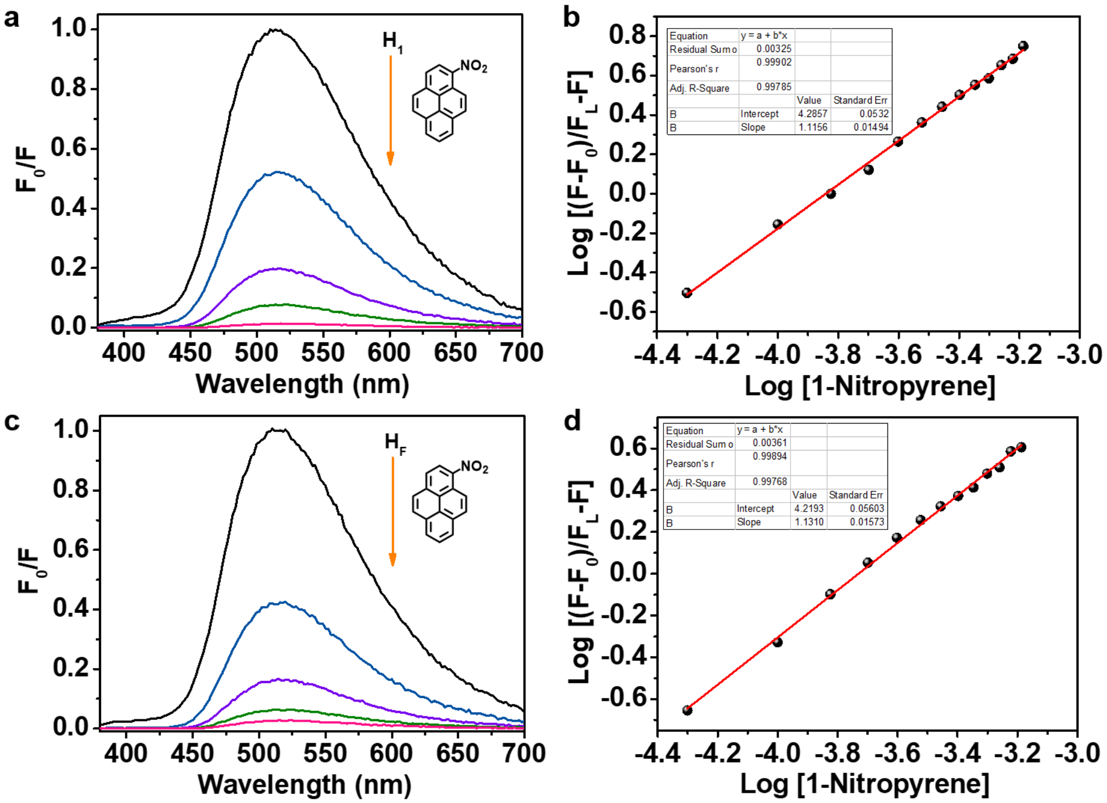

Supplementary Figure 12. (a) Emission spectra of H_1 (20.0 μM) upon addition of 1-nitropyrene in CH_3CN solution, the fluorescence intensity was recorded at 510 nm and excited at 365 nm. (b) The Hill-plot fitting of the titration curve showing a 1:1 host-guest behavior between H_1 and substrate 1-nitropyrene with an associated constant calculated as $1.93 \pm 0.01 \times 10^4$ M^{-1}. (c) Emission spectra of H_F (20.0 μM) upon addition of 1-nitropyrene in CH_3CN solution, the fluorescence intensity was recorded at 510 nm and excited at 365 nm. (d) The Hill-plot fitting of the titration curve showing a 1:1 host-guest behavior between H_1 and substrate 1-nitropyrene with an associated constant calculated as $1.66 \pm 0.01 \times 10^4$ M^{-1}.

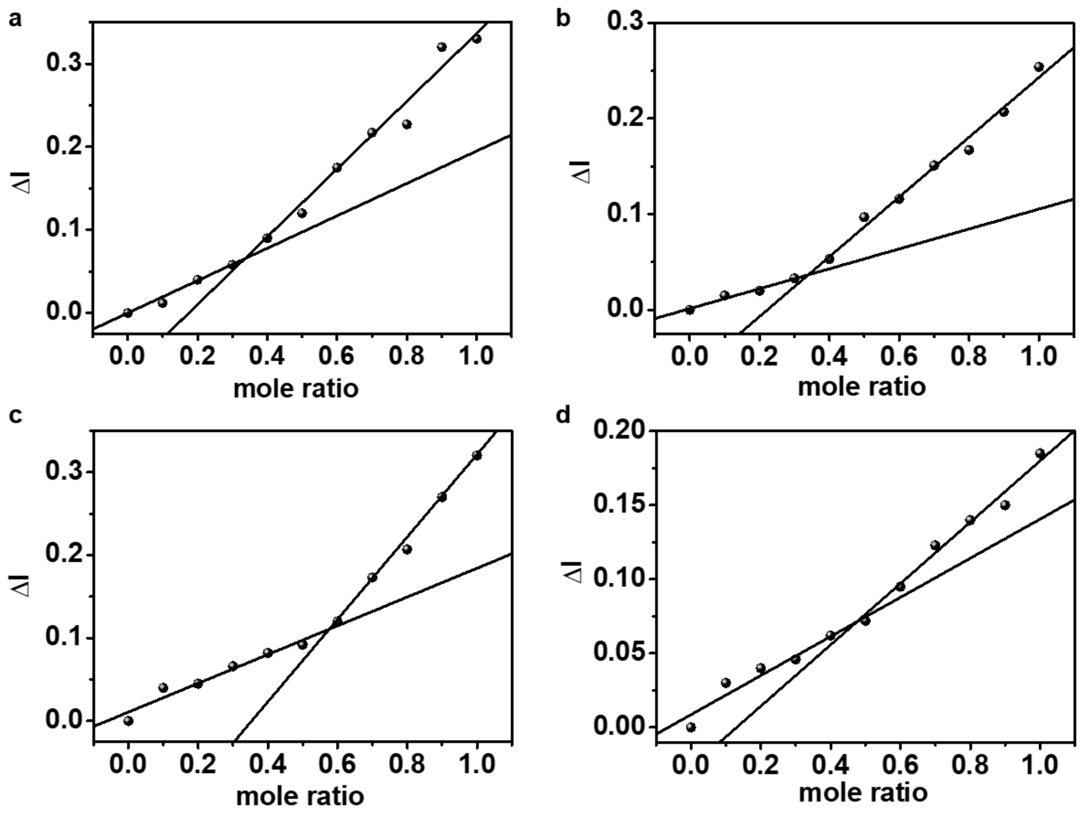

Supplementary Figure 13. (a) Job's plot for the 1:2 host-guest system of H_I⊃9-nitroanthracene in CH_3CN solution. (b) Job's plot for the 1:2 host-guest system of H_F⊃9-nitroanthracene in CH_3CN solution. (c) Job's plot for the 1:1 host-guest system of H_I⊃1-nitropyrene in CH_3CN solution. (d) Job's plot for the 1:1 host-guest system of H_F⊃1-nitropyrene in CH_3CN solution. Changes of the 510 nm fluorescence emission were monitored as a function of host-guest molar ratios (0:10 to 10:0).

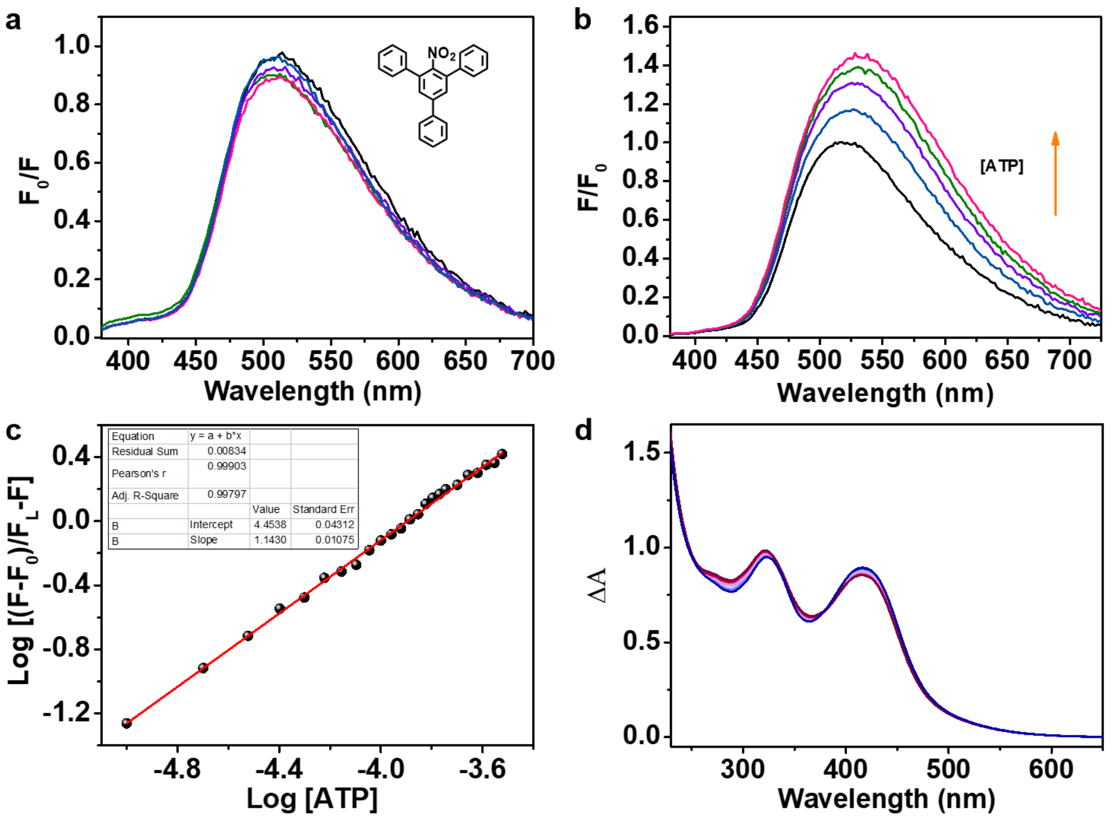

Supplementary Figure 14. (a) Emission spectra of H_1 (20.0 μM) upon addition of 2,4,6-triphenylnitrobenzene in CH_3CN solution, the fluorescence intensity was recorded at 510 nm and excited at 365 nm. (b) Emission spectra of H_1 (20.0 μM) upon addition of ATP in CH_3CN solution, showing the emission enhancement of H_1 excited at 365 nm. (c) The Hill-plot fitting of the titration curve showing a 1:1 host-guest behavior between H_1 and ATP with an associated constant calculated as $2.88 \pm 0.04 \times 10^4$ M^{-1}. (d) UV/Vis spectra of H_1 (10.0 μM) upon addition of ATP in CH_3CN solution. The presence of sharp isosbestic point at 380 nm indicated the host-guest behavior between H_1 and ATP.

8. NMR spectra data for host-guest behavior and diffusion coefficients.

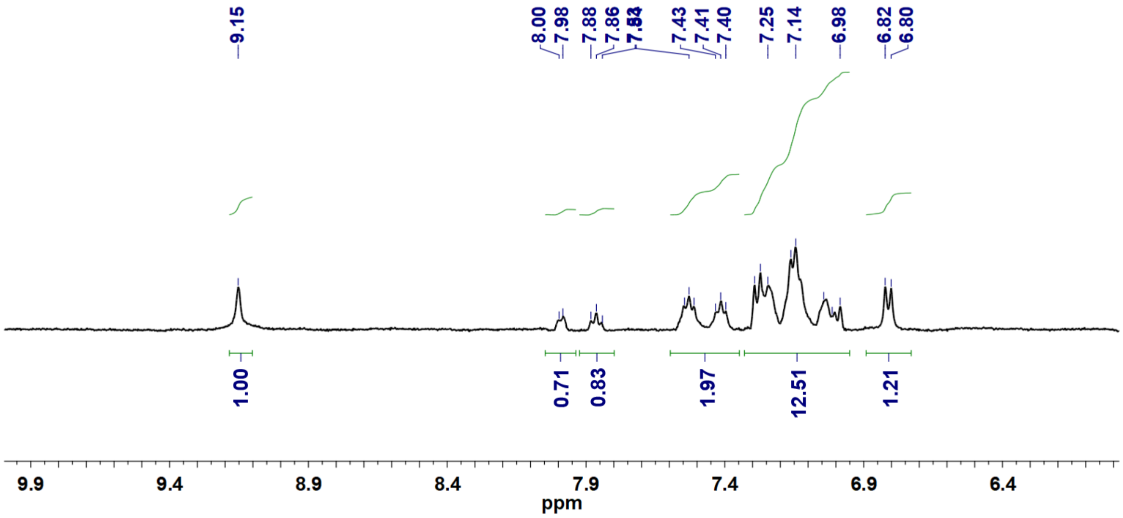

Supplementary Figure 15. ^{1}H NMR spectra (400 MHz, 298 K, CD_3CN) of 1.0 mM H_1.

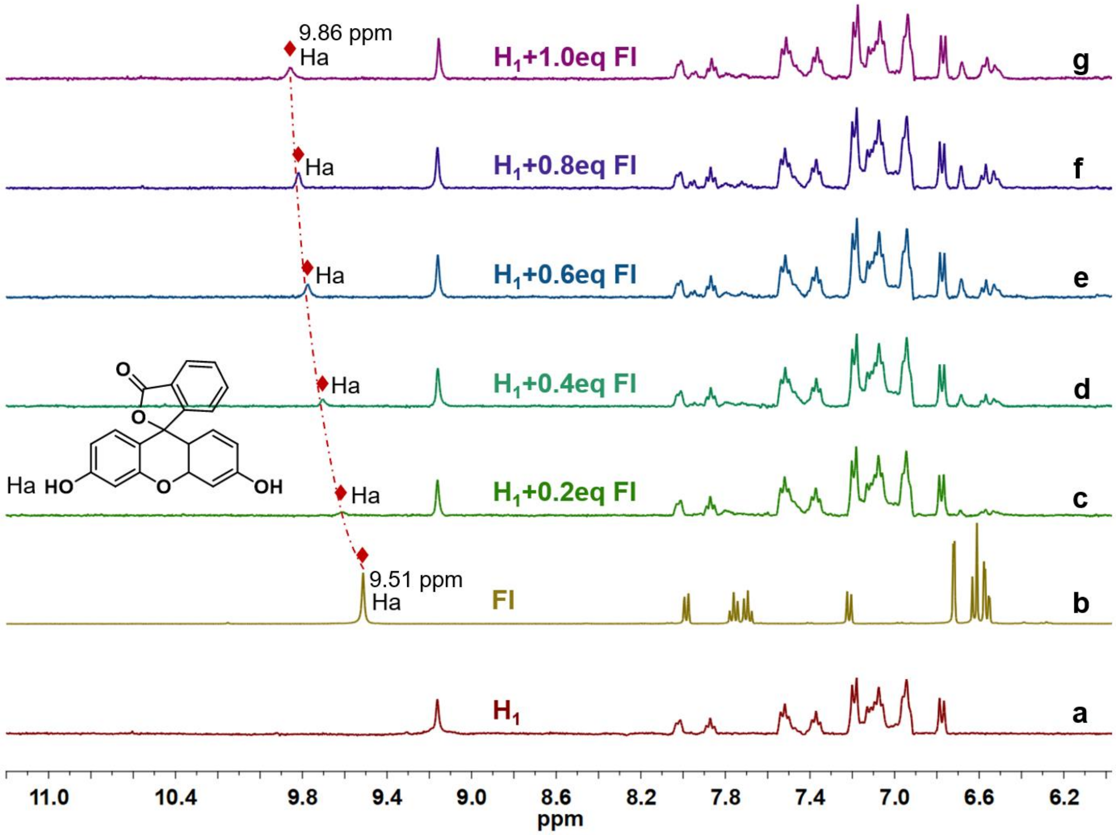

Supplementary Figure 16. ^{1}H NMR titration experiment between H_1 and Fl was carried out by adding Fl successively into a 1.0 mM H_1 solution at 273 K, showing the interactions between H_1 and Fl. (a) 1.0 mM H_1, (b) 10.0 mM Fl, (c) 1.0 mM H_1 and 0.2 mM Fl, (d) 1.0 mM H_1 and 0.4 mM Fl, (e) 1.0 mM H_1 and 0.6 mM Fl, (f) 1.0 mM H_1 and 0.8 mM Fl, (g) 1.0 mM H_1 and 1.0 mM Fl.

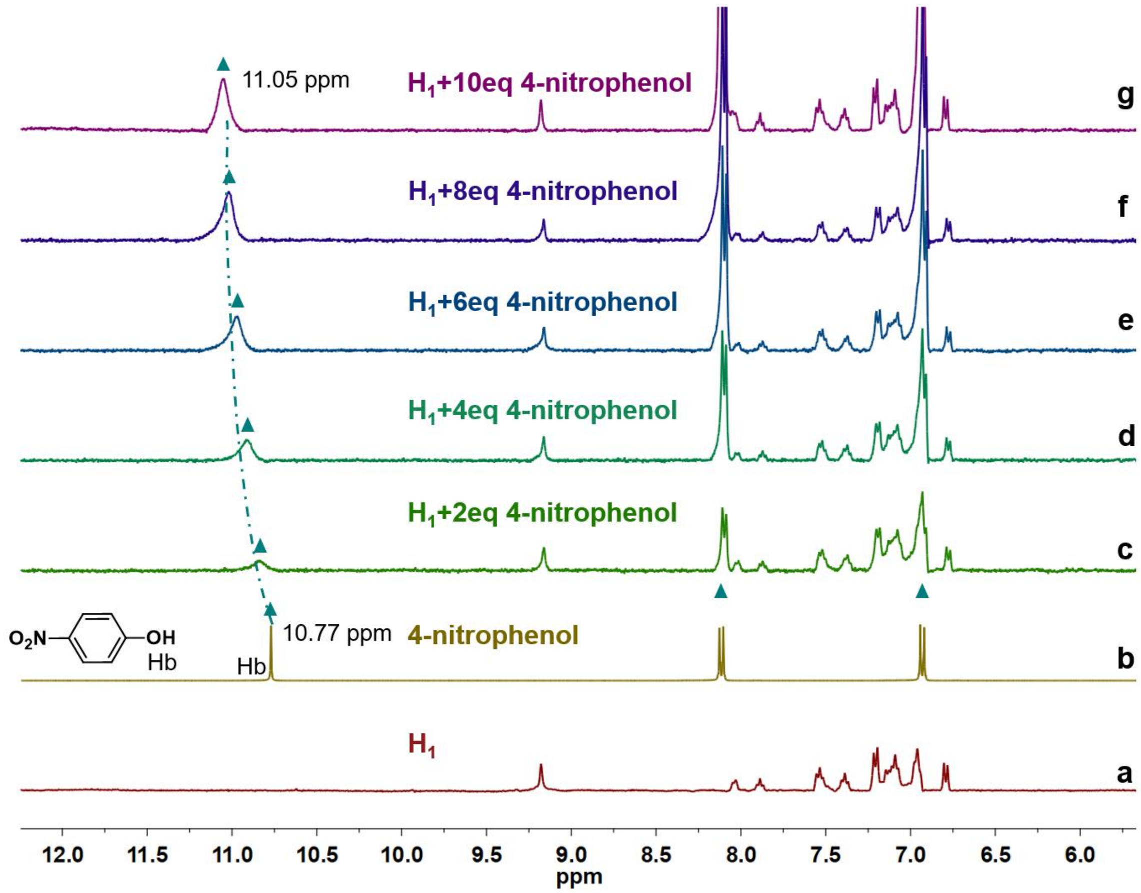

Supplementary Figure 17. ^{1}H NMR titration experiment between H_1 and 4-nitrophenol was carried out by adding 4-nitrophenol successively into a 1.0 mM H_1 solution at 273 K, showing the interactions between H_1 and 4-nitrophenol. (a) 1.0 mM H_1, (b) 0.1 M 4-nitrophenol, (c) 1.0 mM H_1 and 2.0 mM 4-nitrophenol, (d) 1.0 mM H_1 and 4.0 mM 4-nitrophenol, (e) 1.0 mM H_1 and 6.0 mM 4-nitrophenol, (f) 1.0 mM H_1 and 8.0 mM 4-nitrophenol, (g) 1.0 mM H_1 and 10.0 mM 4-nitrophenol.

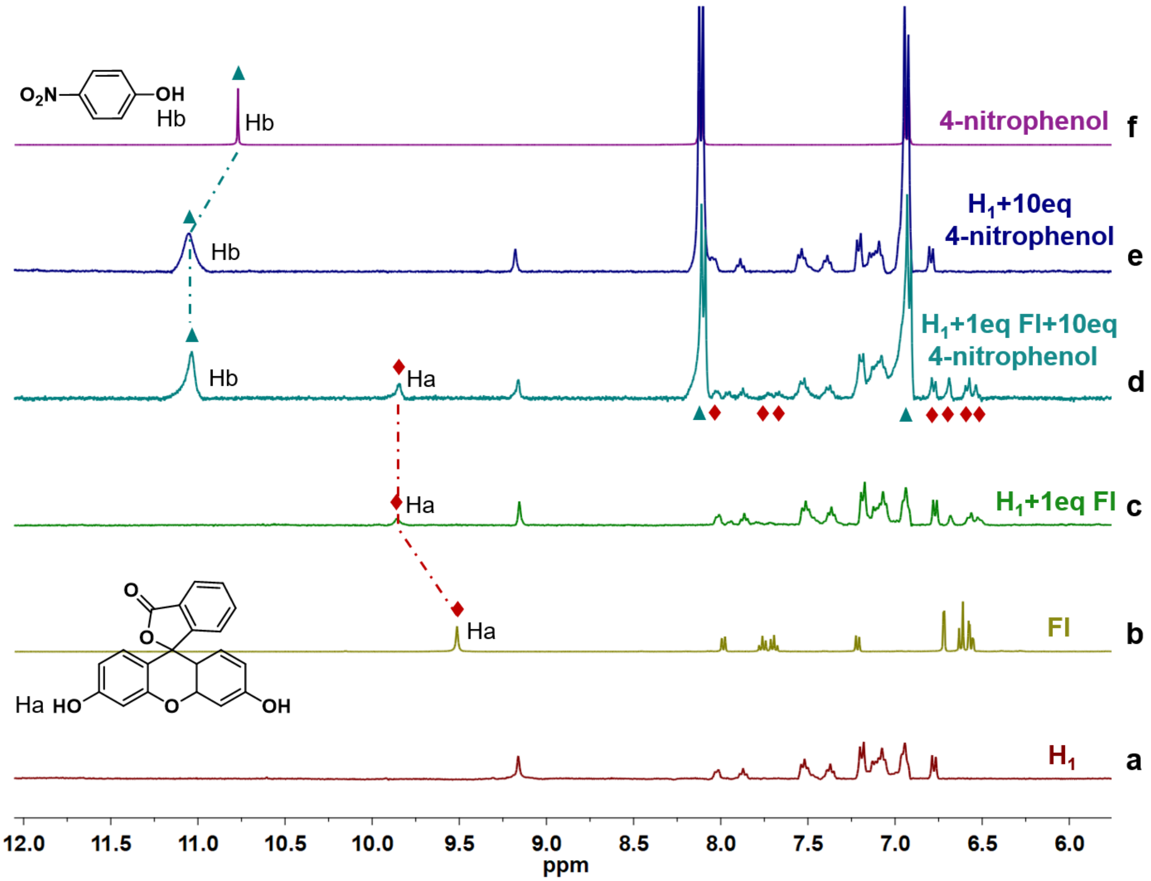

Supplementary Figure 18. ^{1}H NMR spectra of the mixture of H_1 (1.0 mM), Fl (1.0 mM), and 4-nitrophenol (10.0 mM) that measured at 273 K, showing the interactions of H_1, Fl, and 4-nitrophenol. (a) 1.0 mM H_1, (b) 10.0 mM Fl, (c) 1.0 mM H_1 and 1.0 mM Fl, (d) 1.0 mM H_1, 1.0 mM Fl and 10.0 mM 4-nitrophenol, (e) 1.0 mM H_1 and 10.0 mM 4-nitrophenol, (f) 0.1 M 4-nitrophenol.

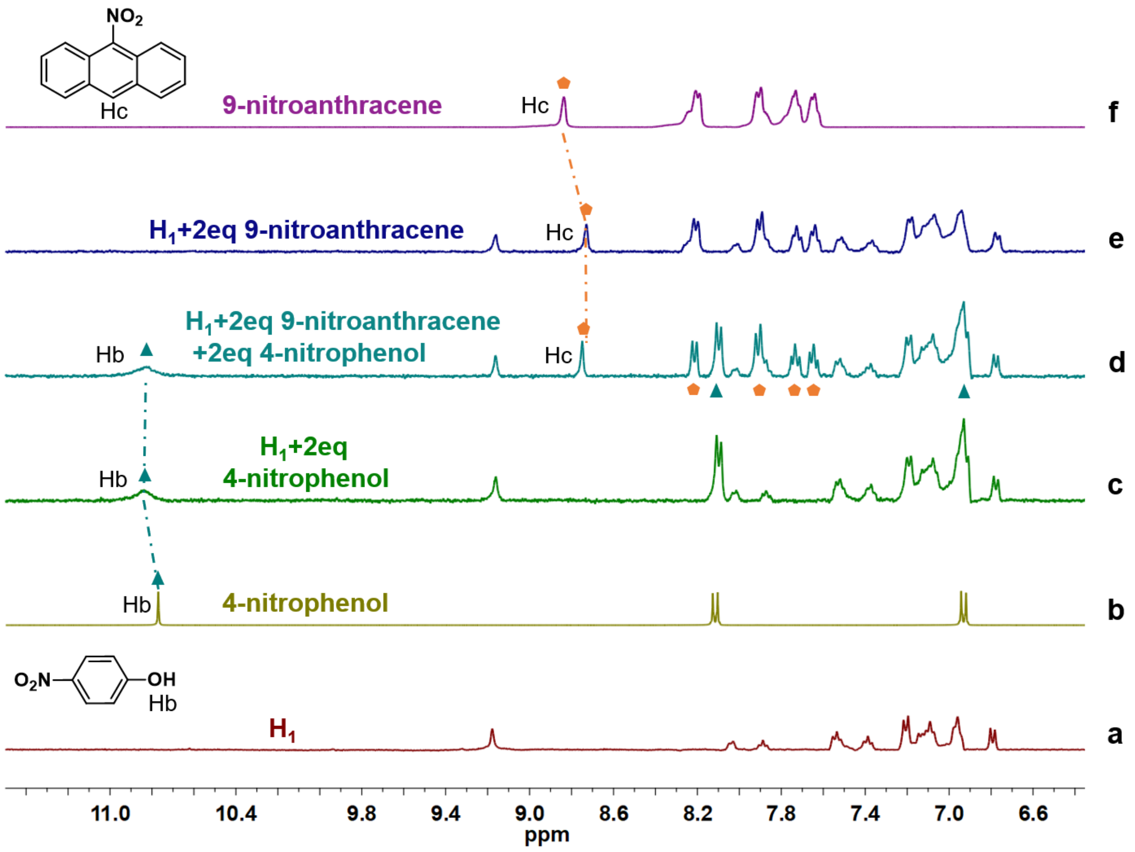

Supplementary Figure 19. ^{1}H NMR spectra of the mixture of H_1 (1.0 mM), 9-nitroanthracene (2.0 mM), and 4-nitrophenol (2.0 mM) that measured at 273 K, showing the interactions of H_1, 9-nitroanthracene, and 4-nitrophenol. (a) 1.0 mM H_1, (b) 0.1 M 4-nitrophenol, (c) 1.0 mM H_1 and 2.0 mM 4-nitrophenol, (d) 1.0 mM H_1, 2.0 mM 4-nitrophenol and 2.0 mM 9-nitroanthracene, (e) 1.0 mM H_1 and 2.0 mM 9-nitroanthracene, (f) 0.1 M 9-nitroanthracene.

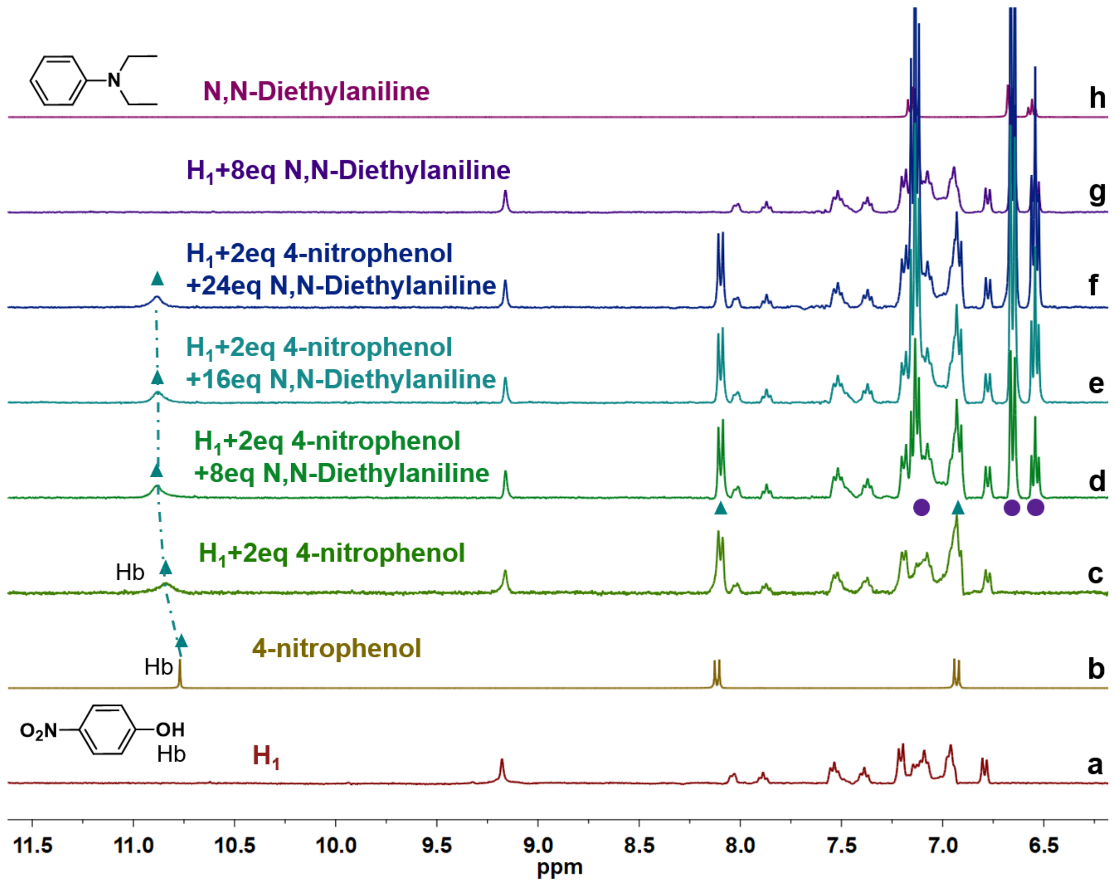

Supplementary Figure 20. ^{1}H NMR titration experiment of the mixture of 1.0 mM H_1 and 2.0 mM 4-nitrophenol with addition of N,N-diethylaniline (up to24.0 mM, the same ratio of the reaction conditions) that carried out at 273 K. (a) 1.0 mM H_1, (b) 0.1 M 4-nitrophenol, (c) 1.0 mM H_1 and 2.0 mM 4-nitrophenol, (d) 1.0 mM H_1, 2.0 mM 4-nitrophenol and 8.0 mM N,N-diethylaniline, (e) 1.0 mM H_1, 2.0 mM 4-nitrophenol and 16.0 mM N,N-diethylaniline, (f) 1.0 mM H_1, 2.0 mM 4-nitrophenol and 24.0 mM N,N-diethylaniline, (g) 1.0 mM H_1 and 8.0 mM N,N-diethylaniline, (h) 1.0 M N,N-diethylaniline.

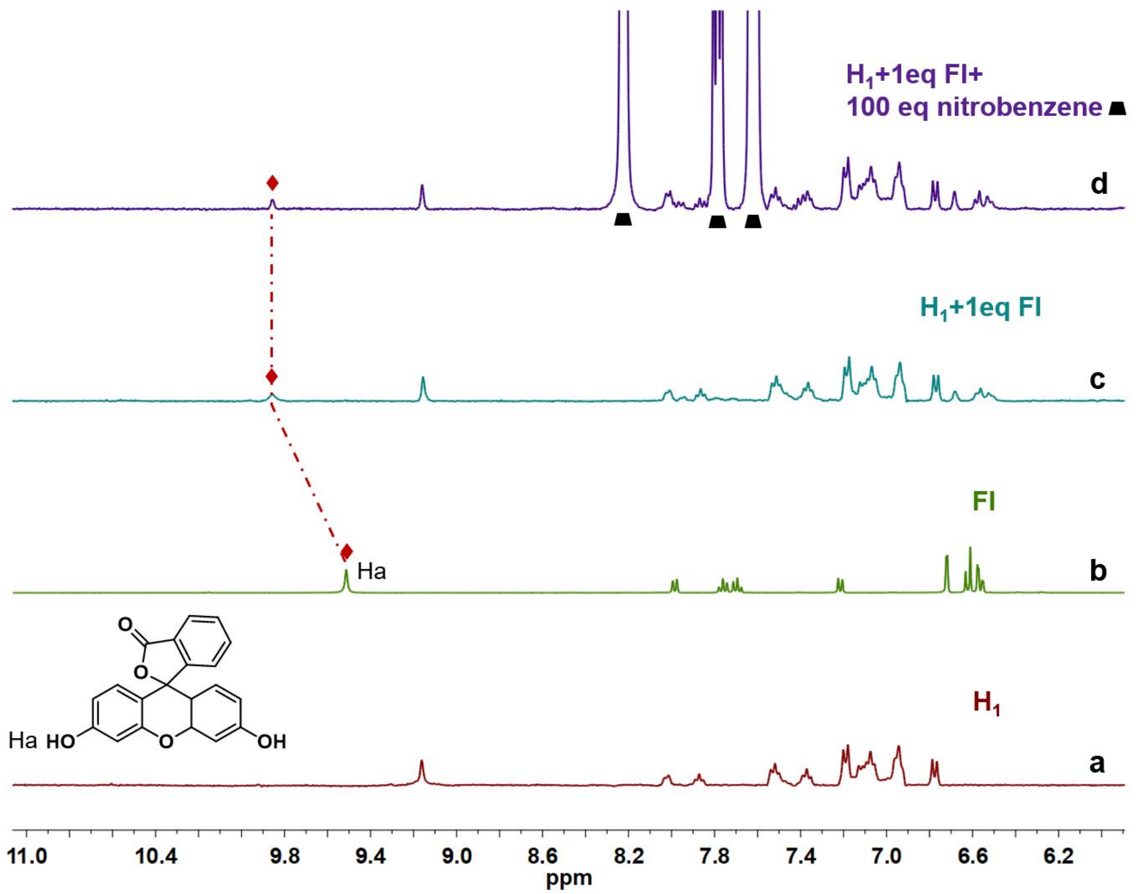

Supplementary Figure 21. ^{1}H NMR spectra of the mixture of H_1 (1.0 mM) and Fl (1.0 mM) with addition of 100.0 mM nitrobenzene that measured at 273 K. (a) 1.0 mM H_1, (b) 10.0 mM Fl, (c) 1.0 mM H_1 and 1.0 mM Fl, (d) 1.0 mM H_1, 1.0 mM Fl and 100.0 M nitrobenzene.

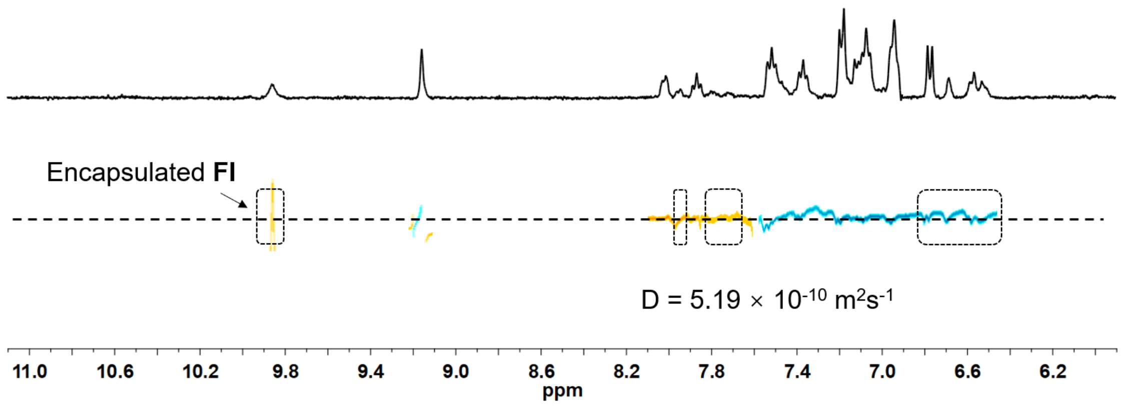

Supplementary Figure 22. ^{1}H DOSY spectra of the mixture containing H_1 (1.0 mM) and Fl (1.0 mM) in CD_3CN that measured at 273 K, showing that all signals of Fl and H_1 had the same diffusion coefficient $D = 5.19 \times 10^{-10}$ m^2s^{-1}. The yellow parts mean that the signals were weak.

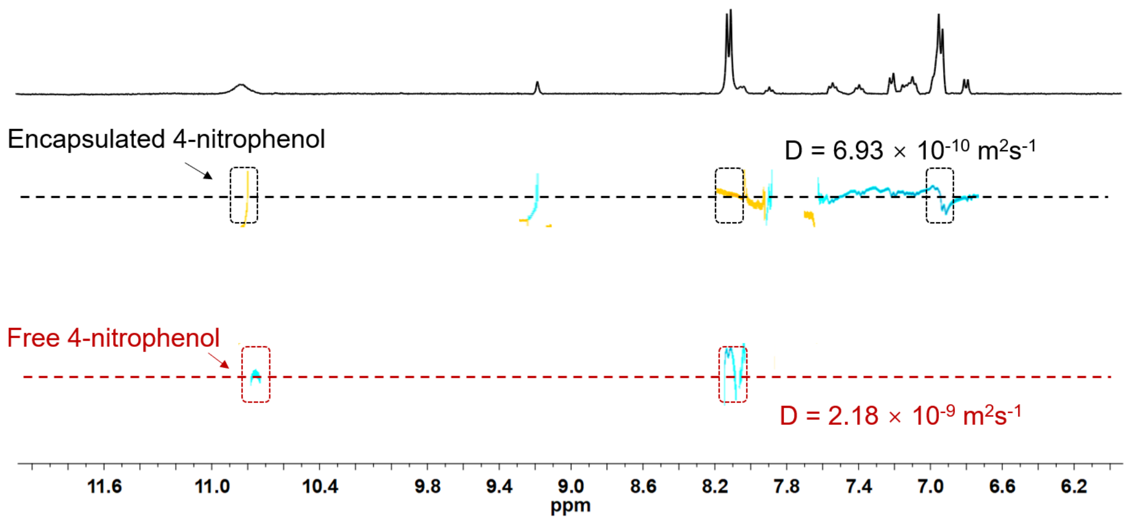

Supplementary Figure 23. ^{1}H DOSY spectra of the mixture containing H_1 (1.0 mM) and 4-nitrophenol (5.0 mM) in CD_3CN that measured at 273 K, showing the bound substrate ^{1}H signals exhibited the same diffusion coefficients as H_1 ($D = 6.93 \times 10^{-10}$ m^2s^{-1}), while the free substrates exhibited the diffusion coefficient of $D = 2.18 \times 10^{-9}$ m^2s^{-1}. The yellow parts mean that the signals were weak.

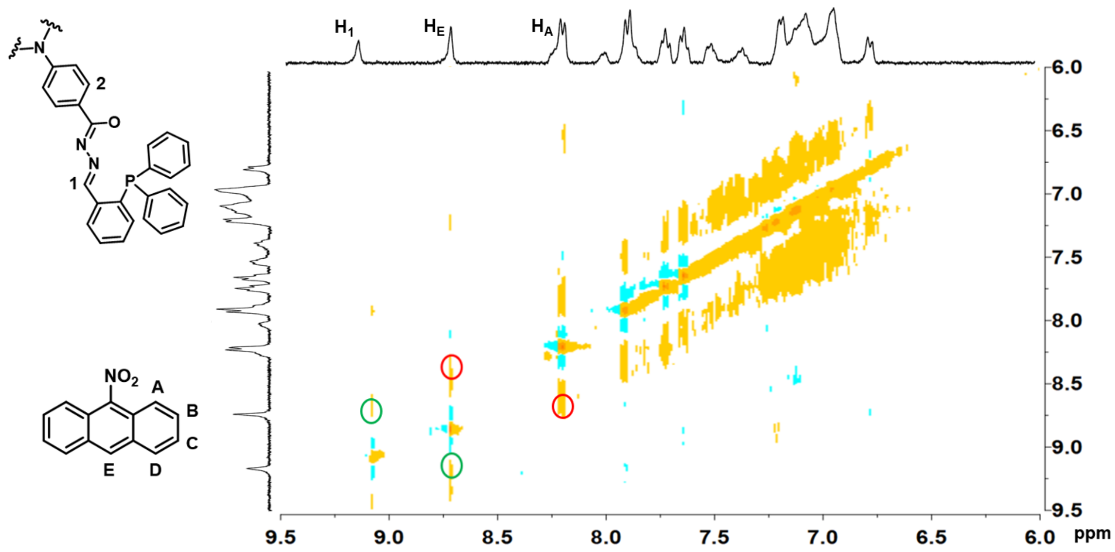

Supplementary Figure 24. The NOESY spectrum of a CD_3CN solution containing H_1 (0.1 mM) and 9-nitroanthracene (0.2 mM), showing obvious H−H interactions between the central phenyl ring of the 9-nitroanthracene and the CH=N fragment of H_1 (green circle) and between the phenyl rings from one substrate and the phenyl rings from the other substrate (red circle).

Supplementary Table 3. Summaries of the diffusion coefficients of the mentioned substrates and electron donors.

Substrates	Diff. Con. ($m^2s^{-1}\times10^{-9}$)	Electron donors	Diff. Con. ($m^2s^{-1}\times10^{-9}$)
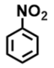	2.22	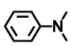	2.47
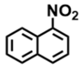	2.09	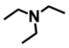	2.30
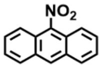	1.84	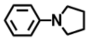	2.27
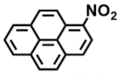	1.73	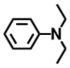	2.15
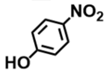	2.16	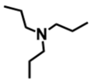	2.11
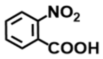	2.11		
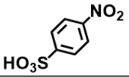	2.09		

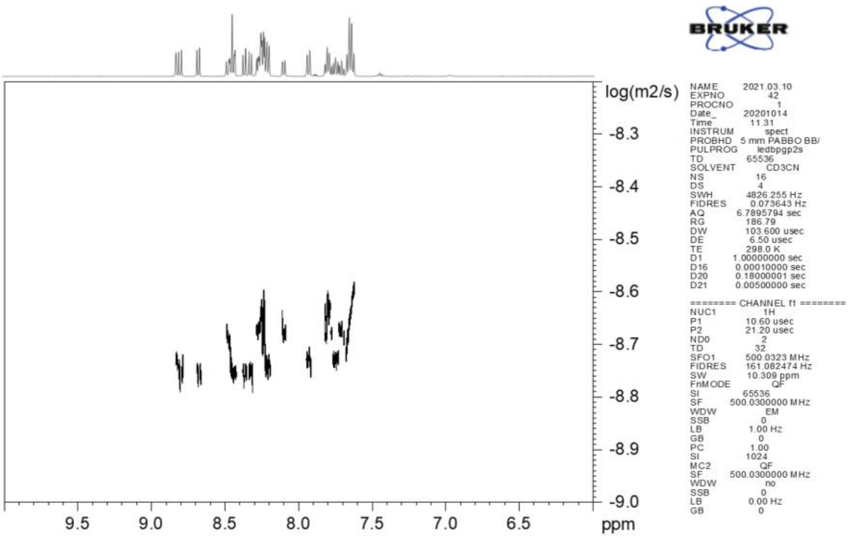

Supplementary Figure 25. ^1H DOSY NMR spectra data for different nitro substrates (nitrobenzene, 1-nitronaphthalene, 9-nitroanthracene, and 1-nitropyrene), showing the different diffusion coefficients.

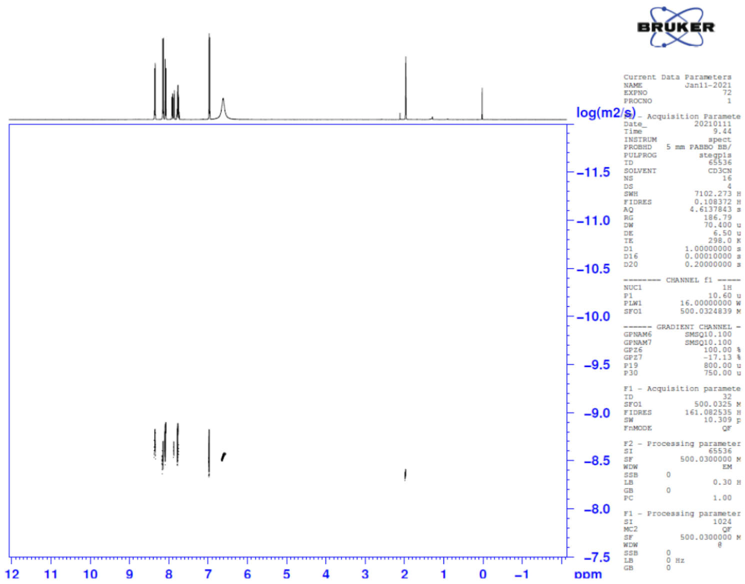

Supplementary Figure 26. ^1H DOSY NMR spectra data for different nitro substrates (4-hydroxynitrobenzene, 2-carboxynitrobenzene, and 4-sulfonicnitrobenzene), showing the different diffusion coefficients.

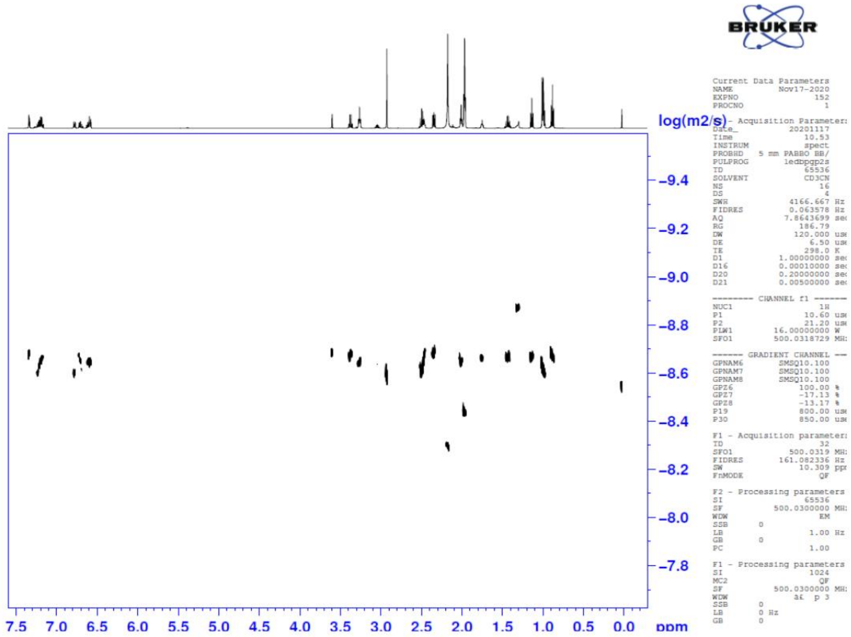

Supplementary Figure 27. ^1H DOSY NMR spectra data for different electron donors, showing the different diffusion coefficients.

9. The photocatalytic hydrogen generation using Na_2S as reductant.

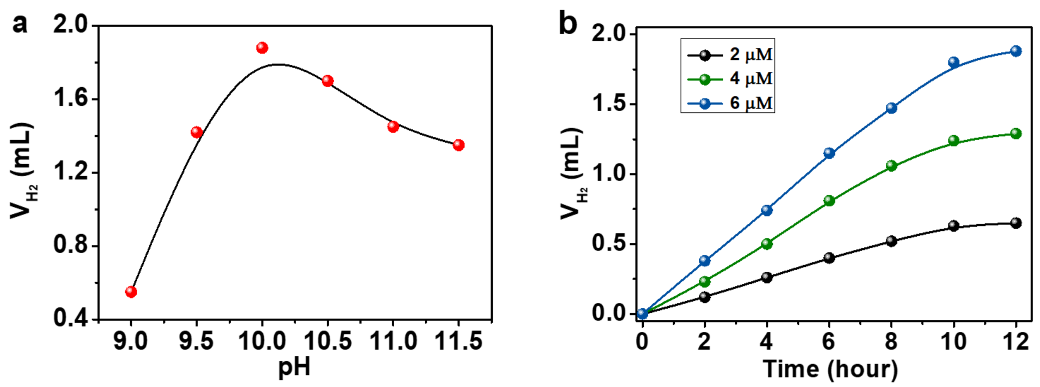

Supplementary Figure 28. The photocatalytic hydrogen generation in 4:1 acetonitrile/H_2O (v/v) solution containing H_1 (6.0 μM), Fl (1.0 mmol) and Na_2S (0.6 mmol) at different pH values, showing the optimal pH value for the photocatalytic system at 10.0. (b) The kinetics of the photocatalytic hydrogen generation for systems in 4:1 acetonitrile/H_2O (v/v) solution containing Fl (1.0 mM) and Na_2S (0.6 mmol) at pH = 10.0 with different concentration of H_1.

10. Control experiments of the photocatalytic reduction of nitrobenzene.

Supplementary Table 4. Control experiments of the photocatalytic reduction of nitrobenzene.

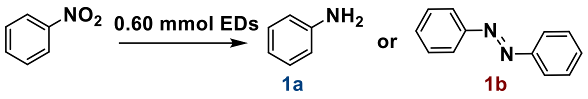

Entry	Catalyst	EDs	Yield (%)	
			Light	Dark
1	0.50 μmol **H_F**	Na_2S	1a (99%)	1a(<10%)
2	0.50 μmol **H_1**	Na_2S	1a (< 5%)	1a (<5%)
3	0.50 μmol **Fl**	Na_2S	1a(<10%)	1a (< 5%)
4[a]	3.0 μmol **M_1**	Na_2S	1a (< 5%)	1a (< 5%)
5[a]	3.0 μmol **M_1** + 0.50 μmol **Fl**	Na_2S	1a (32%)	1a(<10%)
6[b]	2.0 μmol H_3L + 3.0 μmol $CoCl_2$	Na_2S	1a (< 5%)	1a (< 5%)
7[b]	2.0 μmol H_3L + 3.0 μmol $CoCl_2$ + 0.50 μmol **Fl**	Na_2S	1a (11%)	1a (< 5%)
8[b]	3.0 μmol $CoCl_2$	Na_2S	1a (< 5%)	1a (< 5%)
9[b]	3.0 μmol $CoCl_2$ + 0.50 μmol **Fl**	Na_2S	1a(<10%)	1a (< 5%)
10	0.50 μmol **H_F**	tripropylamine	1b (99%)	—
11	0.50 μmol **H_1**	tripropylamine	—	—
12	0.50 μmol **Fl**	tripropylamine	—	—
13[a]	3.0 μmol **M_1**	tripropylamine	—	—
14[a]	3.0 μmol **M_1** + 0.50 μmol **Fl**	tripropylamine	1a (18%)	—
15[b]	2.0 μmol H_3L + 3.0 μmol $CoCl_2$	tripropylamine	—	—
16[b]	2.0 μmol H_3L + 3.0 μmol $CoCl_2$ + 0.50 μmol **Fl**	tripropylamine	1a (< 5%)	—
17[b]	3.0 μmol $CoCl_2$	tripropylamine	—	—
18[b]	3.0 μmol $CoCl_2$ + 0.50 μmol **Fl**	tripropylamine	1a (< 5%)	—

[a]The mononuclear M_1 was synthesized by refluxing cobalt salts and (2-(di-phenylphosphanyl)benzylidene)benzene carbohydrzide, which resembled a corner of H_1 and exhibited the identical cobalt-based redox property. [b] To guarantee the equal amount of cobalt ions, the amount of the addition of M_1 or cobalt salts in the control experiments was six times as much. The reaction time of Na_2S systems was 30 min, while the reaction time of tripropylamine systems was 6 h.

11. Kinetics experiments of photocatalytic of nitro substrates reduction using Na_2S as reductant.

During the reaction process, the tracking of the reaction process was carried by extraction of 50 μL reaction mixture every 5 minutes with a long needle and followed by HPLC analysis after the quick flush through the silica gel. The reaction rates in the first 5 minutes were regarded as the initial rate of the reactions.

Supplementary Table 5. The initial rate of different reduction of substrates (0.05 mmol), the selectivity formation of aminobenzene in the competing reactions with other different substrates respectively.

Substrates	Initial Rate ($\mu M \cdot s^{-1}$)	Selectivity (%)
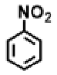	5.67	–
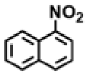	5.04	68
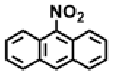	3.23	90
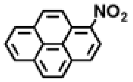	2.00	99

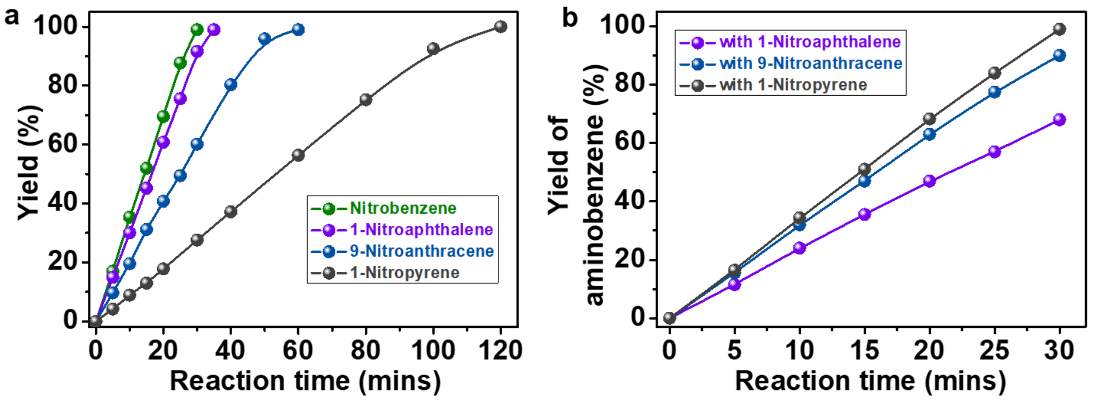

Supplementary Figure 29. (a) Kinetics experiments of reduction reactions of different nitro substrates (0.05 mmol) using Na_2S as reductant under the standard conditions. (b) Kinetics experiments of yields of aminobenzene in the competing reactions with the corresponding mixed substrates under the standard conditions.

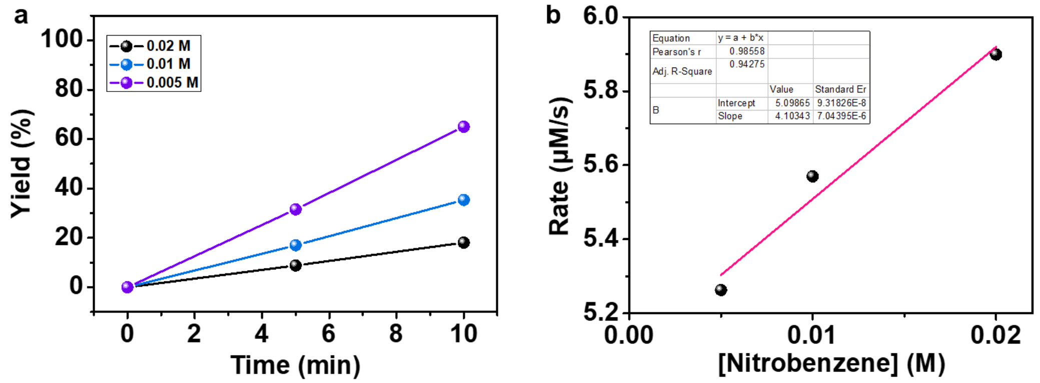

Supplementary Figure 30. (a) Kinetics experiments of reduction reactions of nitrobenzene using Na_2S as reductant with different concentration of nitrobenzene. (b) The relationship between the initial rate of nitrobenzene reduction and the concentration of nitrobenzene.

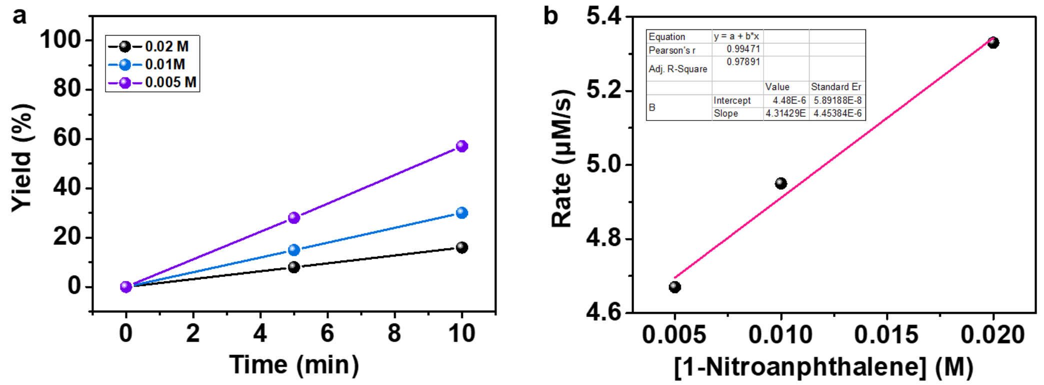

Supplementary Figure 31. (a) Kinetics experiments of reduction reactions of 1-nitroanphthalene using Na_2S as reductant with different concentration of 1-nitroanphthalene. (b) The relationship between the initial rate of 1-nitroanphthalene reduction and the concentration of 1-nitroanphthalene.

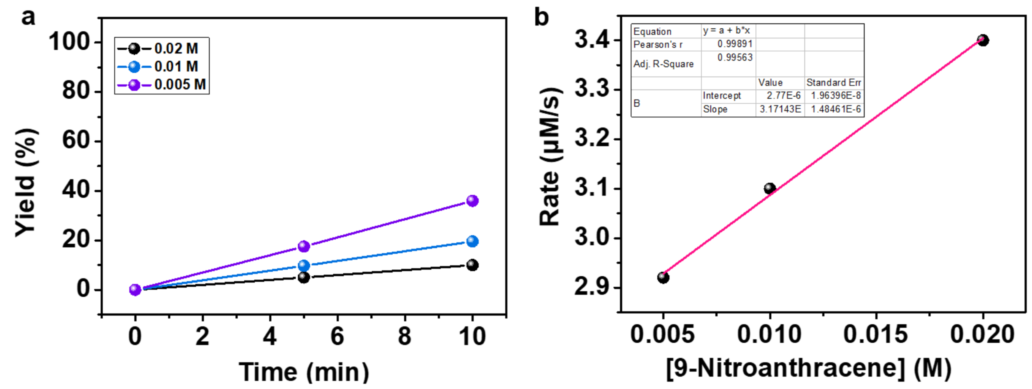

Supplementary Figure 32. (a) Kinetics experiments of reduction reactions of 9-nitroanthracene using Na_2S as reductant with different concentration of 9-nitroanthracene. (b) The relationship between the initial rate of 9-nitroanthracene reduction and the concentration of 9-nitroanthracene.

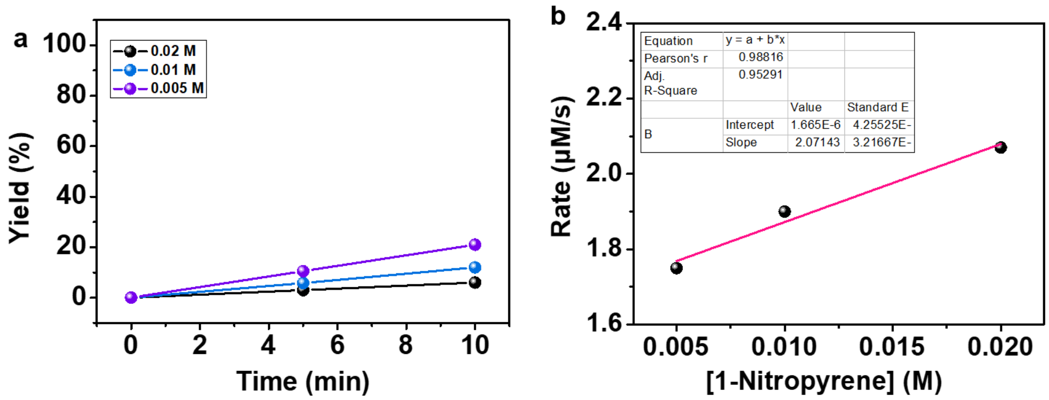

Supplementary Figure 33. (a) Kinetics experiments of reduction reactions of 1-nitropyrene using Na_2S as reductant with different concentration of 1-nitropyrene. (b) The relationship between the initial rate of 1-nitropyrene reduction and the concentration of 1-nitropyrene.

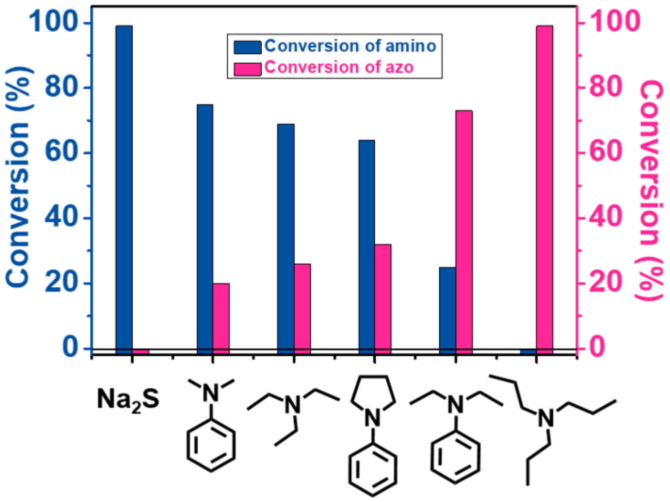

Supplementary Figure 34. Selective reduction of 9-nitroanthracene with different reductants, showing the conversions of amino and azo products, respectively.

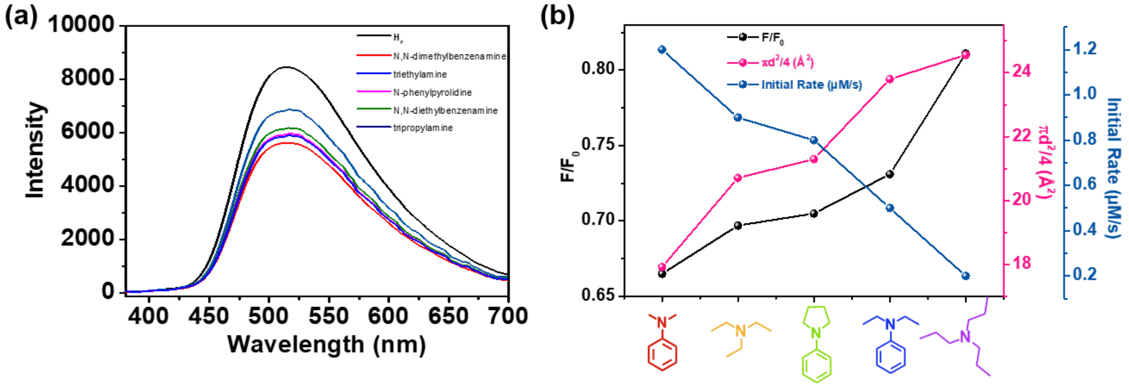

Supplementary Figure 35. (a) Emission spectra of H_F (20.0 μM) upon addition of varieties of electron donors with the same proportion according to the photocatalytic reaction. (b) The relationship of fluorescence quenching, the estimated diameter and the initial rate of 1-nitropyrene reduction according to different electron donors.

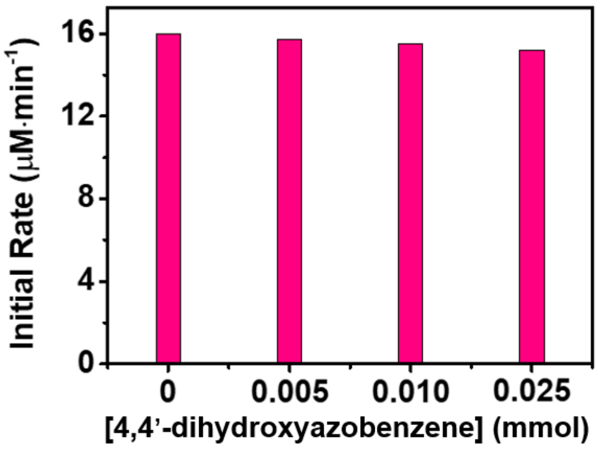

Supplementary Figure 36. The initial conversion rate of the azo product from 4-nitrophenol using tripropylamine as electron donor under standard conditions upon addition of 4,4'-dihydroxyazobenzene 0.005 mmol, 0.010 mmol, and 0.025 mmol respectively.

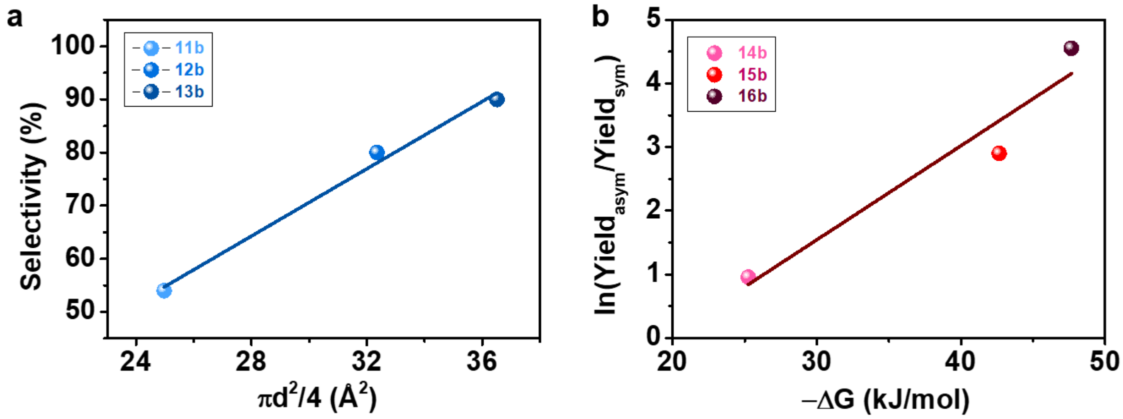

Supplementary Figure 37. (a) The relationship between the selectivity of asymmetric azo products (11b-13b) and estimated diameter of the partner substrates. (b) The relationship between the selectivity of the asymmetric azo products (14b-16b) and the inclusion free energy changes of the capsule and the partner substrates.

12. Microcalorimetric titration experiments of the interactions between catalyst and substrates.

The ITC was performed on a Nano ITC (TA Instruments Inc.–Waters LLC) at atmospheric parameters and at 25.0 ºC, giving the association constants and the thermodynamic parameters. The solution of guest in the syringe was sequentially injected under stirring at 250 rpm into a solution of host in the sample cell.

Supplementary Table 6. The thermodynamic data of observed by isothermal titration calorimetry of the different clathration species mentioned.

Clathration species	H_1+FI	H_F + 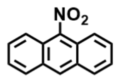	H_F+ 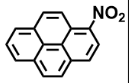	H_F + 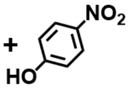	H_F + 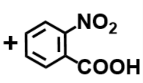
n	1.070	1.933	0.974	2.024	1.028
ΔH (kJ/mol)	-8.64	-0.60	-1.56	-2.41	-9.61
ΔS (J/mol)	83.03	95.17	111.54	76.68	110.87
ΔG (kJ/mol)	-33.38	-28.96	-34.80	-25.26	-42.65
Clathration species	H_F + 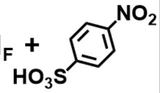	H_F + 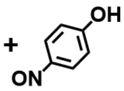	H_F + 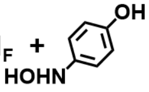	H_F + 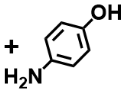	H_F + 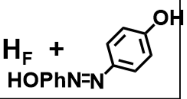
n	1.016	2.04	2.01	1.99	1.01
ΔH (kJ/mol)	-15.70	-1.05	-1.84	-1.18	-1.13
ΔS (J/mol)	107.25	76.71	94.19	76.91	77.65
ΔG (kJ/mol)	-47.66	-23.91	-29.91	-24.10	-24.27

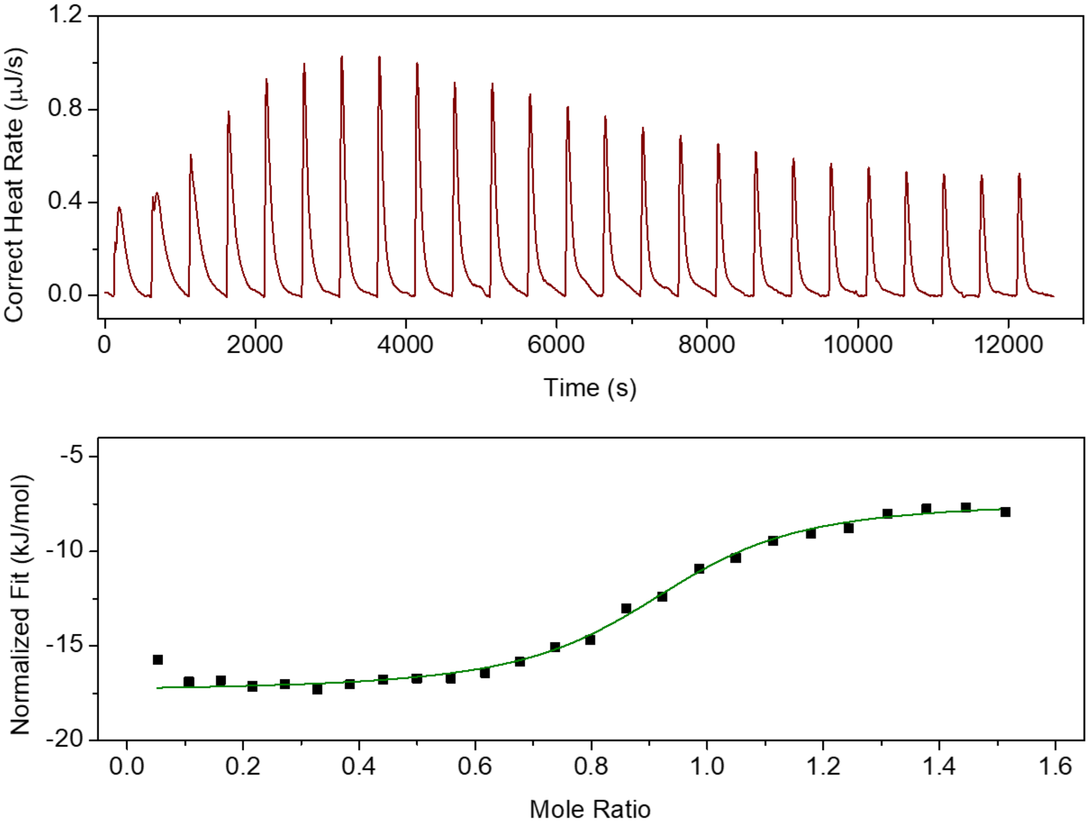

Model	Variable	Value	Confidence Interval (±)
	K_d (M)	1.570E-05	1.104E-06
	n	1.070	0.024
Independent	ΔH (kJ/mol)	-8.635	0.649
	ΔS (J/mol·K)	83.033	
	ΔG (kJ/mol)	-33.378	
	Confidence Level	99%	

Supplementary Figure 38. Microcalorimetric titration of H_1 in CH_3CN solution at 298.00 K. (Top) Raw data for sequential 25 injections (10 μL per injection) of Fl solution injecting into H_1 solution (0.1 mM). (Bottom) Apparent reaction heat obtained from the integration of calorimetric traces.

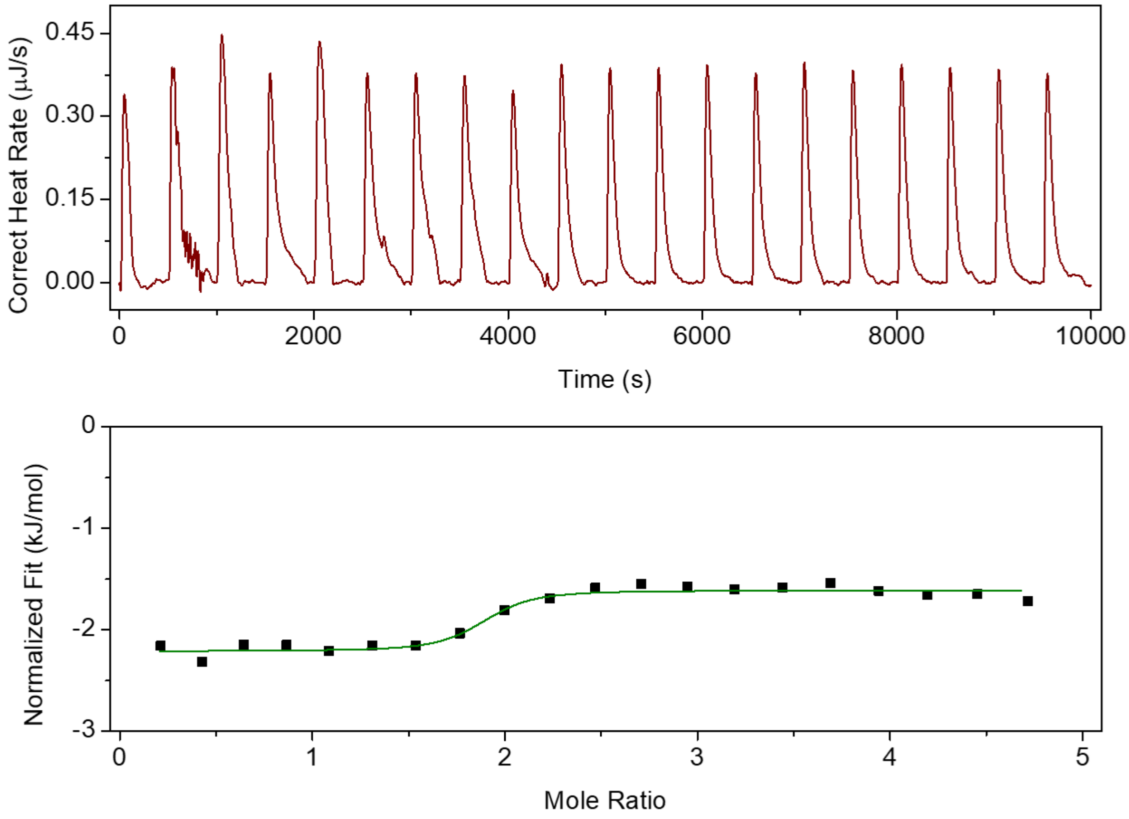

Model	Variable	Value	Confidence Interval (±)
	K_d (M)	7.561E-07	1.651E-08
	n	1.933	0.126
Independent	ΔH (kJ/mol)	-0.602	0.087
	ΔS (J/mol·K)	95.172	
	ΔG (kJ/mol)	-28.963	
	Confidence Level	99%	

Supplementary Figure 39. Microcalorimetric titration of H_F in CH_3CN solution at 298.00 K. (Top) Raw data for sequential 20 injections (10 μL per injection) of 9-nitroanthracene solution injecting into H_F solution (0.1 mM). (Bottom) Apparent reaction heat obtained from the integration of calorimetric traces.

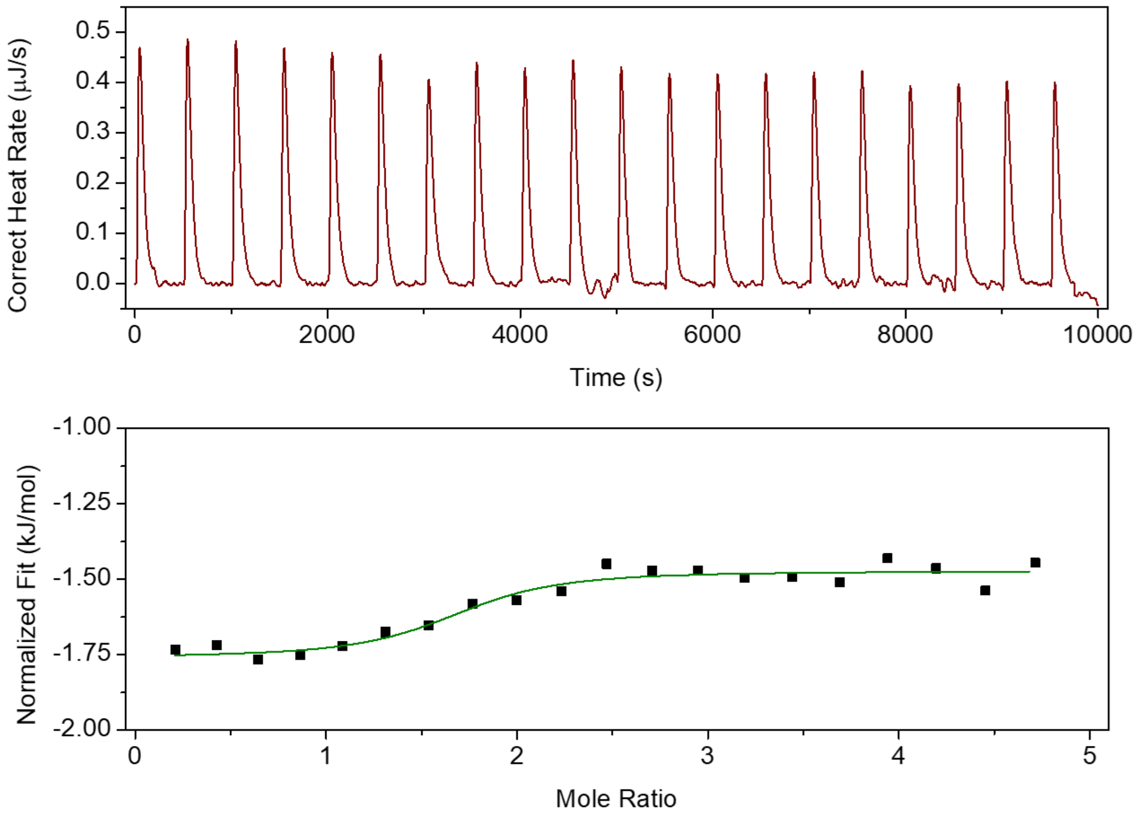

Model	Variable	Value	Confidence Interval (±)
	K_d (M)	2.309E-05	1.651E-06
	n	0.974	0.141
Independent	ΔH (kJ/mol)	-1.560	0.025
	ΔS (J/mol·K)	111.539	
	ΔG (kJ/mol)	-34.799	
	Confidence Level	99%	

Supplementary Figure 40. Microcalorimetric titration of H_F in CH_3CN solution at 298.00 K. (Top) Raw data for sequential 20 injections (10 μL per injection) of 1-nitropyrene solution injecting into H_F solution (0.1 mM). (Bottom) Apparent reaction heat obtained from the integration of calorimetric traces.

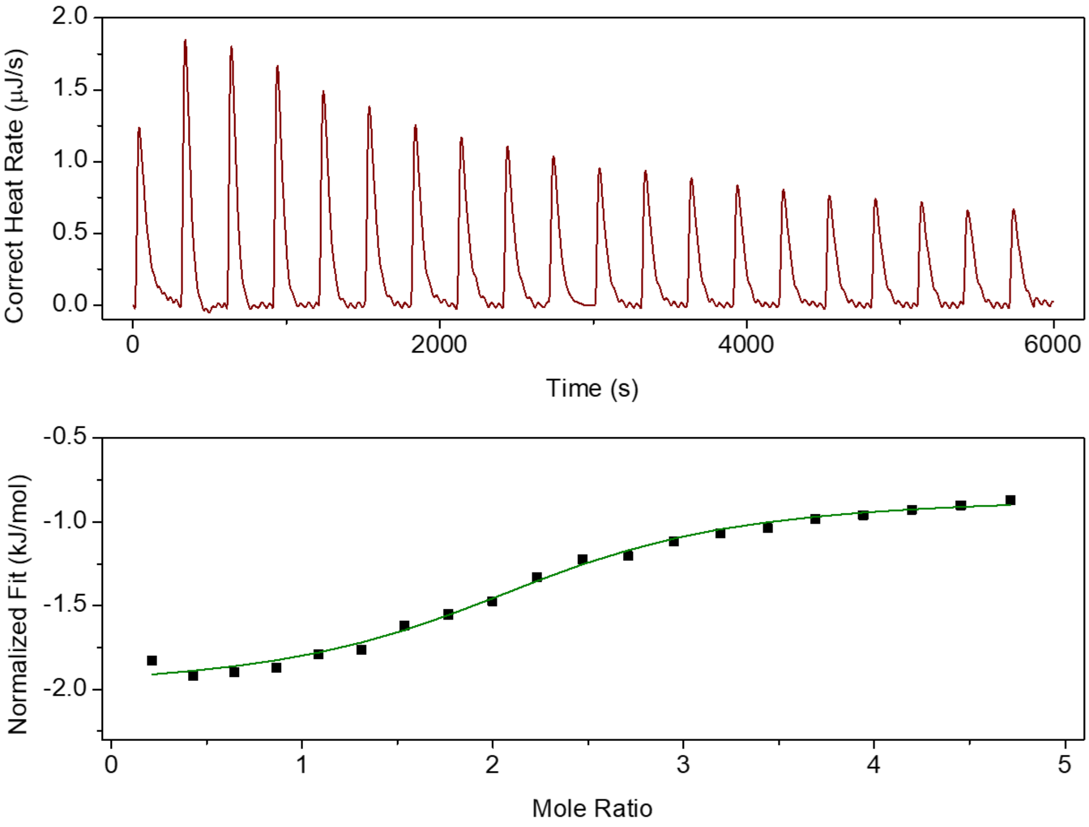

Model	Variable	Value	Confidence Interval (±)
	K_d (M)	4.589E-05	3.278E-06
	n	2.024	0.155
Independent	ΔH (kJ/mol)	-2.411	0.215
	ΔS (J/mol·K)	76.677	
	ΔG (kJ/mol)	-25.261	
	Confidence Level	99%	

Supplementary Figure 41. Microcalorimetric titration of H_F in CH_3CN solution at 298.00 K. (Top) Raw data for sequential 20 injections (10 μL per injection) of 4-hydroxynitrobenzene solution injecting into H_F solution (0.1 mM). (Bottom) Apparent reaction heat obtained from the integration of calorimetric traces.

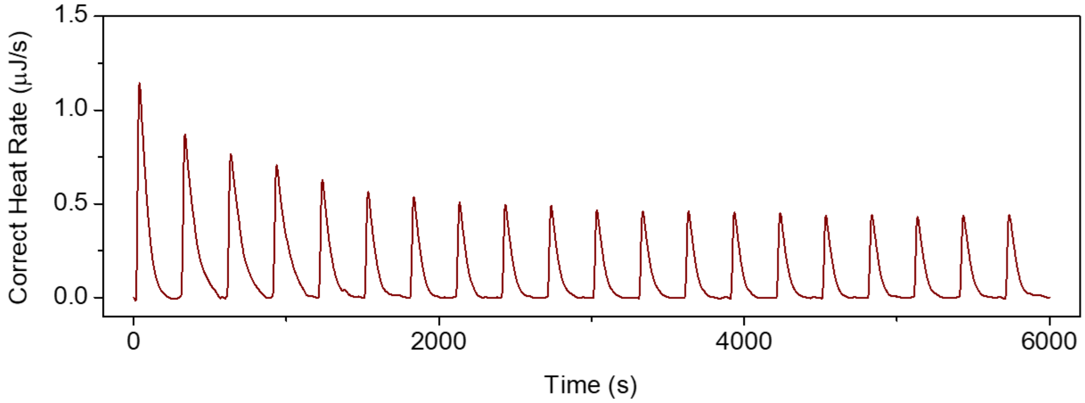

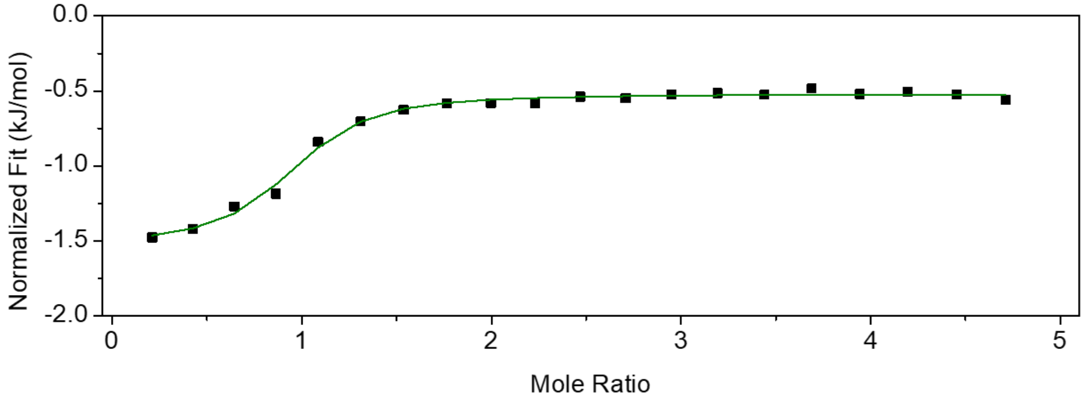

Model	Variable	Value	Confidence Interval (±)
	K_d (M)	2.405E-05	1.721E-06
	n	2.039	0.162
Independent	ΔH (kJ/mol)	-1.054	0.070
	ΔS (J/mol·K)	76.711	
	ΔG (kJ/mol)	-23.914	
	Confidence Level	99%	

Supplementary Figure 42. Microcalorimetric titration of H_F in CH_3CN solution at 298.00 K. (Top) Raw data for sequential 20 injections (10 μL per injection) of 4-hydroxynitrosobenzene solution injecting into H_F solution (0.1 mM). (Bottom) Apparent reaction heat obtained from the integration of calorimetric traces.

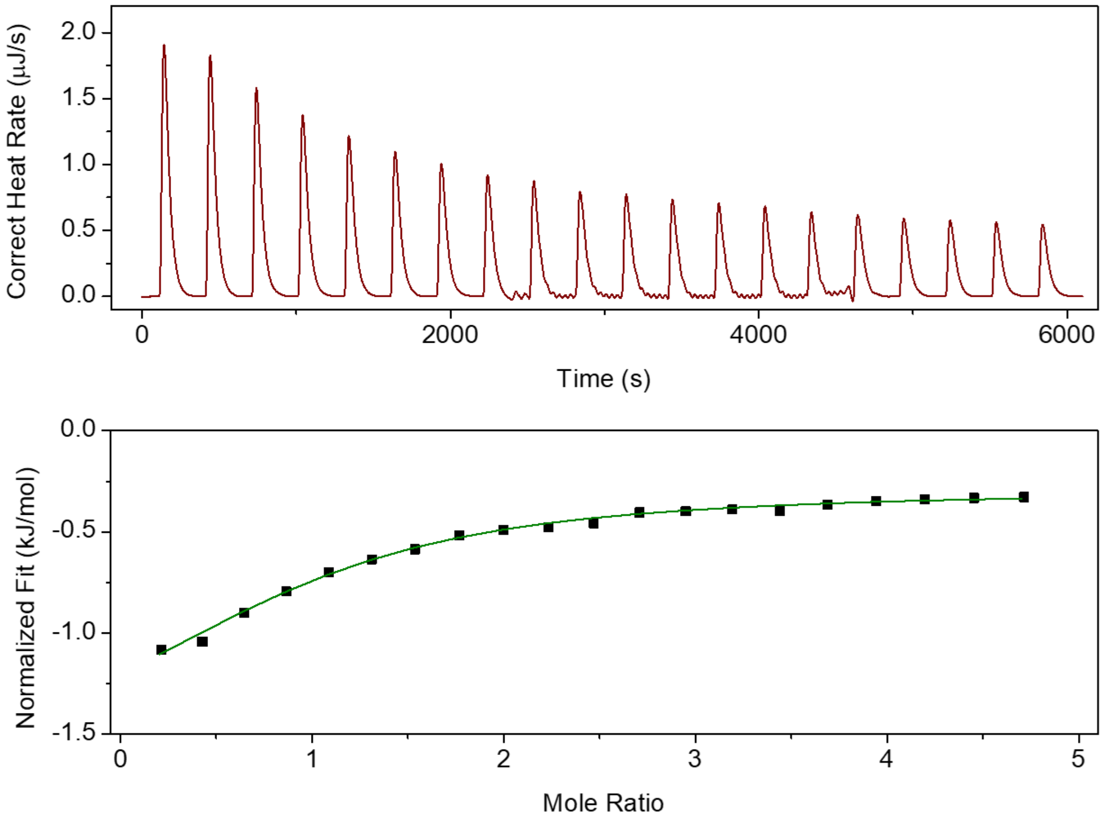

Model	Variable	Value	Confidence Interval (±)
	K_d (M)	1.132E-05	3.995E-06
	n	2.007	0.111
Independent	ΔH (kJ/mol)	-1.843	0.115
	ΔS (J/mol·K)	94.195	
	ΔG (kJ/mol)	-29.913	
	Confidence Level	99%	

Supplementary Figure 43. Microcalorimetric titration of H_F in CH_3CN solution at 298.00 K. (Top) Raw data for sequential 20 injections (10 μL per injection) of 4-hydroxylazanolbenzene solution injecting into H_F solution (0.1 mM). (Bottom) Apparent reaction heat obtained from the integration of calorimetric traces.

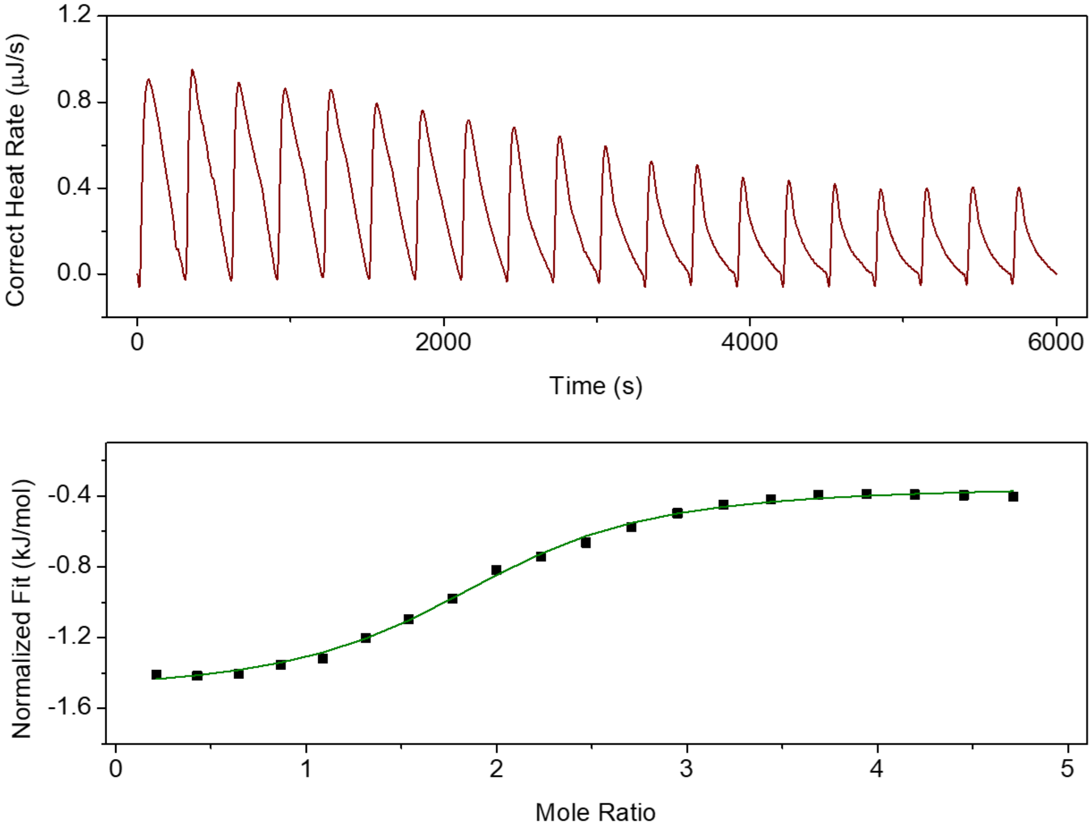

Model	Variable	Value	Confidence Interval (±)
	K_d (M)	6.446E-05	1.338E-06
	n	1.994	0.071
Independent	ΔH (kJ/mol)	-1.181	0.080
	ΔS (J/mol·K)	76.913	
	ΔG (kJ/mol)	-24.101	
	Confidence Level	99%	

Supplementary Figure 44. Microcalorimetric titration of H_F in CH_3CN solution at 298.00 K. (Top) Raw data for sequential 20 injections (10 μL per injection) of 4-hydroxylaminobenzene solution injecting into H_F solution (0.1 mM). (Bottom) Apparent reaction heat obtained from the integration of calorimetric traces.

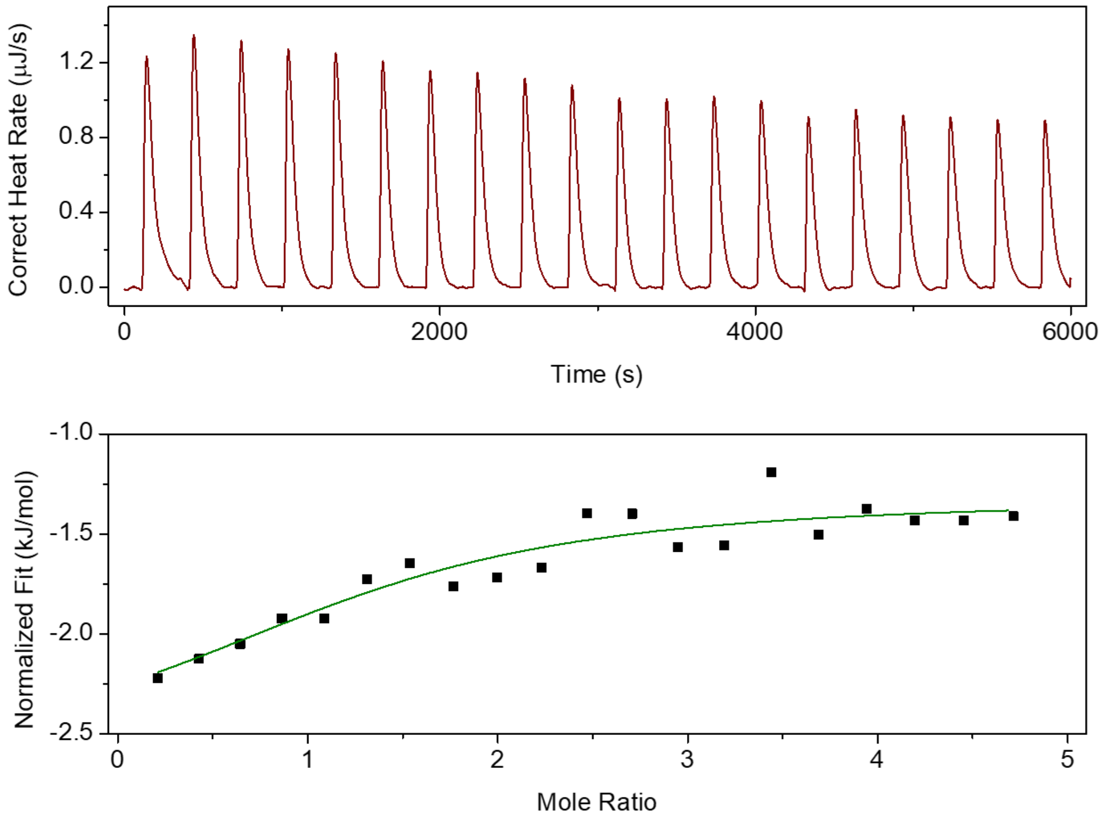

Model	Variable	Value	Confidence Interval (±)
	K_d (M)	6.406E-05	1.601E-06
	n	1.012	0.100
Independent	ΔH (kJ/mol)	-1.134	0.041
	ΔS (J/mol·K)	77.651	
	ΔG (kJ/mol)	-24.273	
	Confidence Level	99%	

Supplementary Figure 45. Microcalorimetric titration of H_F in CH_3CN solution at 298.00 K. (Top) Raw data for sequential 20 injections (10 μL per injection) of 4-hydroxyazobenzene solution injecting into H_F solution (0.1 mM). (Bottom) Apparent reaction heat obtained from the integration of calorimetric traces.

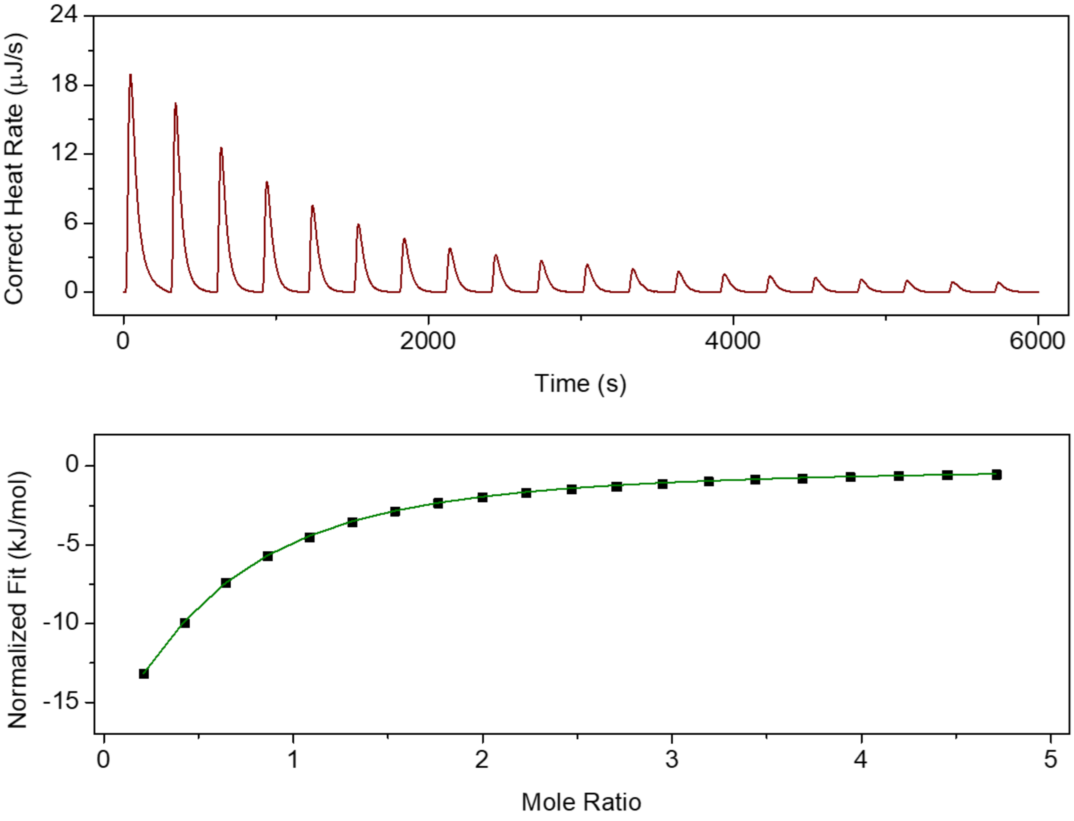

Model	Variable	Value	Confidence Interval (±)
	K_d (M)	3.587E-04	5.593E-05
	n	1.028	0.126
Independent	ΔH (kJ/mol)	-9.610	0.192
	ΔS (J/mol·K)	110.868	
	ΔG (kJ/mol)	-42.649	
	Confidence Level	99%	

Supplementary Figure 46. Microcalorimetric titration of H_F in CH_3CN solution at 298.00 K. (Top) Raw data for sequential 20 injections (10 μL per injection) of 2-carboxynitrobenzene solution injecting into H_F solution (0.1 mM). (Bottom) Apparent reaction heat obtained from the integration of calorimetric traces.

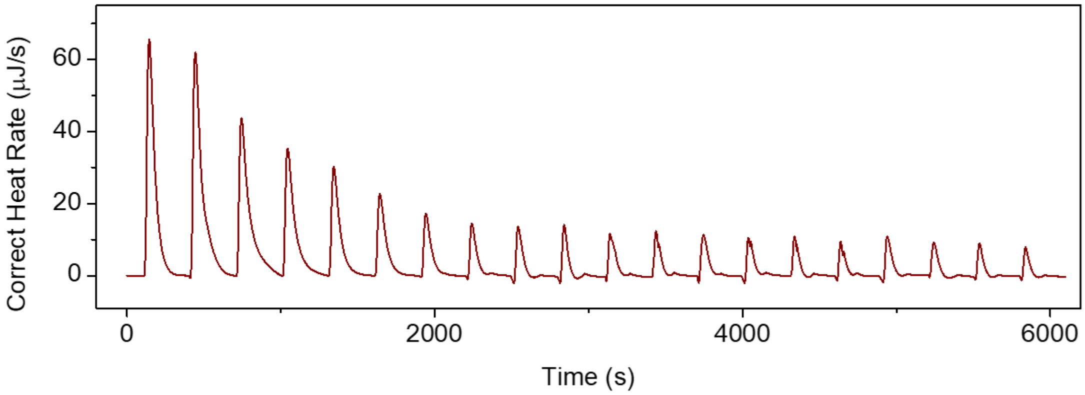

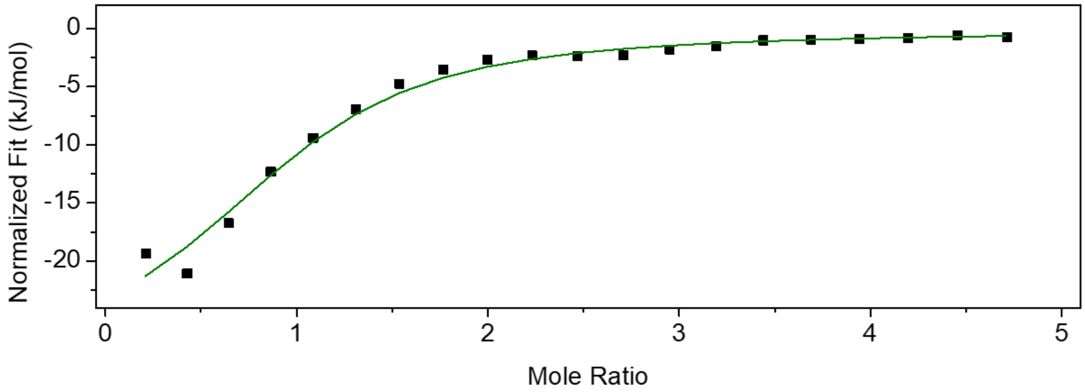

Model	Variable	Value	Confidence Interval (±)
	K_d (M)	1.352E-04	1.671E-05
	n	1.016	0.059
Independent	ΔH (kJ/mol)	-15.703	0.474
	ΔS (J/mol·K)	107.251	
	ΔG (kJ/mol)	-47.664	
	Confidence Level	99%	

Supplementary Figure 47. Microcalorimetric titration of H_F in CH_3CN solution at 298.00 K. (Top) Raw data for sequential 20 injections (10 µL per injection) of 4-sulfonicnitrobenzene solution injecting into H_F solution (0.1 mM). (Bottom) Apparent reaction heat obtained from the integration of calorimetric traces.

13. HPLC data for the catalytic reactions.

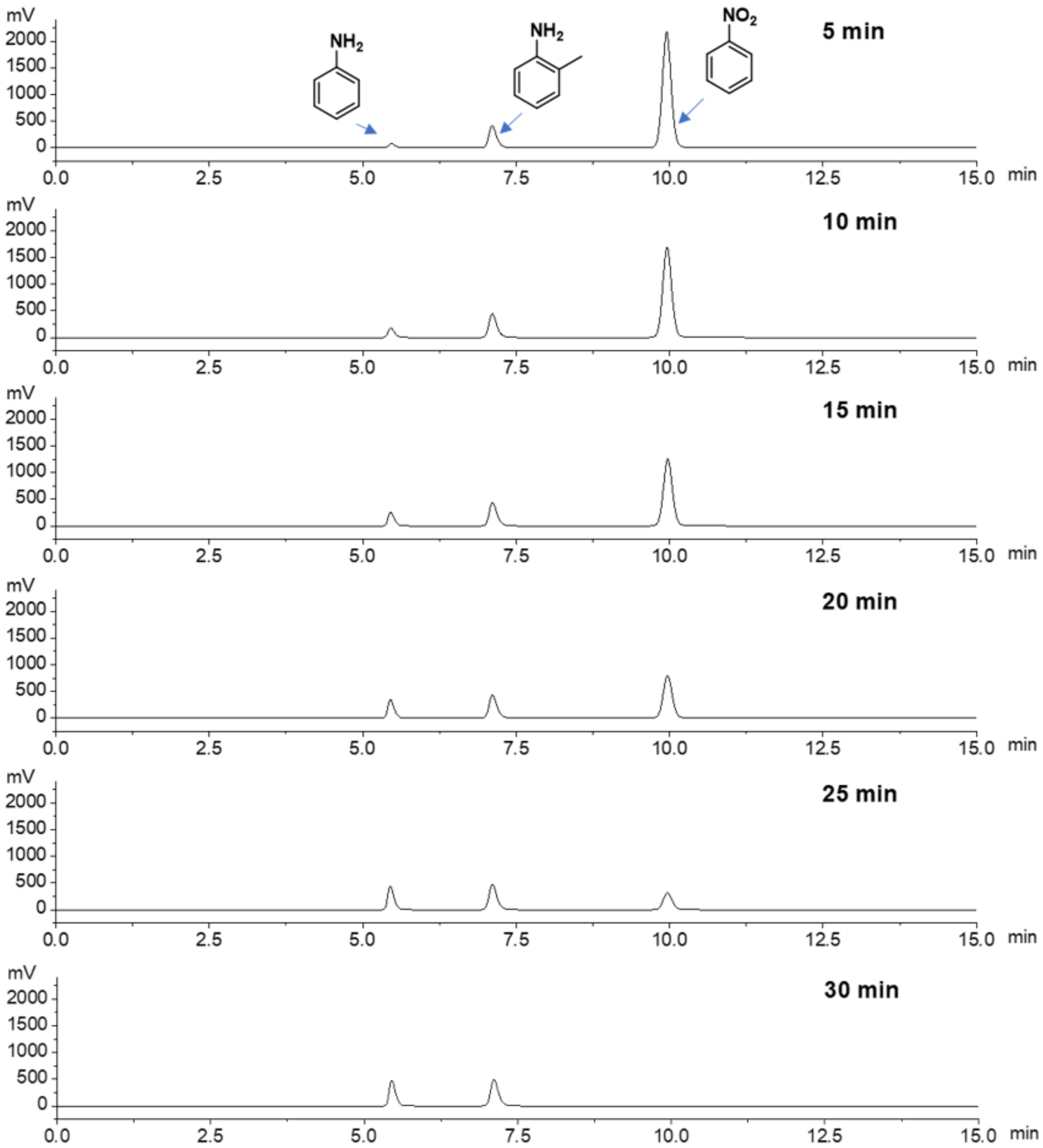

Supplementary Figure 48. HPLC data for the yields of amimobenzene of the photocatalytic reduction of nitrobenzene catalyzed by H_F using m-toluidine as integral standard at 5 min, 10 min, 15 min, 20 min, 25 min, and 30 min, respectively.

Peaks	Retention time (min)	Height	Area	Area (%)
5 min				
1	5.467	78.455	552.942	2.090
2	7.110	415.135	3735.029	14.116
3	9.952	2174.489	22170.858	83.794
10 min				
1	5.459	177.687	1279.793	5.608
2	7.111	444.556	4013.540	17.586
3	9.959	1697.864	17529.201	76.807
15 min				
1	5.456	252.974	1831.359	9.761
2	7.116	434.323	3931.424	20.953
3	9.970	1253.825	12999.938	69.286
20 min				
1	5.452	347.077	2512.578	17.045
2	7.114	429.501	3902.075	26.472
3	9.967	802.787	8325.794	56.483
25 min				
1	5.451	437.702	3165.290	29.520
2	7.113	472.941	4296.265	40.067
3	9.966	313.157	3261.068	30.413
30 min				
1	5.449	478.318	3459.219	43.614
2	7.113	493.106	4472.260	56.386

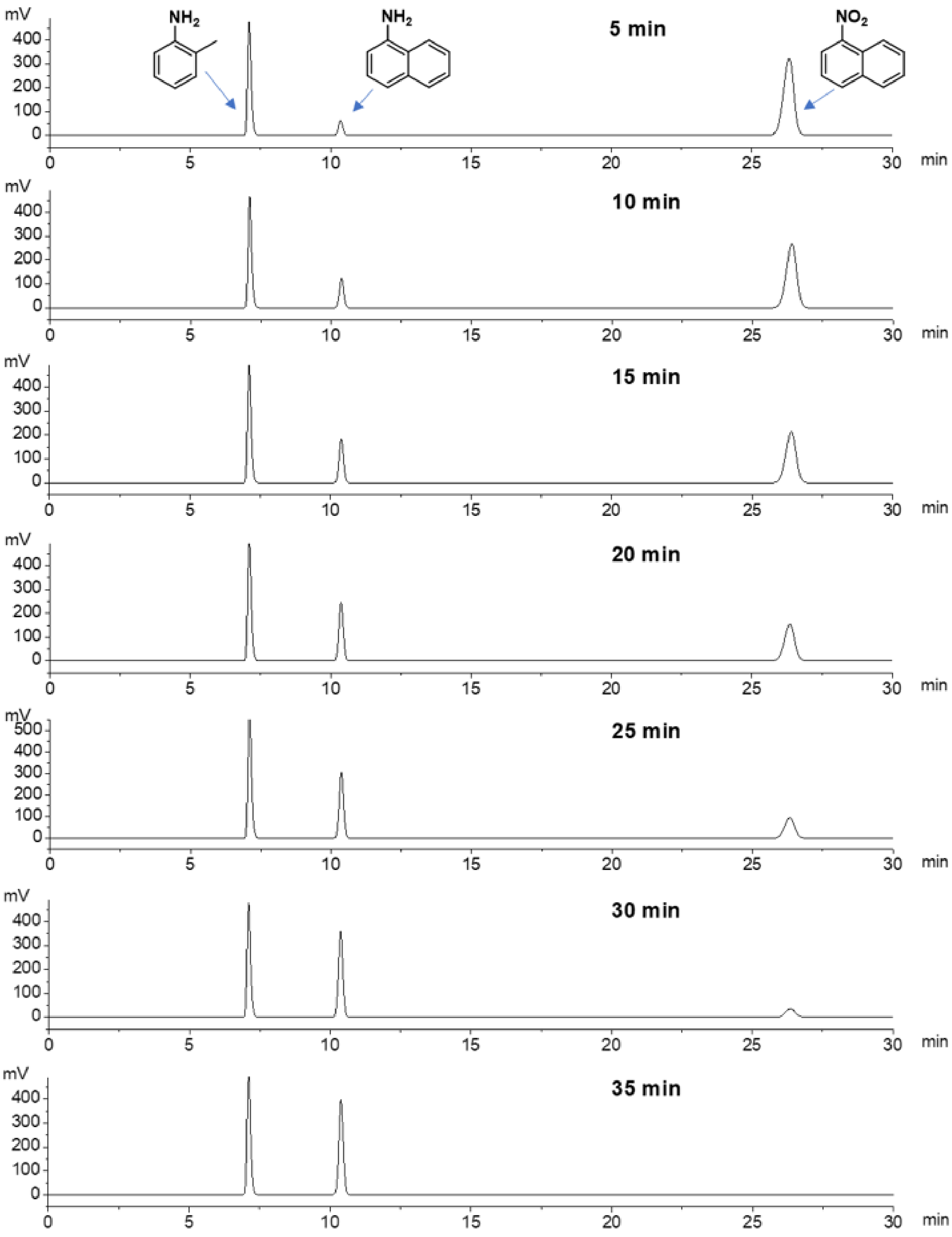

Supplementary Figure 49. HPLC data for the yields of 1-amimonaphthalene of the photocatalytic reduction of 1-nitronaphthalene catalyzed by H_F using m-toluidine as integral standard at 5 min, 10 min, 15 min, 20 min, 25 min, 30 min, and 35 min, respectively.

Peaks	Retention time (min)	Height	Area	Area (%)
5 min				
1	7.093	477.860	4315.595	31.962
2	10.350	63.763	718.788	5.323
3	26.335	322.890	8467.972	62.715
10 min				
1	7.101	467.489	4219.089	33.289
2	10.373	123.930	1411.744	11.139
3	26.408	269.360	7043.481	55.573
15 min				
1	7.103	495.726	4480.878	37.012
2	10.379	182.179	2087.080	17.239
3	26.392	213.574	5538.555	45.749
20 min				
1	7.096	515.871	4656.280	40.627
2	10.366	245.730	2811.597	24.532
3	26.358	153.927	3993.066	34.841
25 min				
1	7.096	623.780	5598.197	48.420
2	10.365	307.761	3525.885	30.496
3	26.332	94.607	2437.745	21.084
30 min				
1	7.096	477.473	4313.699	46.054
2	10.367	359.345	4137.468	44.173
3	26.365	35.664	915.410	9.773
35 min				
1	7.107	502.552	4519.848	49.760
2	10.387	395.527	4563.408	50.240

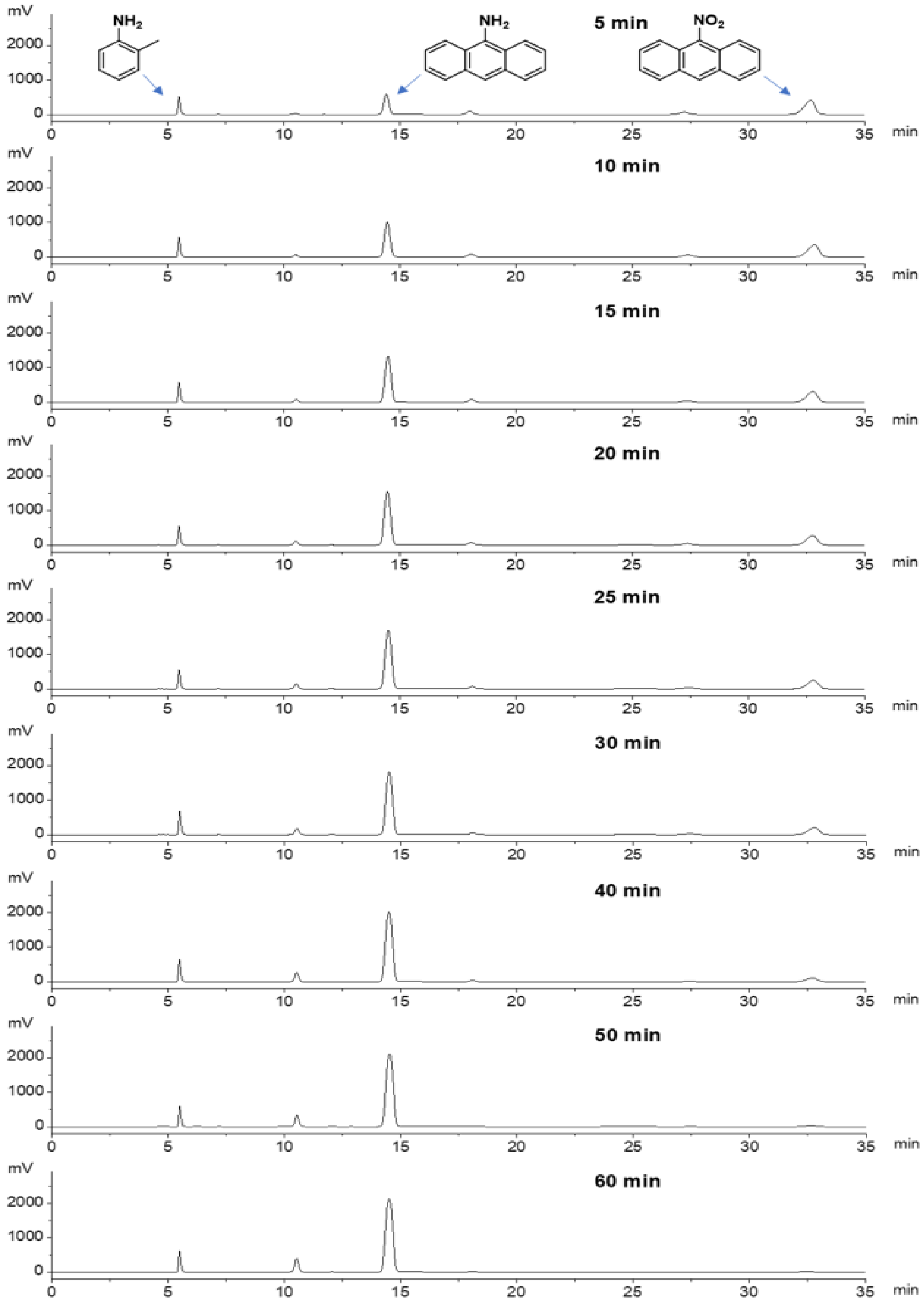

Supplementary Figure 50. HPLC data for the yields of 9-amimoanthracene of the photocatalytic reduction of 9-nitroanthracene catalyzed by H_F using m-toluidine as integral standard at 5 min, 10 min, 15 min, 20 min, 25 min, 30 min, 40 min, 50 min, and 60 min, respectively.

Peaks	Retention time (min)	Height	Area	Area (%)
5 min				
1	5.495	529.557	3834.908	14.219
2	14.423	589.553	9141.162	33.894
3	32.683	415.504	13993.879	51.887
10 min				
1	5.496	586.590	4215.702	12.561
2	14.461	1011.497	17030.644	50.744
3	32.848	365.114	12315.654	36.695
15 min				
1	5.498	586.324	4203.362	10.822
2	14.491	1335.966	24002.032	61.793
3	32.782	317.467	10637.070	27.385
20 min				
1	5.497	559.309	4018.514	9.552
2	14.468	1548.920	28808.166	68.477
3	32.770	274.942	9242.972	21.971
25 min				
1	5.498	563.734	4048.067	9.020
2	14.507	1689.075	32362.525	72.115
3	32.807	252.556	8465.803	18.864
30 min				
1	5.497	681.950	4880.414	10.241
2	14.501	1822.553	35772.768	75.067
3	32.778	208.381	7001.284	14.692
40 min				
1	5.497	641.397	4611.263	9.236
2	14.508	2011.018	41602.674	83.327
3	32.727	107.768	3712.863	7.437
50 min				
1	5.499	602.194	4313.904	8.526
2	14.519	2105.233	45099.096	89.133
3	32.622	31.329	1184.270	2.341
60 min				
1	5.501	618.913	4438.586	8.863
2	14.517	2117.765	45641.469	91.137

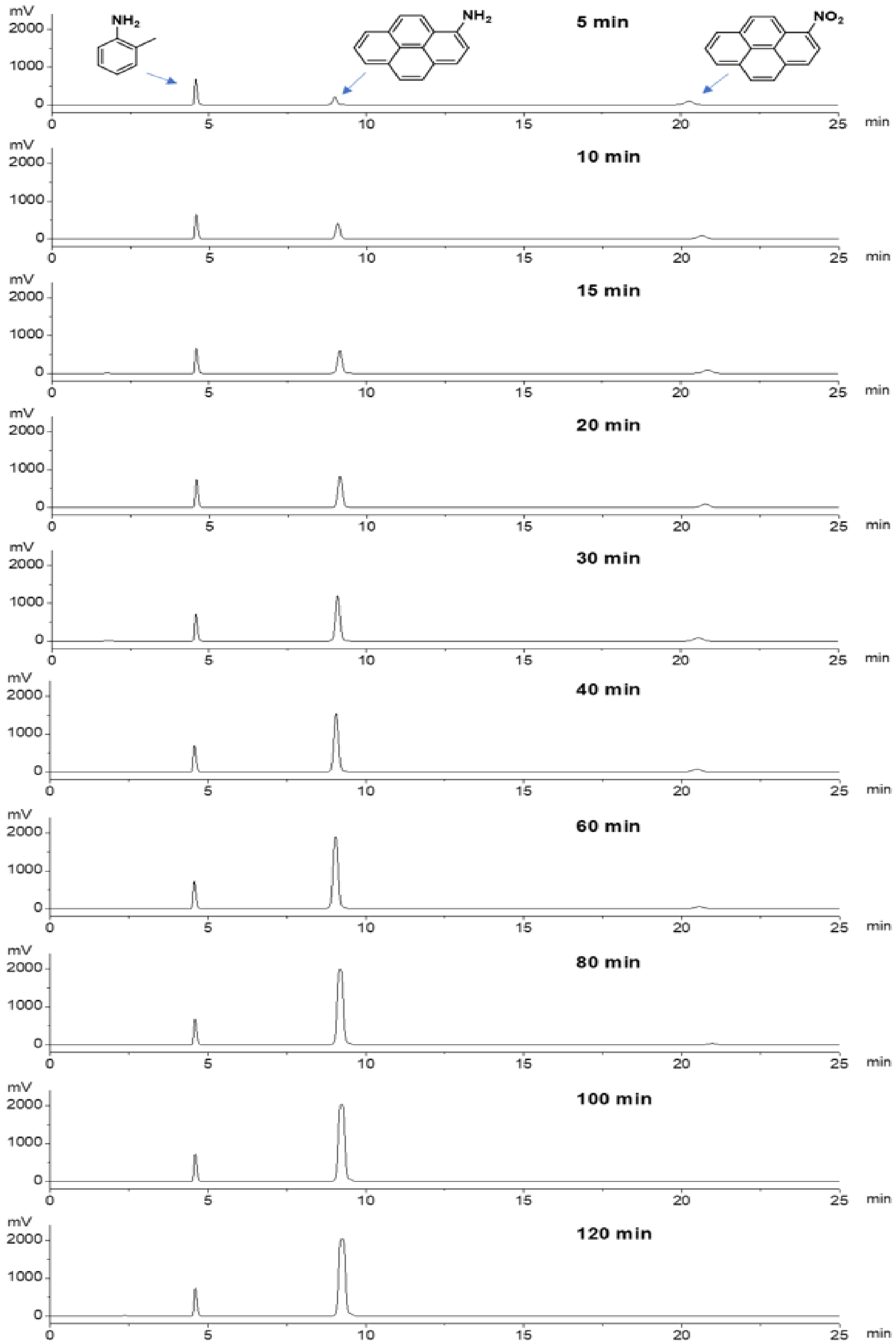

Supplementary Figure 51. HPLC data for the yields of 1-aminopyrene of the photocatalytic reduction of 1-nitropyrene catalyzed by H_F using m-toluidine as integral standard at 5 min, 10 min, 15 min, 20 min, 30 min, 40 min, 60 min, 80 min, 100 min, and 120 min, respectively.

Peaks	Retention time (min)	Height	Area	Area (%)
5 min				
1	4.574	700.029	4229.270	50.869
2	8.996	215.490	2126.849	25.581
3	20.261	98.183	1958.003	23.550
10 min				
1	4.579	653.405	3976.404	39.457
2	9.080	420.217	4245.677	42.129
3	20.654	91.229	1855.694	18.414
15 min				
1	4.590	665.493	4044.555	33.841
2	9.153	600.029	6101.624	51.052
3	20.855	88.349	1805.535	15.107
20 min				
1	4.590	739.846	4476.475	30.661
2	9.152	821.103	8356.633	57.238
3	20.760	88.171	1766.666	12.101
30 min				
1	4.579	717.426	4373.685	23.558
2	9.085	1206.884	12461.741	67.122
3	20.568	86.390	1730.501	9.321
40 min				
1	4.576	712.955	4302.147	19.331
2	9.060	1547.141	16398.472	73.683
3	20.490	78.235	1554.682	6.986
60 min				
1	4.573	733.141	4418.597	15.907
2	9.043	1908.626	22329.666	80.386
3	20.567	51.452	1029.880	3.708
80 min				
1	4.594	680.847	4149.068	13.460
2	9.190	2001.776	26081.730	84.612
3	20.972	29.077	594.123	1.927
100 min				
1	4.601	733.363	4450.667	13.358
2	9.237	2036.200	28725.912	86.217
3	21.092	7.743	141.585	0.425
120 min				
1	4.604	737.734	4474.641	13.118
2	9.254	2044.526	29636.016	86.882

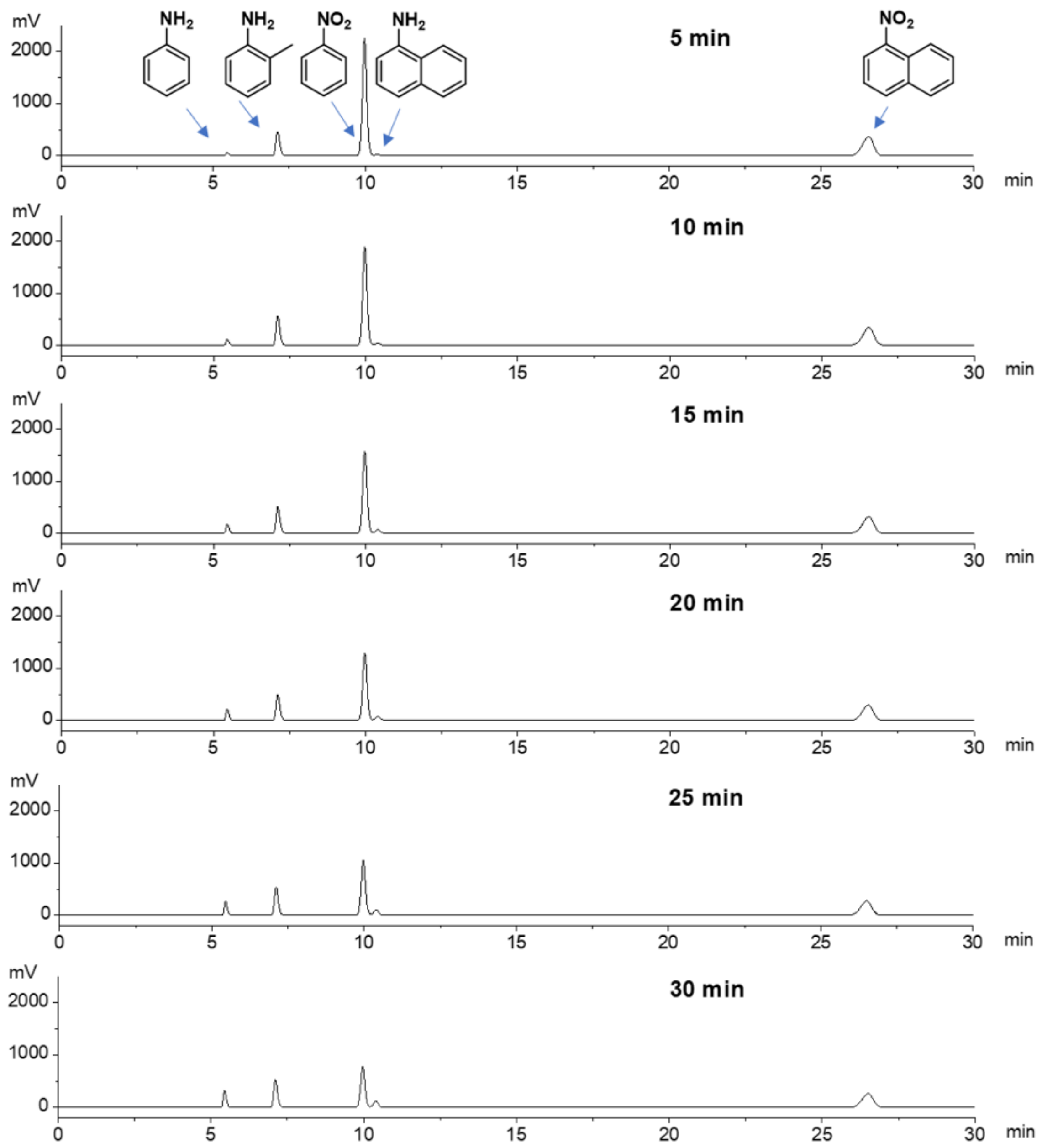

Supplementary Figure 52. HPLC data for yield of amino products of the competing photocatalytic reduction of nitrobenzene and 1-nitronaphthalene catalyzed by H_F using m-toluidine as integral standard at 5 min, 10 min, 15 min, 20 min, 25 min, and 30 min, respectively.

Peaks	Retention time (min)	Height	Area	Area (%)
5 min				
1	5.463	60.934	430.350	1.166
2	7.118	469.114	4176.509	11.319
3	9.978	2240.183	22643.793	61.367
4	10.404	13.869	121.675	0.330
5	26.555	362.643	9526.908	25.819
10 min				
1	5.457	124.218	887.719	2.568
2	7.112	577.959	5144.518	14.881
3	9.973	1889.046	19349.279	55.970
4	10.401	27.468	220.545	0.638
5	26.541	341.825	8968.673	25.943
15 min				
1	5.456	178.003	1270.774	4.100
2	7.115	512.976	4558.770	14.708
3	9.973	1576.788	16188.388	52.229
4	10.401	59.657	596.707	1.925
5	26.534	318.494	8380.574	27.038
20 min				
1	5.452	228.462	1638.180	5.874
2	7.114	506.644	4514.856	16.190
3	9.973	1290.418	13224.886	47.424
4	10.401	74.069	723.922	2.596
5	26.521	296.659	7784.900	27.916
25 min				
1	5.449	277.205	1987.852	7.654
2	7.111	540.810	4809.963	18.520
3	9.972	1060.106	10877.807	41.883
4	10.400	96.073	982.309	3.782
5	26.507	278.562	7314.157	28.162
30 min				
1	5.450	323.072	2317.453	10.149
2	7.112	526.706	4686.102	20.522
3	9.970	770.092	7830.750	34.293
4	10.396	108.585	1093.701	4.790
5	26.505	262.839	6906.654	30.246

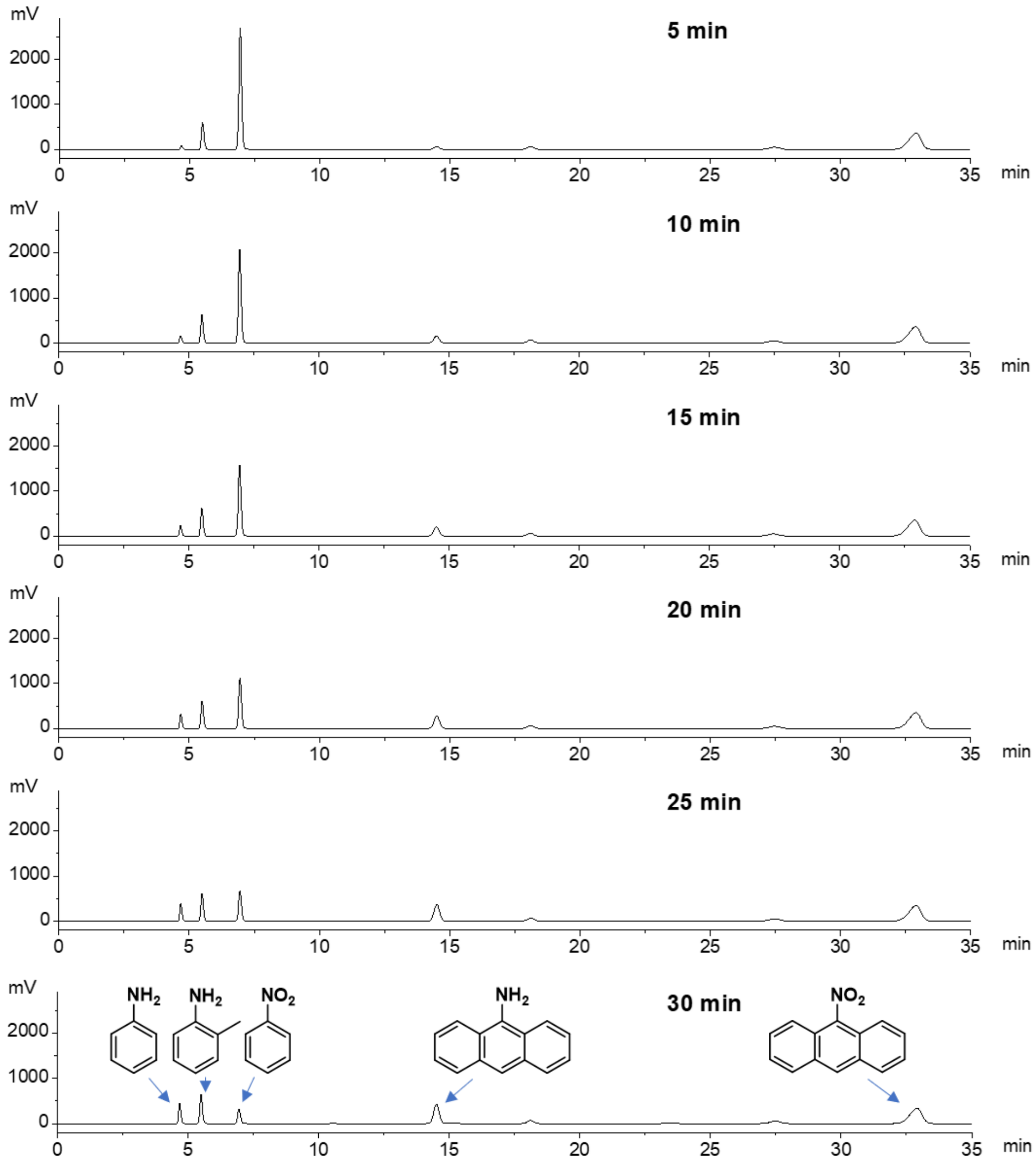

Supplementary Figure 53. HPLC data for yield of amino products of the competing photocatalytic reduction of nitrobenzene and 9-nitroanthracene catalyzed by H_F using m-toluidine as integral standard at 5 min, 10 min, 15 min, 20 min, 25 min, and 30 min, respectively.

Peaks	Retention time (min)	Height	Area	Area (%)
5 min				
1	4.687	87.119	554.834	1.456
2	5.495	600.982	4312.020	11.317
3	6.944	2690.155	20113.360	52.788
4	14.496	64.371	919.305	2.413
5	32.921	362.343	12202.758	32.026
10 min				
1	4.680	164.260	1016.586	2.842
2	5.495	623.906	4338.190	12.129
3	6.950	2078.677	15918.165	44.506
4	14.508	155.957	2296.588	6.421
5	32.911	362.246	12196.797	34.101
15 min				
1	4.677	240.876	1491.587	4.522
2	5.492	625.658	4348.710	13.184
3	6.946	1581.073	12235.530	37.095
4	14.502	201.620	2981.265	9.039
5	32.878	356.076	11926.870	36.160
20 min				
1	4.678	318.085	1970.411	6.365
2	5.493	612.084	4253.491	13.739
3	6.948	1116.457	8632.055	27.882
4	14.502	283.052	4258.131	13.754
5	32.894	352.876	11844.945	38.260
25 min				
1	4.676	390.601	2417.908	8.273
2	5.491	616.686	4291.091	14.682
3	6.946	676.301	5259.944	17.997
4	14.504	371.053	5660.879	19.369
5	32.892	345.605	11596.222	39.678
30 min				
1	4.676	449.836	2795.642	10.059
2	5.493	652.523	4552.276	16.383
3	6.948	317.399	2477.854	8.916
4	14.506	424.937	6507.815	23.416
5	32.899	341.439	11457.956	41.227

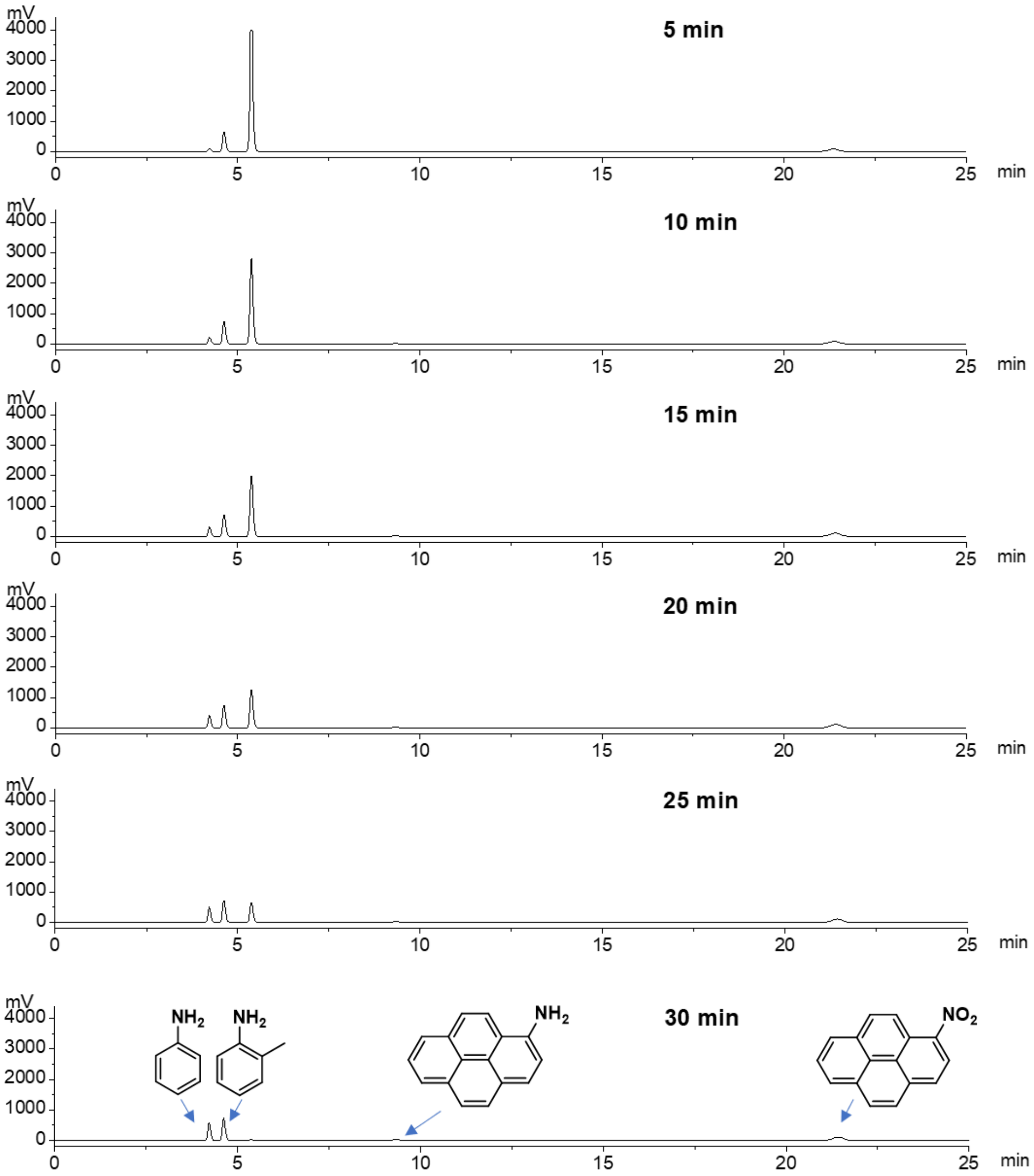

Supplementary Figure 54. HPLC data for yield of amino products of the competing photocatalytic reduction of nitrobenzene and 1-nitropyrene catalyzed by H_F using m-toluidine as integral standard at 5 min, 10 min, 15 min, 20 min, 25 min, and 30 min, respectively.

Peaks	Retention time (min)	Height	Area	Area (%)
5 min				
1	4.224	101.748	554.447	1.756
2	4.622	652.003	3850.804	12.199
3	5.354	3998.129	25520.511	80.844
4	21.362	80.956	1641.707	5.201
10 min				
1	4.224	215.016	1179.400	4.896
2	4.622	730.465	4325.243	17.953
3	5.373	2813.039	16744.151	69.502
4	9.342	14.249	130.008	0.540
5	21.375	83.515	1712.676	7.109
15 min				
1	4.224	306.968	1689.271	8.111
2	4.623	720.992	4274.347	20.523
3	5.375	2002.650	12314.632	59.127
4	9.332	23.414	229.863	1.104
5	21.412	111.840	2319.407	11.136
20 min				
1	4.221	407.163	2264.916	13.210
2	4.620	740.645	4408.464	25.713
3	5.373	1261.519	7819.578	45.608
4	9.333	32.049	318.295	1.856
5	21.426	111.785	2333.943	13.613
25 min				
1	4.220	499.668	2771.063	19.874
2	4.620	722.598	4292.972	30.789
3	5.373	664.041	4115.767	29.518
4	9.335	39.475	397.167	2.848
5	21.434	112.939	2366.372	16.971
30 min				
1	4.220	593.332	3297.966	31.223
2	4.620	721.194	4289.112	40.606
3	5.373	32.459	197.342	1.868
4	9.334	43.603	434.638	4.115
5	21.430	112.041	2343.574	22.187

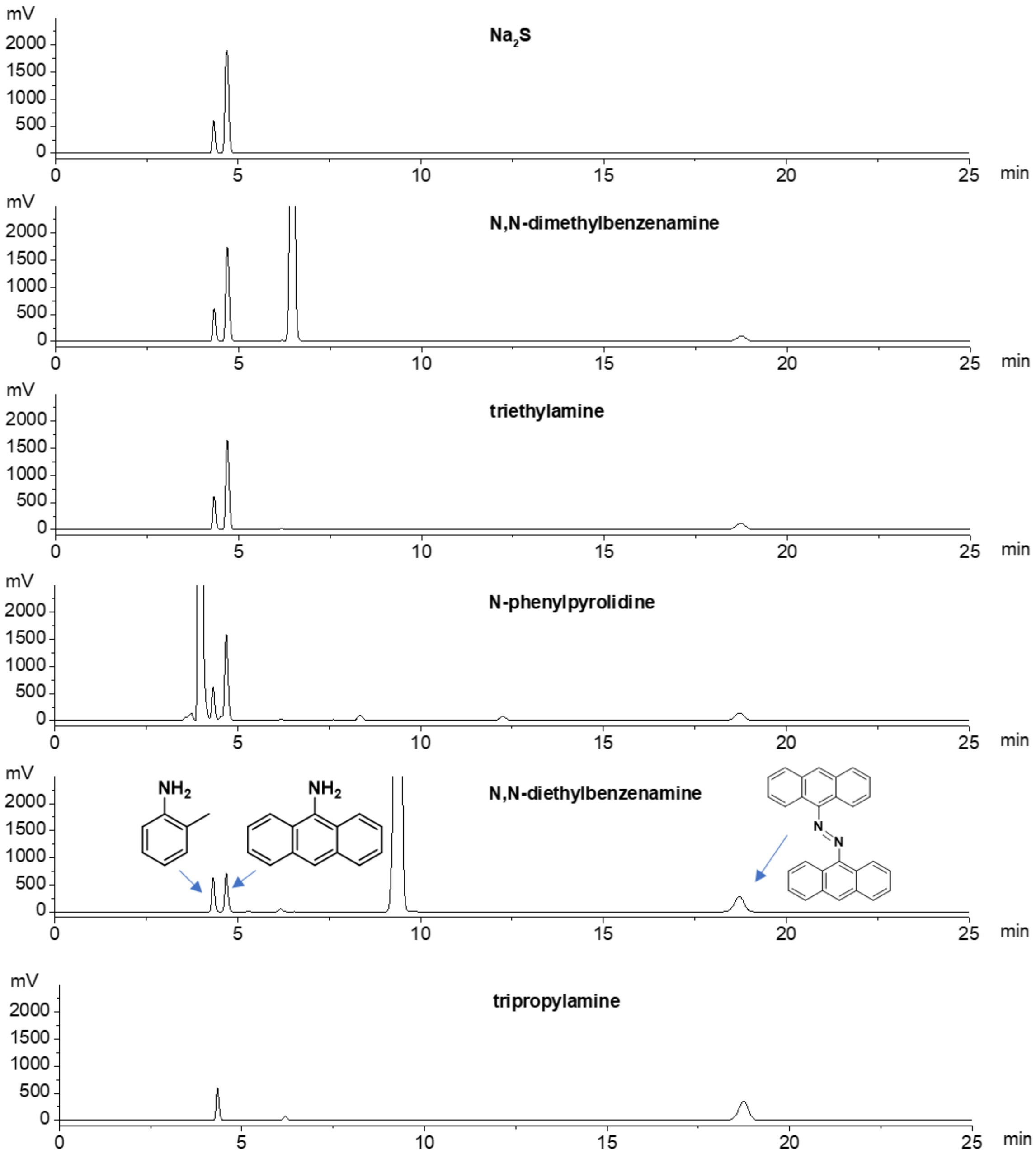

Supplementary Figure 55. HPLC data for yields of amino and azo products of the photocatalytic reduction of 9-nitroanthracene catalyzed by H_F using m-toluidine as integral standard with different reductans (Na_2S, N,N-dimethylbenzenamine, triethylamine, N-phenylpyrolidine, N,N-diethylbenzenamine, and tripropylamine, respectively).

Peaks	Retention time (min)	Height	Area	Area (%)
Na_2S				
1	4.327	604.649	3344.541	19.877
2	4.688	1894.961	13481.994	80.123
N,N-dimethylbenzenamine				
1	4.332	598.951	3314.484	19.822
2	4.694	1740.840	11540.370	69.018
3	18.750	99.873	1866.064	11.160
triethylamine				
1	4.331	610.024	3368.381	20.928
2	4.693	1642.801	10481.262	65.122
3	18.731	117.523	2245.211	13.950
N-phenylpyrolidine				
1	4.330	571.654	2928.918	19.164
2	4.690	1557.863	9715.668	63.569
3	18.728	141.103	2639.072	17.267
N,N-diethylbenzenamine				
1	4.330	629.449	3504.354	26.469
2	4.690	713.958	4287.851	32.387
3	18.724	285.682	5447.288	41.144
tripropylamine				
1	4.329	595.776	3328.525	33.001
2	18.745	350.858	6757.691	66.999

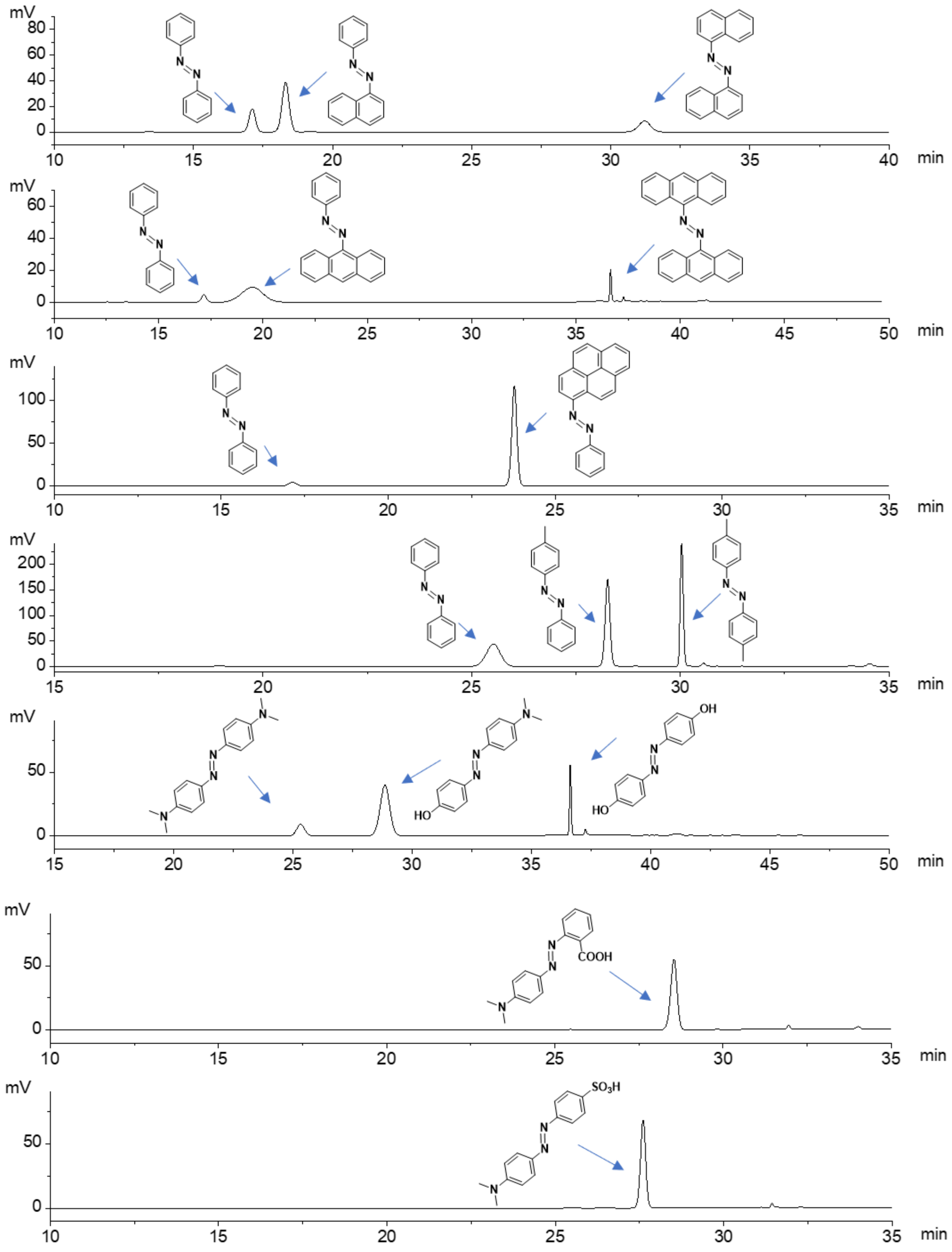

Supplementary Figure 56. HPLC data for yield and selectivity of asymmetric azo product of the photocatalytic reduction of the mixed nitro substrates catalyzed by H_F using tripropylamine as reductant.

Peaks	Retention time (min)	Height	Area	Area (%)
nitrobenzene + 1-nitronaphthalene				
1	17.112	18.184	302.849	23.360
2	18.312	38.917	723.110	55.775
3	31.214	8.576	270.515	20.865
nitrobenzene + 9-nitroanthracene				
1	17.168	4.959	84.075	9.361
2	19.488	9.474	709.516	80.001
3	36.657	20.175	104.515	10.637
nitrobenzene + 1-nitropyrene				
1	17.138	4.577	134.533	9.839
2	23.779	117.030	1296.673	90.161
nitrobenzene + 4-methylnitrobenzene				
1	25.521	43.950	1079.700	28.912
2	28.258	170.996	1410.831	37.778
3	30.032	241.146	1243.965	33.310
N,N-dimethyl-4-nitrobenzene + 4-hydroxynitrobenzene				
1	25.331	9.172	224.337	13.521
2	28.863	39.822	1197.775	72.190
3	36.636	54.906	237.077	14.289
N,N-dimethyl-4-nitrobenzene + 2-carboxynitrobenzene				
1	25.333	2.327	38.861	4.89
2	28.541	54.802	755.841	95.110
N,N-dimethyl-4-nitrobenzene + 4-sulfonicnitrobenzene				
1	27.607	68.284	757.570	99.400

14. Characterization of the azo products.

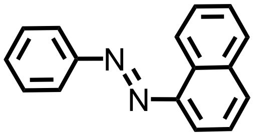

1-(1-Naphthalenyl)-2-phenyldiazene

The product is obtained as dark red solid. ^{1}H NMR (400 MHz, DMSO-d_6) δ 8.45 (d, 1 H), 7.97 (d, 2 H), 7.80-7.88 (m, 1 H), 7.63-7.49 (m, 6 H), 7.45-7.42 (m, 1 H), 7.11 (d, 1 H); ^{13}C NMR (100 MHz, DMSO-d_6) δ 152.81, 150.90, 134.22, 131.05, 130.51, 129.00, 128.53, 127.44, 126.30, 123.02, 116.51. HRMS-ESI (m/z): $[M+H]^+$ calcd. for $C_{16}H_{13}N_2^+$, 233.1079; found, 233.1080.

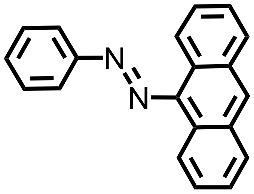

1-(9-Anthracenly)-2-phenyldiazene

The product is obtained as orange solid. ^{1}H NMR (400 MHz, DMSO-d_6) δ 8.67 (s, 1 H), 8.20 (m, 2 H), 8.03-7.92 (m, 4 H), 7.58-7.41 (m, 6 H), 7.18 (m, 1 H); ^{13}C NMR (100 MHz, DMSO-d_6) δ 153.23, 151.43, 137.63, 134.52, 133.78, 132.99, 132.12, 131.30, 130.02, 129.70, 128.79, 127.26, 126.71, 126.36, 125.96, 123.61, 117.55. HRMS-ESI (m/z): $[M+H]^+$ calcd. for $C_{20}H_{15}N_2^+$, 283.1235; found, 283.1233.

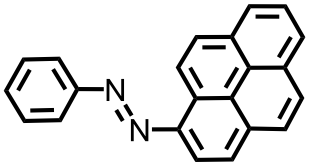

1-phenyl-2-(1-pyrenyl)diazene

The product is obtained as red solid. ^{1}H NMR (400 MHz, DMSO-d_6) δ 8.77 (d, 1 H), 8.63 (d, 1 H), 8.19 (d, 1 H), 7.93-7.76 (m, 4 H), 7.66 (m, 1 H), 7.54-7.45 (m, 3H), 7.16-7.07 (m, 2H), 6.95-6.89 (m, 1H); ^{13}C NMR (100 MHz, DMSO-d_6) δ 152.71, 150.31, 133.34, 131.22,

130.95, 130.35, 129.07, 128.35, 126.60, 126.37, 125.41, 123.02, 122.44, 121.50. HRMS-ESI (m/z): $[M+H]^+$ calcd. for $C_{22}H_{15}N_2^+$, 307.1235; found, 307.1237.

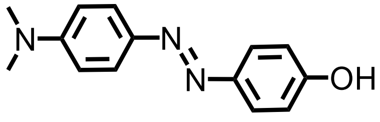

4-Hydroxy-4'-(dimethylamino)azobenzene

The product is obtained as red solid. ^{1}H NMR (400 MHz, DMSO-d_6) δ 10.17 (s, 1 H), 7.79-7.77 (m, 4 H), 7.10-7.07 (d, 2 H), 6.89 (d, 2 H), 3.10 (m, 6 H); ^{13}C NMR (100 MHz, DMSO-d_6) δ 161.10, 153.50, 145.33, 144.82, 125.17, 124.41, 116.52, 111.67. HRMS-ESI (m/z): $[M+Na]^+$ calcd. for $C_{14}H_{15}N_3ONa^+$, 264.1113; found, 264.1110.

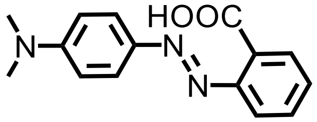

2-Carboxy-4'-(dimethylamino)azobenzene

The product is obtained as dark red solid. ^{1}H NMR (400 MHz, DMSO-d_6) δ 13.16 (s, 1 H), 7.77 (m, 3 H), 7.65-7.62 (m, 2 H), 7.51-7.45 (m, 1 H), 6.38 (d, 2 H), 3.09 (s, 6 H); ^{13}C NMR (100 MHz, DMSO-d_6) δ 167.30, 153.92, 150.31, 141.72, 133.44, 132.40, 126.95, 125.11, 115.62, 111.37. HRMS-ESI (m/z): $[M+Na]^+$ calcd. for $C_{15}H_{15}N_3O_2Na^+$, 292.1062; found, 292.1064.

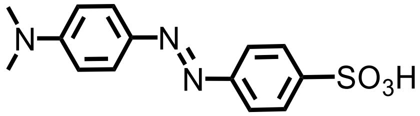

4-sulfonic-4'-(dimethylamino)azobenzene

The product is obtained as orange solid. ^{1}H NMR (400 MHz, DMSO-d_6) δ 7.80 (d, 2 H), 7.74-7.70 (m, 4 H), 6.87 (d, 2 H), 3.07 (m, 6 H); ^{13}C NMR (100 MHz, DMSO-d_6) δ 157.01, 153.22, 147.61, 144.80, 125.21, 123.27, 111.71. HRMS-ESI (m/z): $[M+Na]^+$ calcd. for $C_{14}H_{15}N_3O_3SNa^+$, 328.0732; found, 328.0734.

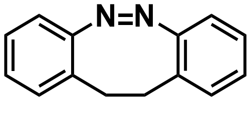

(Z)-11,12-Dihydrodibenzo[c,g][1,2]diazocine

The product is obtained as yellow solid. ^{1}H NMR (400 MHz, $CDCl_3$) δ 7.27-7,21 (m, 4 H), 7.16-7.01 (m, 2 H), 6.97-6.95 (m, 2 H), 3.07-2.95 (m, 4 H); ^{13}C NMR (100 MHz, DMSO-d_6) δ 149.09, 132.32, 131.73, 130.99, 130.78, 121.99, 30.68. HRMS-ESI (m/z): $[M+H]^+$ calcd. for $C_{14}H_{13}N_2^+$, 209.1079; found, 209.1080.

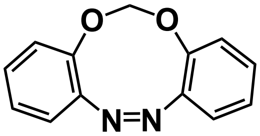

(Z)-dibenzo[*d*,*h*][1,3,6,7]dioxadiazonine

The product is obtained as yellow solid. ^{1}H NMR (400 MHz, $CDCl_3$) δ 7.91 (d, 2 H), 7.75-7.70 (m, 2 H), 7.58-7.55 (m, 2 H), 7.28-7.24 (m, 2 H) 6,17 (s, 2 H); ^{13}C NMR (100 MHz, DMSO-d_6) δ 148.73, 140.88, 134.78, 125.47, 123.40, 117.60, 91.35. HRMS-ESI (m/z): $[M+H]^+$ calcd. for $C_{13}H_{11}N_2O_2^+$, 227.0820; found, 227.0823.

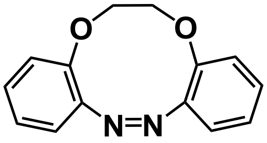

(Z)-6,7-dihydrodibenzo[*e*,*i*][1,4,7,8]dioxadiazecine

The product is obtained as yellow solid. ^{1}H NMR (400 MHz, $CDCl_3$) δ 7.87 (d, 2 H), 7.68-7.64 (m, 2 H), 7.45 (d, 2 H), 7,17-7.13 (m, 2 H) 4.54 (s, 4 H); ^{13}C NMR (100 MHz, DMSO-d_6) δ 151.39, 140.25, 134.80, 125.34, 121.48, 116.05, 68.52. HRMS-ESI (m/z): $[M+H]^+$ calcd. for $C_{14}H_{13}N_2O_2^+$, 241.0977; found, 241.0976.

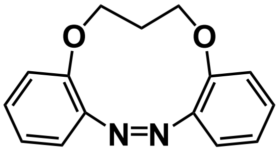

(Z)-7,8-dihydro-6H-dibenzo[*b*,*f*][1,8]dioxa[4,5]diazacycloundecine

The product is obtained as yellow solid. ^{1}H NMR (400 MHz, $CDCl_3$) δ 7.88 (d, 2 H), 7.68-7.64 (m, 2 H), 7.40 (m, 2 H), 7.14-7.10 (m, 2 H), 4.33 (m, 4 H) 2.22 (s, 2 H); ^{13}C NMR (100 MHz, DMSO-d_6) δ 151.14, 139.45, 134.53, 124.97, 120.60, 114.97, 65.45, 28.06. HRMS-ESI (m/z): $[M+H]^+$ calcd. for $C_{15}H_{15}N_2O_2^+$, 255.1133; found, 255.1135.

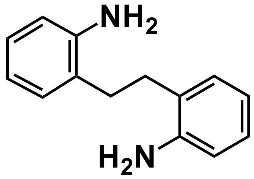

2,2’-(ethane-1,2-diyl)dianiline

The product is obtained as white solid. ^{1}H NMR (400 MHz, $CDCl_3$) δ 7.01-6.98 (dd, 2 H), 6.92-6.88 (m, 2 H), 6.64-6.62 (m, 2 H), 6.52-6.49 (m, 2 H), 4.84 (s, 4 H), 2.66 (s, 4 H); ^{13}C NMR (100 MHz, DMSO-d_6) δ 146.55, 129.38, 126.88, 125.84, 116.79, 115.12, 30.25. HRMS-ESI (m/z): $[M+H]^+$ calcd. for $C_{14}H_{17}N_2^+$, 213.1392; found, 213.1390.

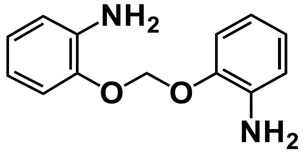

2-[(2-Aminophenoxy)methoxy]aniline

The product is obtained as pale yellow solid. ^{1}H NMR (400 MHz, $CDCl_3$) δ 7.10-7.08 (d, 2 H), 6.81-6.77 (m, 2 H), 6.71-6.69 (m, 2 H), 6.56-6.52 (m, 2 H), 5.71 (s, 2 H), 4.80 (s, 4 H); ^{13}C NMR (100 MHz, DMSO-d_6) δ 144.01, 139.18, 123.33, 116.61, 115.80, 115.15, 92.46. HRMS-ESI (m/z): $[M+H]^+$ calcd. for $C_{13}H_{15}N_2O_2^+$, 231.1133; found, 231.1134.

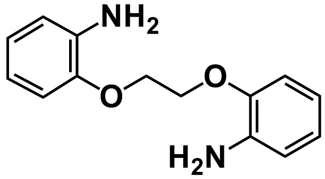

2,2’-(Ethylenedioxy)dianiline

The product is obtained as pale yellow solid. ^{1}H NMR (400 MHz, $CDCl_3$) δ 6.88 (d, 2 H), 6.72-6.64 (m, 4 H), 6.54-6.50 (m, 2 H), 4.69 (s, 4 H), 4.28 (s, 4 H); ^{13}C NMR (100 MHz, DMSO-d_6) δ 145.35, 138.15, 121.44, 116.17, 114.14, 112.73, 67.21. HRMS-ESI (m/z): $[M+H]^+$ calcd. for $C_{14}H_{17}N_2O_2^+$, 245.1290; found, 245.1293.

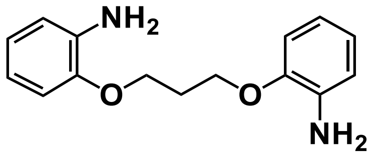

2-[3-(2-Aminophenoxy)propoxy]aniline

The product is obtained as pale yellow solid. ^{1}H NMR (400 MHz, $CDCl_3$) δ 6.80-6.79 (m, 2 H), 6.66-6.64 (m, 4 H), 6.50 (m, 2 H), 4.69 (s, 4 H), 4.13 (m, 4 H), 2.18 (m, 2 H); ^{13}C NMR (100 MHz, DMSO-d_6) δ 145.96, 138.24, 121.39, 116.62, 114.37, 112.11, 64.96, 29.44. HRMS-ESI (m/z): $[M+H]^+$ calcd. for $C_{15}H_{19}N_2O_2^+$, 259.1446; found, 259.1444.

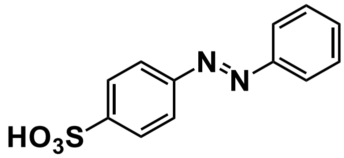

4-sulfonic-azobenzene

The product is obtained as orange solid. ^{1}H NMR (400 MHz, DMSO-d_6) δ 10.34 (s, 1 H), 7.82-7.80 (m, 4 H), 7.59-7.52 (m, 2 H), 6.97-6.94 (m, 2 H), 6.78-6.74 (m, 1 H); ^{13}C NMR (100 MHz, DMSO-d_6) δ 161.43, 152.53, 145.68, 130.95, 129.83, 125.31, 122.52, 119.31, 116.36, 115.68. HRMS-ESI (m/z): $[M+Na]^+$ calcd. for $C_{12}H_{10}N_2O_3SNa^+$, 285.0310; found, 285.0307.

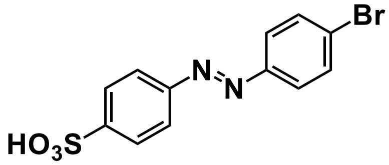

4-sulfonic-4’-(bromophenyl)azobenzene

The product is obtained as red solid. ^{1}H NMR (400 MHz, DMSO-d_6) δ 8.25-8.21 (d, 2 H), 8.03-7.99 (d, 2 H), 7.85-7.80 (m, 2 H), 7.67-7.64 (m, 2 H); ^{13}C NMR (100 MHz, DMSO-d_6) δ 156.32, 151.21, 148.77, 132.95, 127.40, 125.39, 125.24, 124.33, 124.29, 123.20, 123.11. HRMS-ESI (m/z): $[M+Na]^+$ calcd. for $C_{12}H_9BrN_2O_3SNa^+$, 362.9415; found, 362.9414.

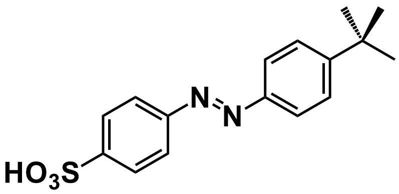

4-sulfonic-4’-(tertbutyl-phenyl)azobenzene

The product is obtained as orange solid. ^{1}H NMR (400 MHz, DMSO-d_6) δ 11.32 (s, 1 H), 7.92-7.90 (d, 2 H), 7.76 (d, 2 H), 7.61-7.59 (m, 2 H), 7.50-7.47 (m, 2 H), 1.34 (s, 12 H); ^{13}C NMR (100 MHz, DMSO-d_6) δ. 154.73, 152.11, 149.58, 142.65, 137.74, 131.31, 126.69, 122.72, 121.50, 118.22, 35.26, 31.54. HRMS-ESI (m/z): $[M+Na]^+$ calcd. for $C_{16}H_{18}N_2O_3SNa^+$, 341.0936; found, 341.0939.

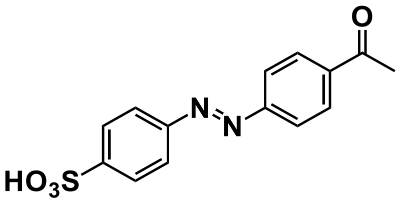

4-sulfonic-4’-(acetylphenyl)azobenzene

The product is obtained as yellow solid. ^{1}H NMR (400 MHz, DMSO-d_6) δ 8.25-8.20 (d, 2 H), 8.03-7.90 (m, 4 H), 7.70 (d, 2 H), 2.41 (s, 3 H); ^{13}C NMR (100 MHz, DMSO-d_6) δ. 157.14, 156.50, 147.69, 139.85, 129.25, 129.17, 125.43, 123.05, 122.95, 122.90, 122.88, 27.60. HRMS-ESI (m/z): $[M+Na]^+$ calcd. for $C_{14}H_{12}N_2O_4SNa^+$, 327.0416; found, 327.0417.

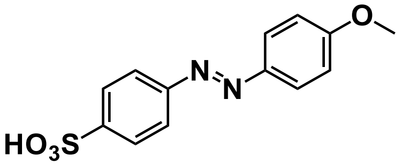

4-sulfonic-4’-(methoxyphenyl)azobenzene

The product is obtained as orange solid. ^{1}H NMR (400 MHz, DMSO-d_6) δ 8.17-8.14 (d, 2 H), 7.98-7.95 (d, 2 H), 7.68-7.66 (d, 2 H), 7.23-7.20 (d, 2 H), 3.65 (s, 3 H); ^{13}C NMR (100 MHz, DMSO-d_6) δ. 162.38, 156.51, 147.35, 142.77, 124.39, 124.35, 124.30, 123.19, 123.10, 117.33, 58.53. HRMS-ESI (m/z): $[M+Na]^+$ calcd. for $C_{13}H_{12}N_2O_4SNa^+$, 315.0416; found, 315.0418.

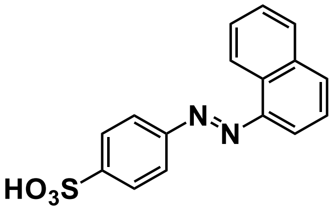

4-[2-(1-Naphthalenyl)diazinyl]benzenesulfonic acid

The product is obtained as dark red solid. ^{1}H NMR (400 MHz, DMSO-d_6) δ 9.02 (s, 1 H), 8.97 (d, 1 H), 8.25 (d, 1 H), 8,15-8.13 (m, 1 H), 8.05-8.00 (m, 2 H), 7.93-7.90 (m, 1 H), 7.71-7.62 (m, 4 H), 7.53-7.51 (m, 1 H); ^{13}C NMR (100 MHz, DMSO-d_6) δ. 151.11, 148.34, 138.20, 133.91, 129.50, 128.11, 124.92, 121.59, 116.27, 111.65, 108.08. HRMS-ESI (m/z): $[M+Na]^+$ calcd. for $C_{16}H_{12}N_2O_3SNa^+$, 335.0467; found, 335.0466.

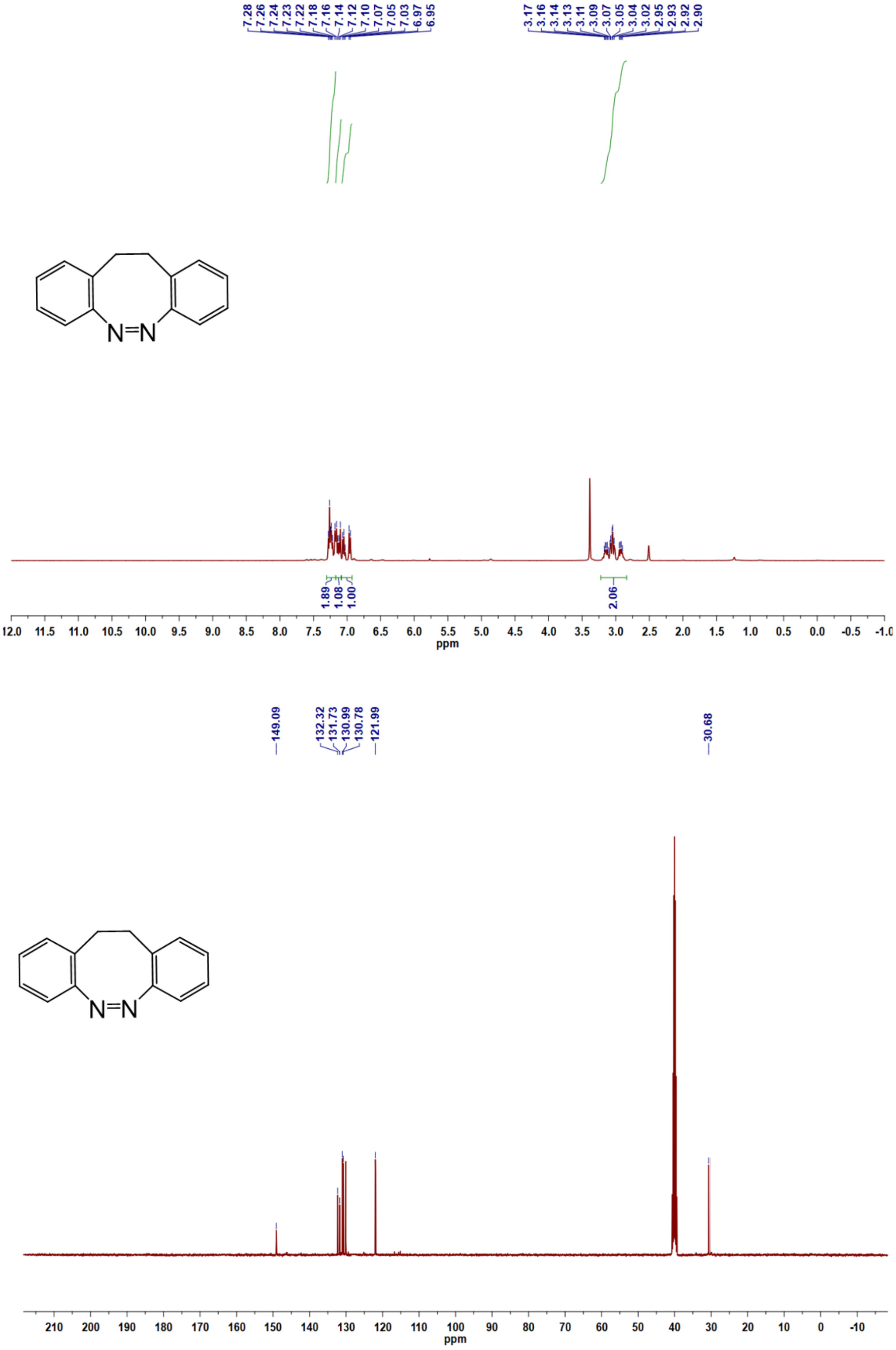

Supplementary Figure 57. NMR spectra of **6b**.

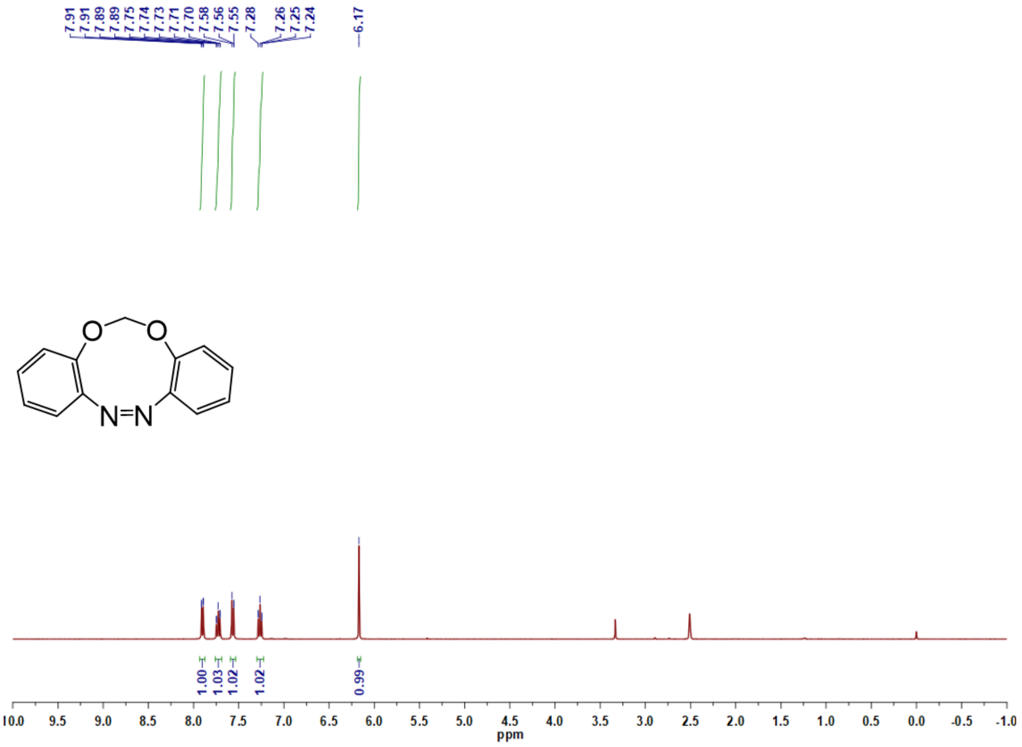

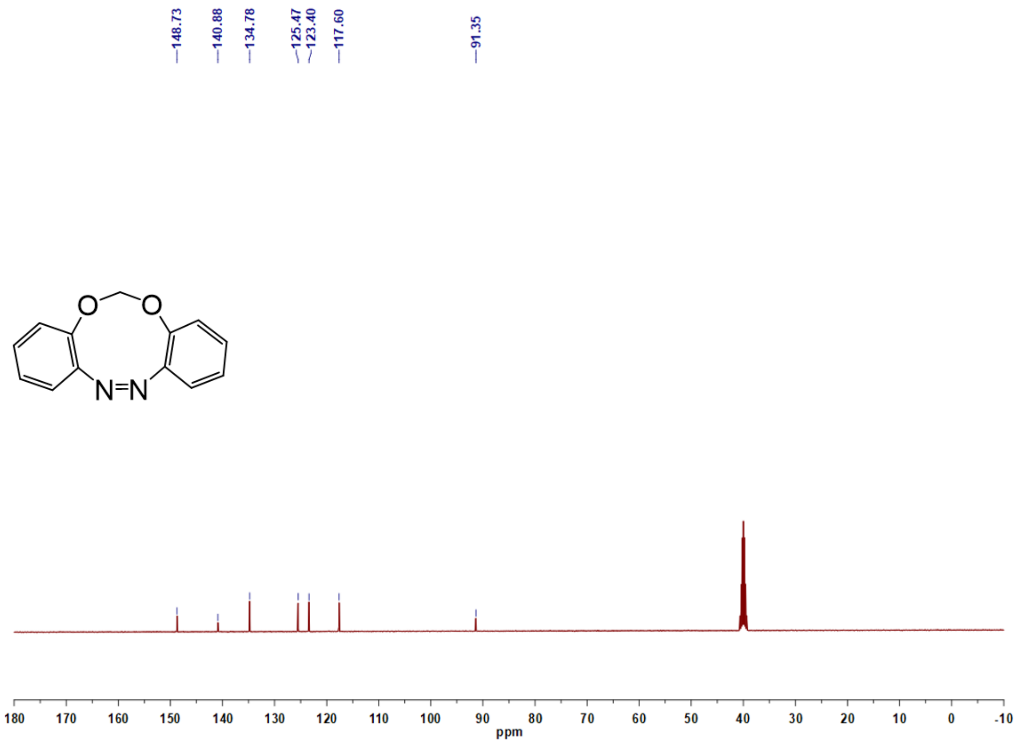

Supplementary Figure 58. NMR spectra of **7b**.

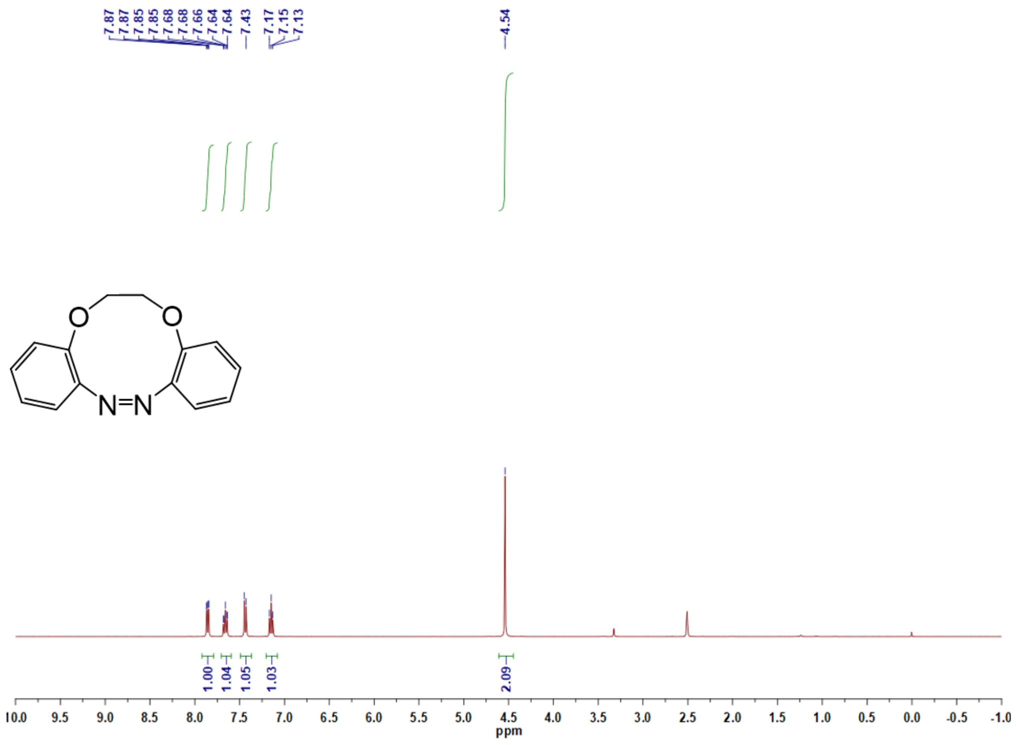

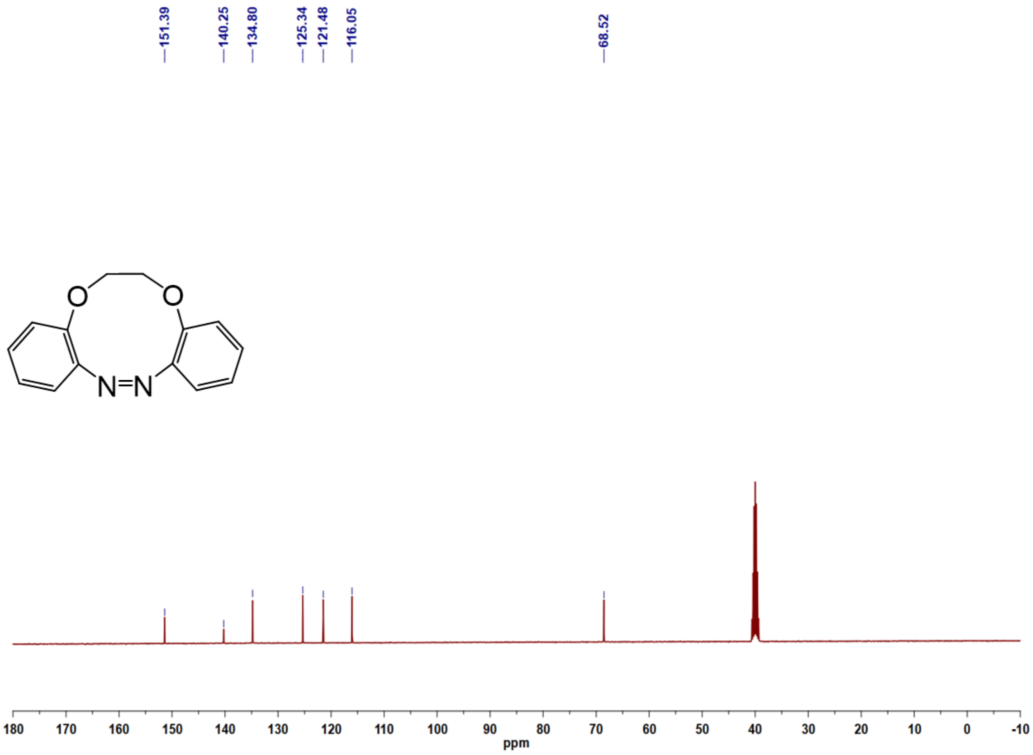

Supplementary Figure 59. NMR spectra of **8b**.

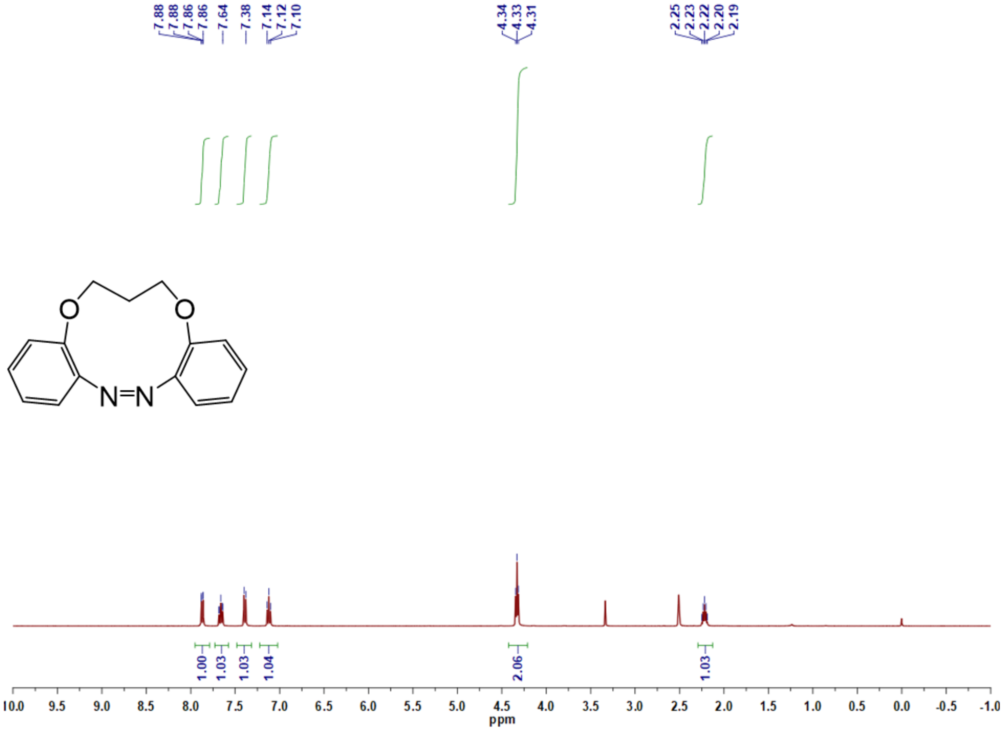

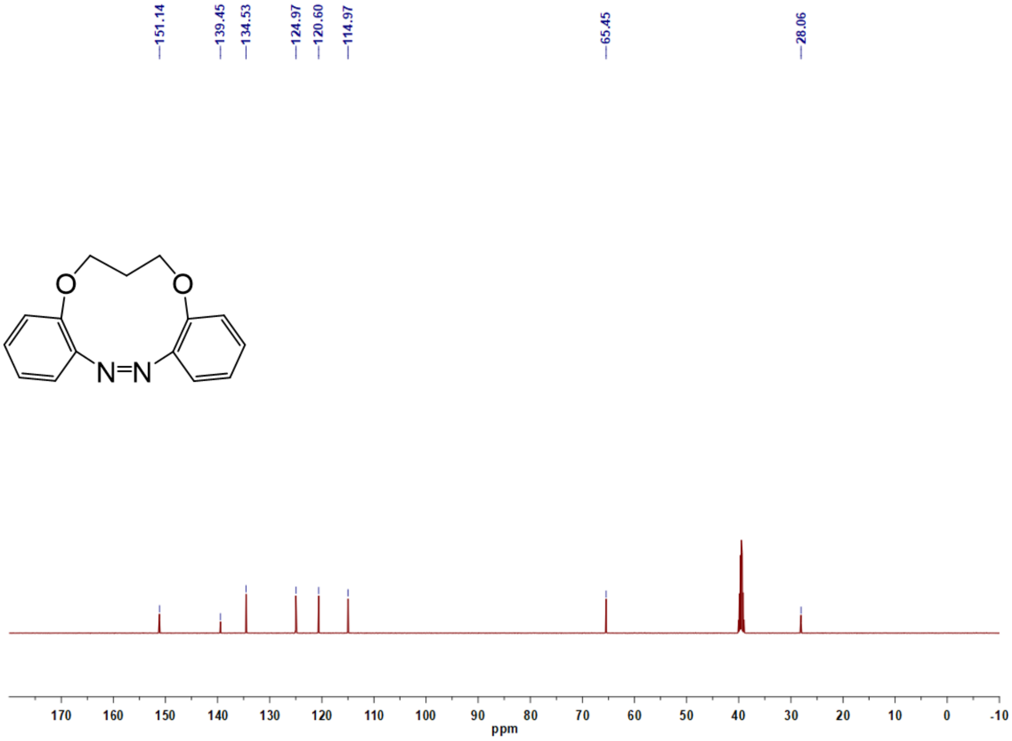

Supplementary Figure 60. NMR spectra of **9b**.

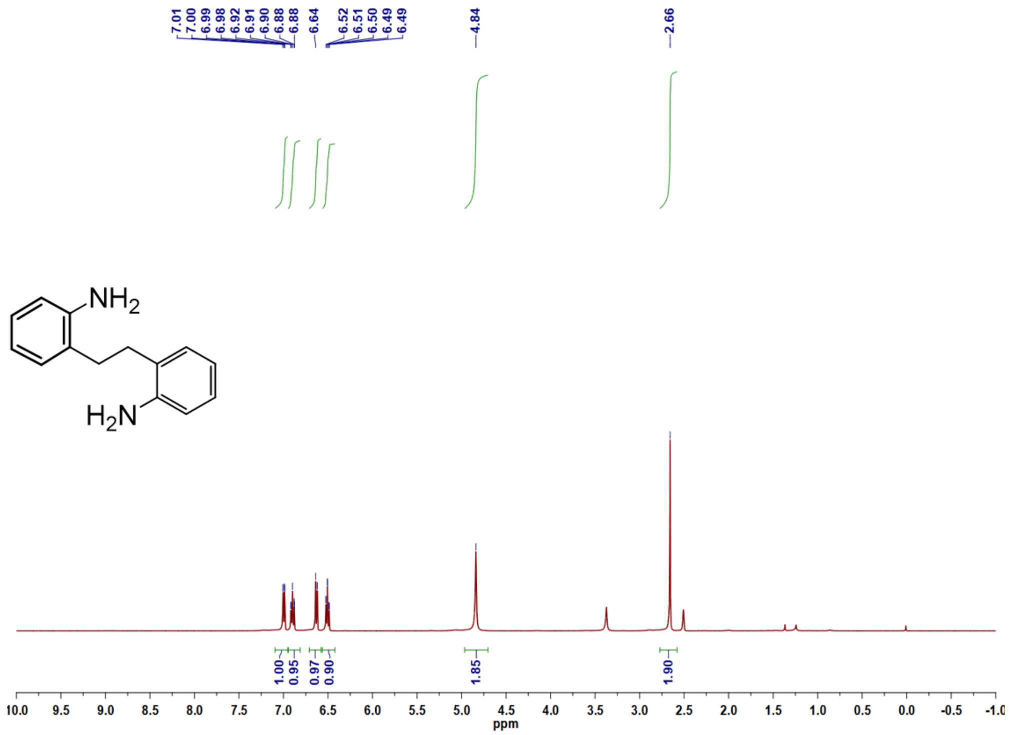

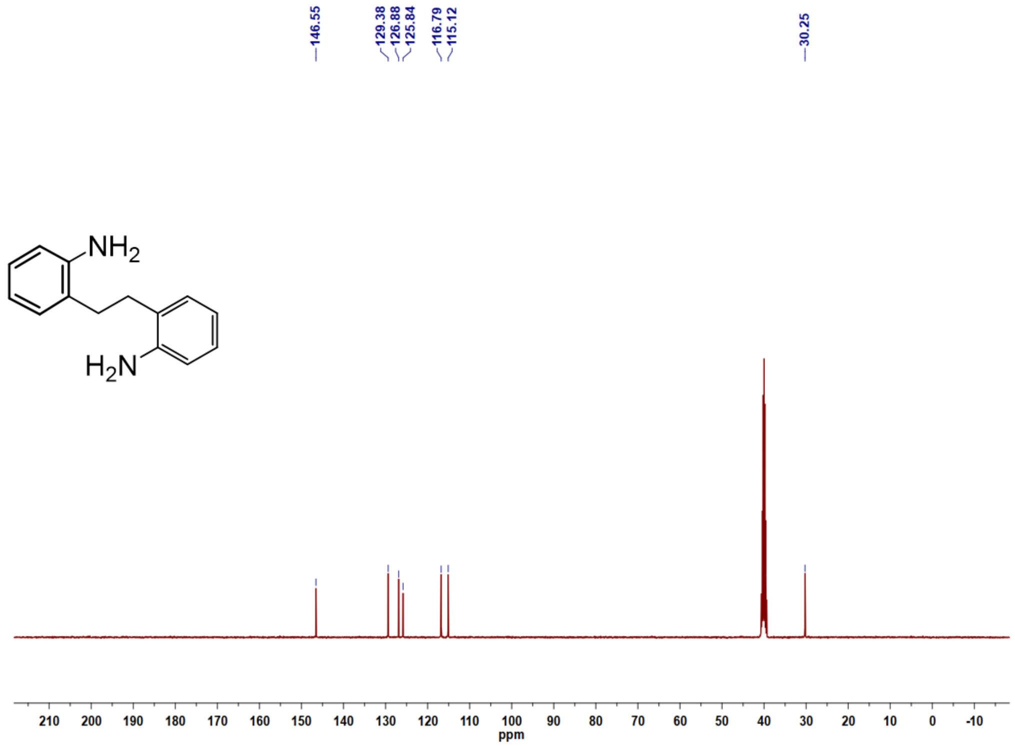

Supplementary Figure 61. NMR spectra of **6a**.

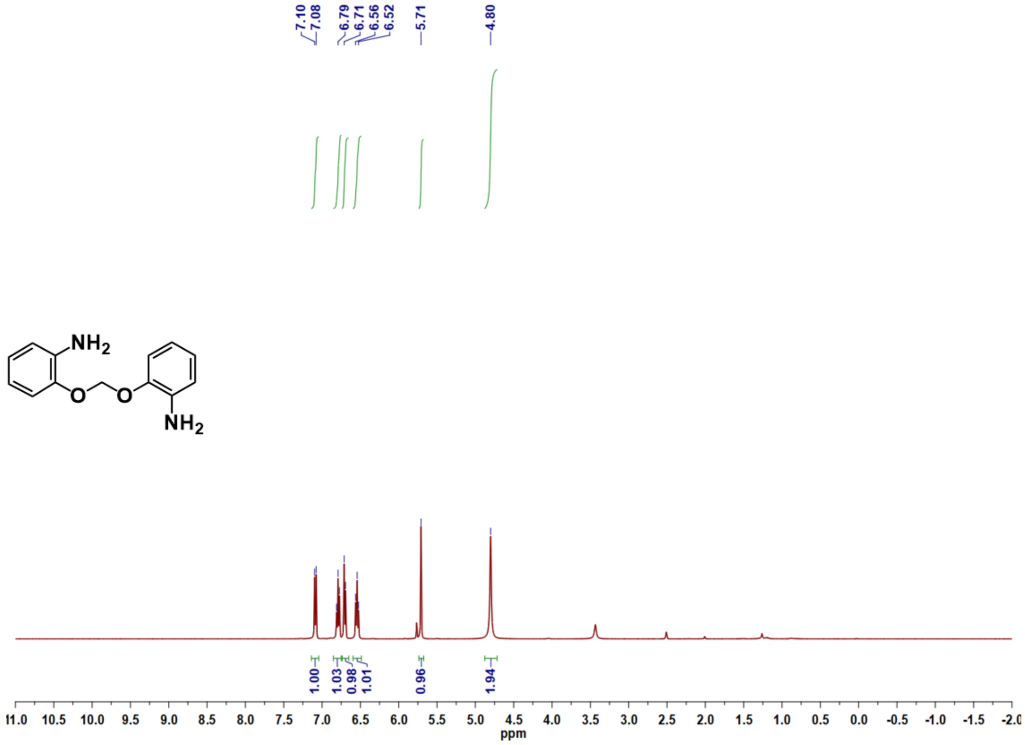

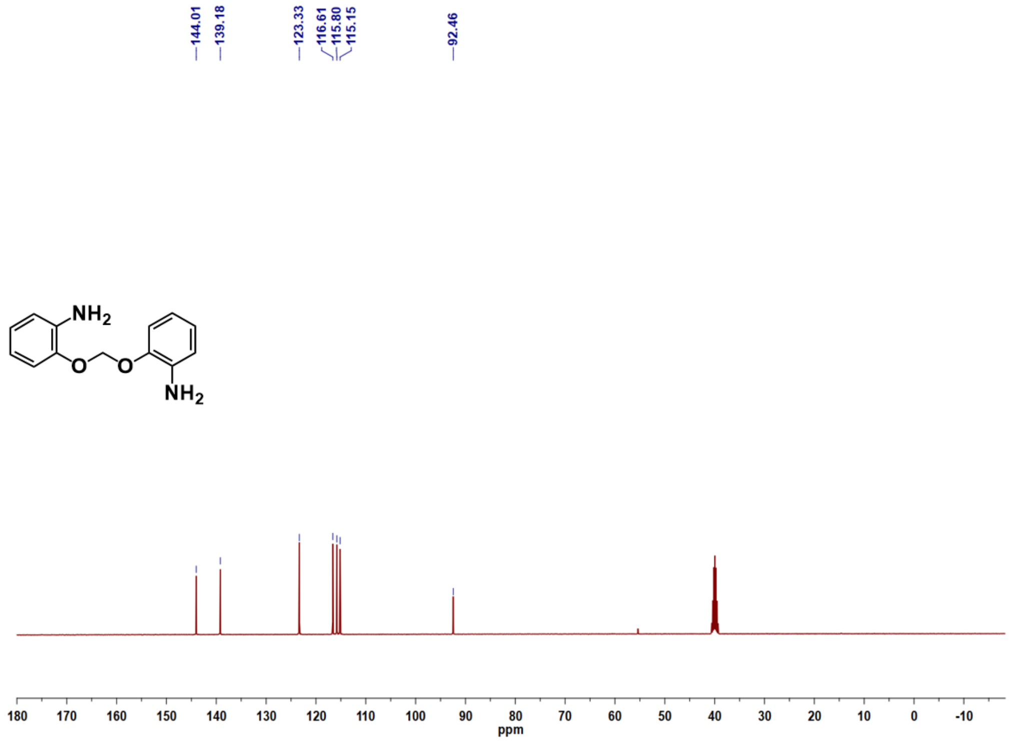

Supplementary Figure 62. NMR spectra of **7a**.

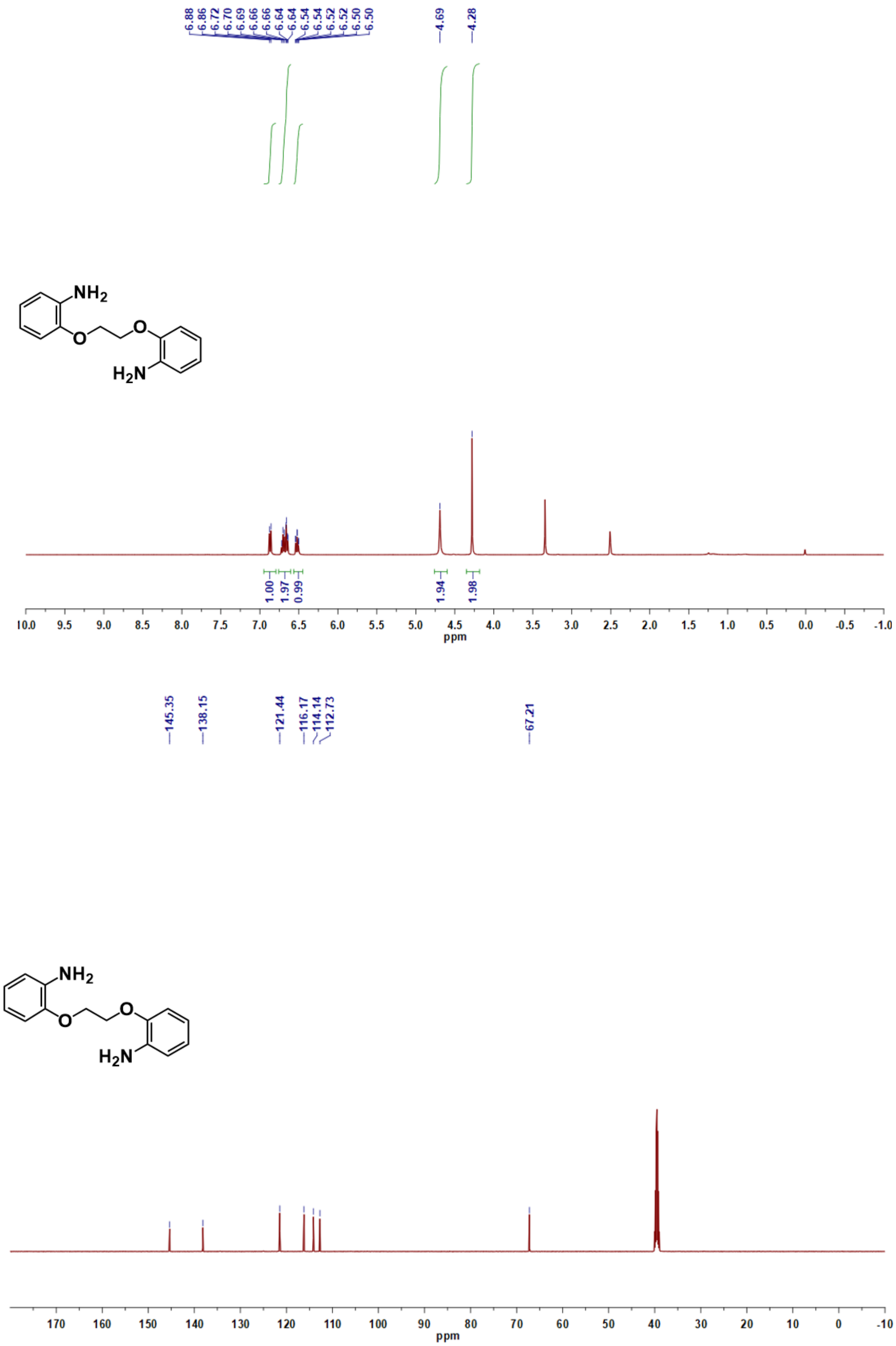

Supplementary Figure 63. NMR spectra of **8a**.

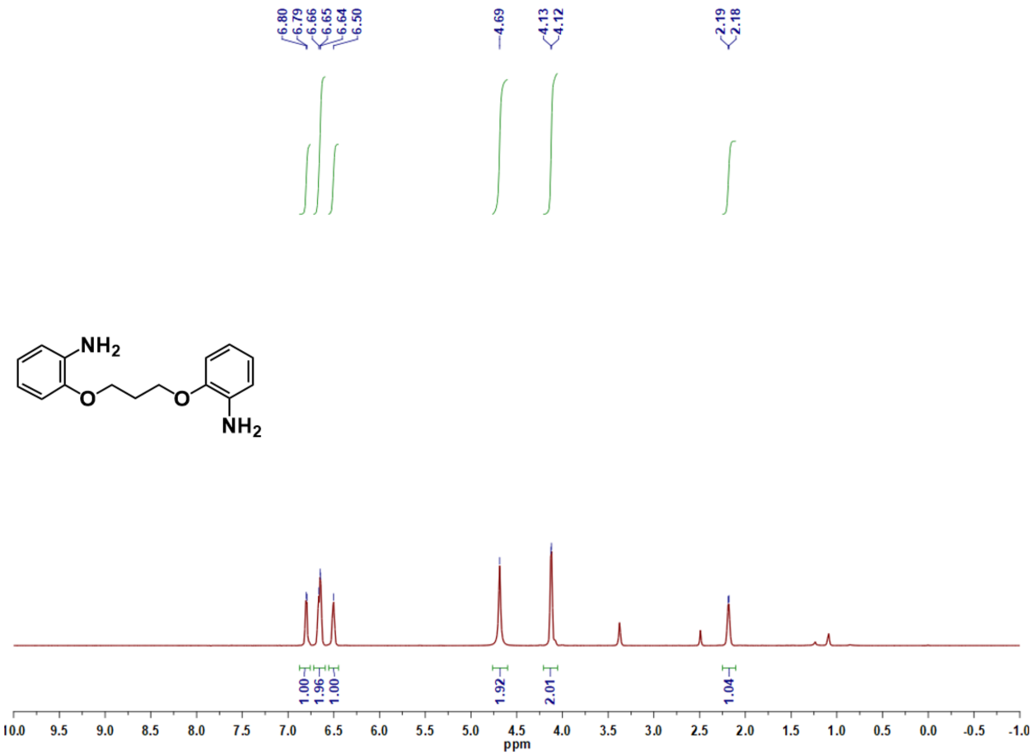

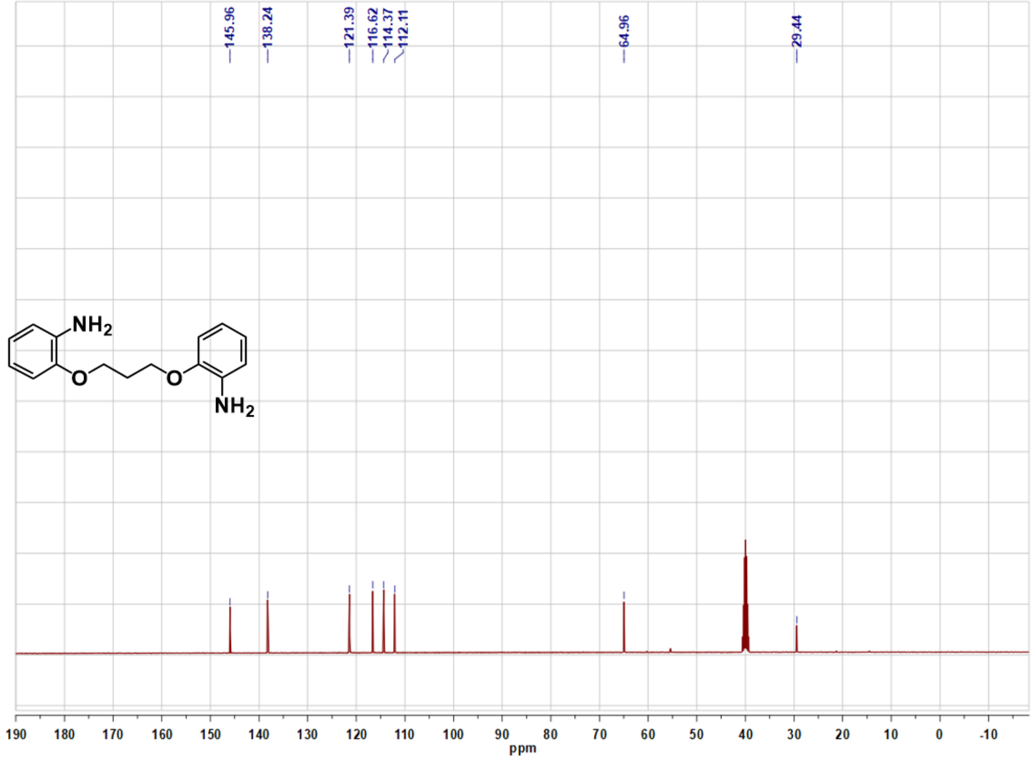

Supplementary Figure 64. NMR spectra of **9a**.

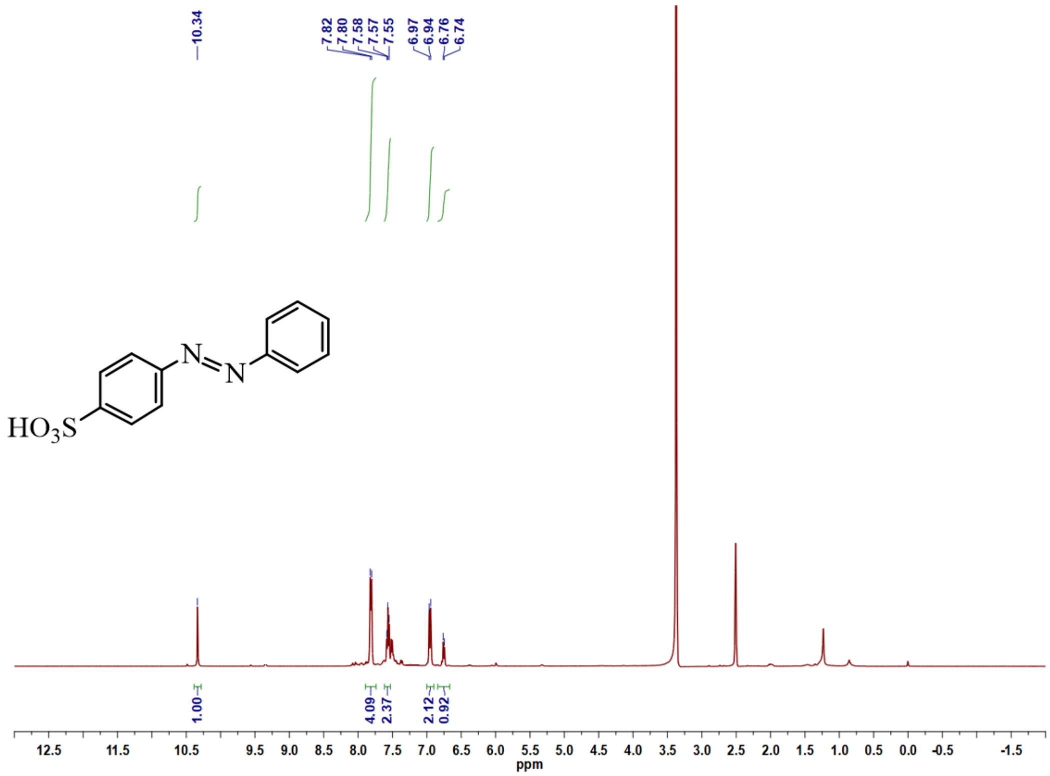

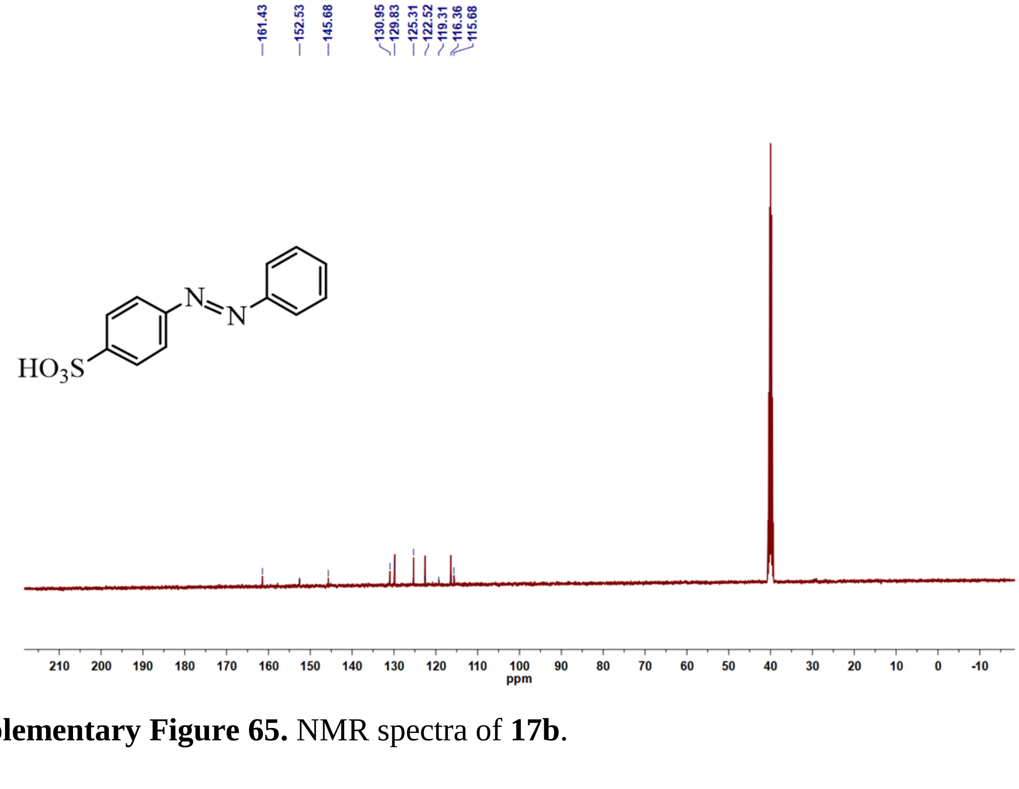

Supplementary Figure 65. NMR spectra of **17b**.

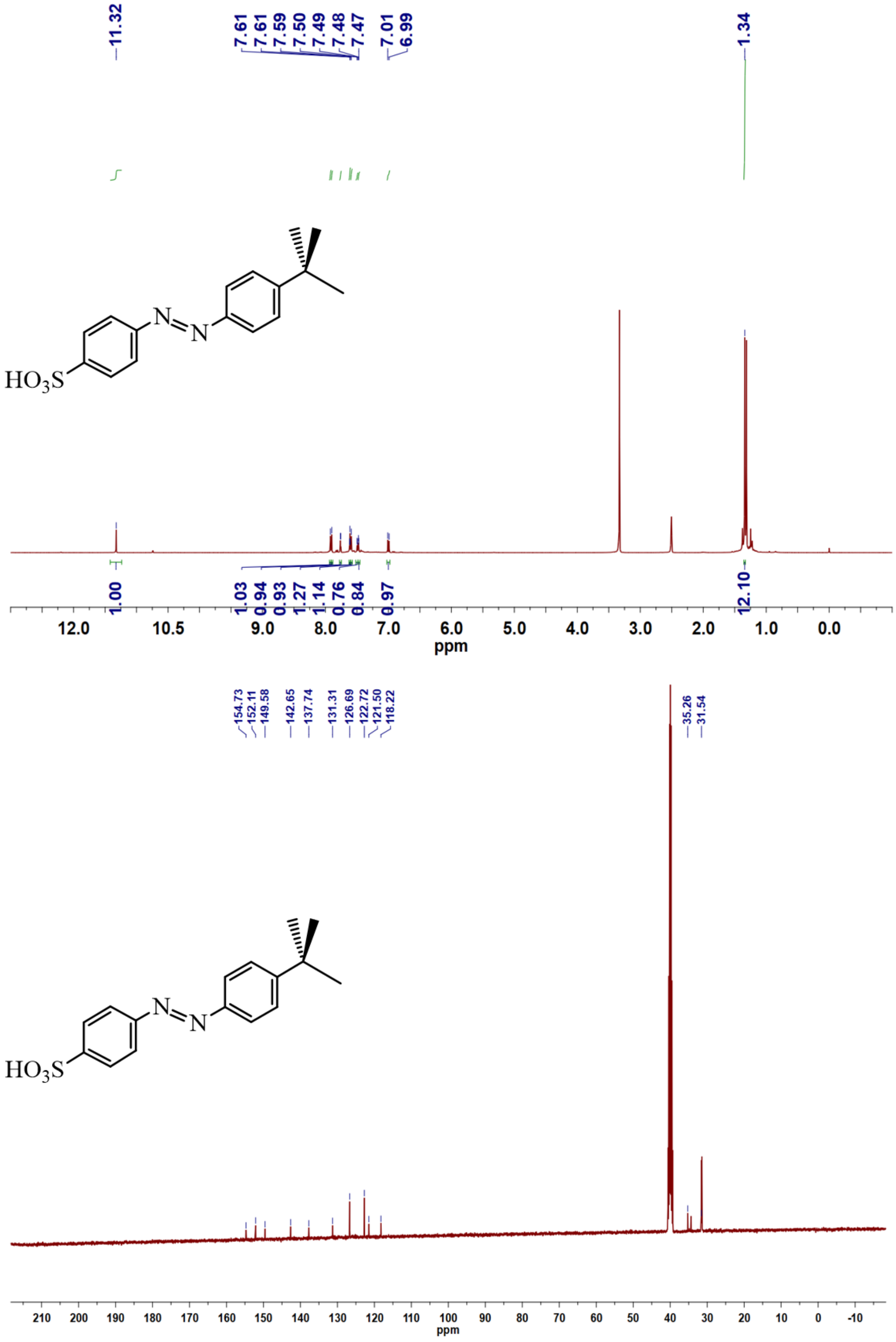

Supplementary Figure 66. NMR spectra of **19b**.

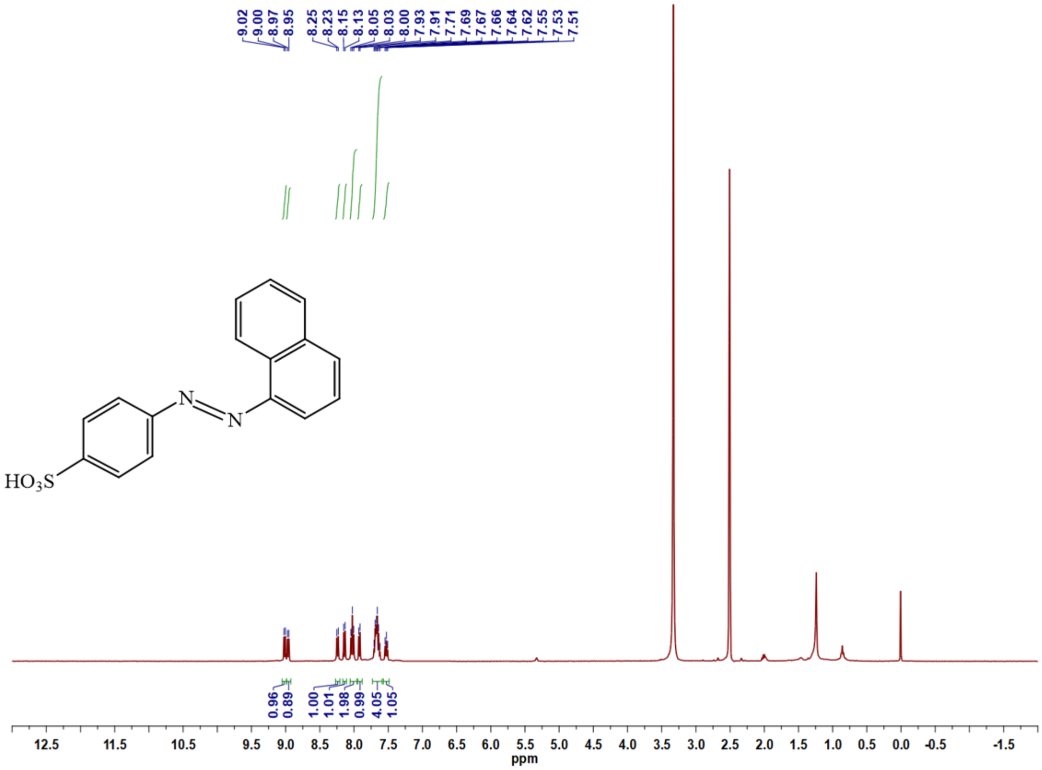

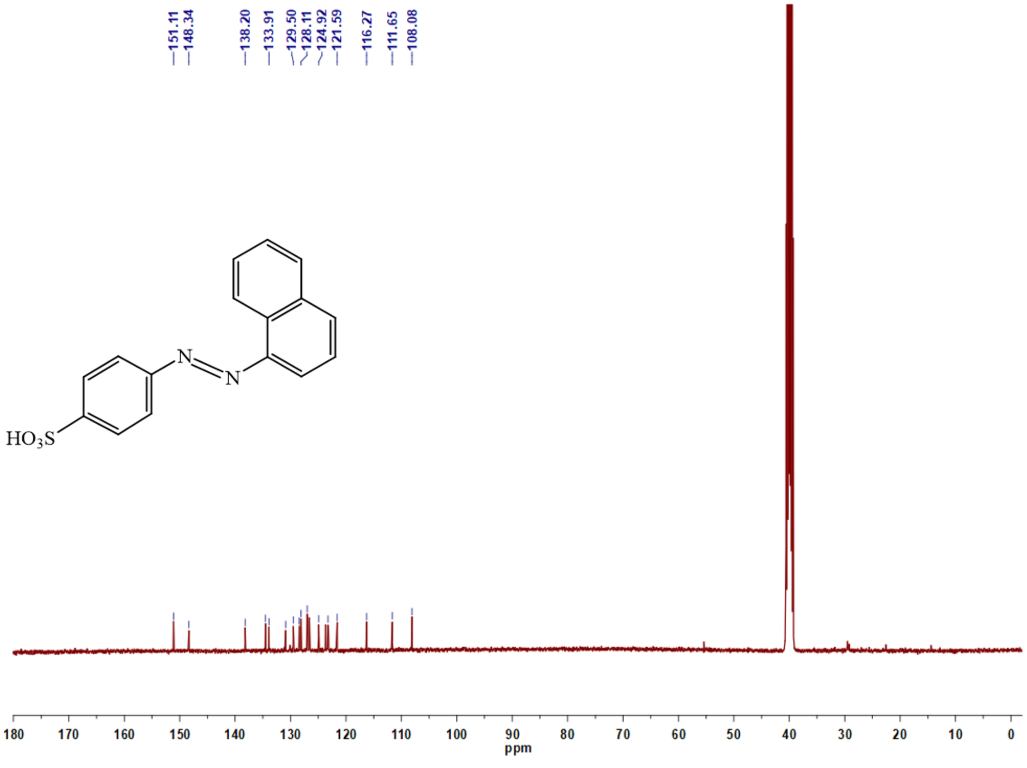

Supplementary Figure 67. NMR spectra of **22b**.

Supplementary References

1. Moormann, w., Langbehn, D., & Herges, R. Solve-free synthesis of diazocine. *Synthesis.* **49**, 3471-3475 (2017).
2. Sakate, S., Kamble, S., Chikate, R., & Rode, C. Facile one-pot synthesis of aliphatic bridged diaryloxy compounds, cyclic and crown ethers under mild conditions. *Supramolecular Chemistry.* **29**, 462-470 (2017).
3. SMART, Data collection software (version 5.629) (Bruker AXS Inc., Madison, WI, 2003).
4. SAINT, Data reduction software (version 6.54) (Bruker AXS Inc., Madison, WI, 2003).
5. Sheldrick, G. M. SHELXTL97, Program for Crystal Structure Solution (University of Göttingen: Göttingen, Germany, 1997).
6. Prakash, B. D. & We, Y. C. A fully automated iterative moving averaging (AIMA) technique for baseline correction. *Analyst*, **136**, 3130–3135 (2011).
7. Bao, Q., Feng, J., Chen, F., Mao, W., Liu, Z., Liu, K. & Liu, C. A new automatic baseline correction method based on iterative method. *J Magn Reson*, **218**, 35-43 (2012).
